# Patient-level performance evaluation of a smartphone-based malaria diagnostic application

**DOI:** 10.1186/s12936-023-04446-0

**Published:** 2023-01-27

**Authors:** Hang Yu, Fayad O. Mohammed, Muzamil Abdel Hamid, Feng Yang, Yasmin M. Kassim, Abdelrahim O. Mohamed, Richard J. Maude, Xavier C. Ding, Ewurama D.A. Owusu, Seda Yerlikaya, Sabine Dittrich, Stefan Jaeger

**Affiliations:** 1 Lister Hill National Center for Biomedical Communications, National Library of Medicine, National Institutes of Health, MD Bethesda, USA; 2 Department of Parasitology and Medical Entomology, Institute of Endemic Diseases, Medical Campus, University of Khartoum, Khartoum, Sudan; 3 Department of Biochemistry, Faculty of Medicine, University of Khartoum, Khartoum, Sudan; 4 Mahidol Oxford Tropical Medicine Research Unit, Faculty of Tropical Medicine, Mahidol University, Bangkok, Thailand; 5 Centre for Tropical Medicine and Global Health, Nuffield Department of Medicine, University of Oxford, Oxford, UK; 6 Harvard TH Chan School of Public Health, Harvard University, Boston, USA; 7 FIND, Geneva, Switzerland; 8 Department of Medical Laboratory Sciences, School of Biomedical and Allied Health Sciences, College of Health Sciences, University of Ghana, Accra, Ghana

**Keywords:** Malaria microscopy, Computer-aided diagnosis, Automated screening, Machine learning, Field testing, Smartphone application

## Abstract

**Background:**

Microscopic examination is commonly used for malaria diagnosis in the field. However, the lack of well-trained microscopists in malaria-endemic areas impacted the most by the disease is a severe problem. Besides, the examination process is time-consuming and prone to human error. Automated diagnostic systems based on machine learning offer great potential to overcome these problems. This study aims to evaluate Malaria Screener, a smartphone-based application for malaria diagnosis.

**Methods:**

A total of 190 patients were recruited at two sites in rural areas near Khartoum, Sudan. The Malaria Screener mobile application was deployed to screen Giemsa-stained blood smears. Both expert microscopy and nested PCR were performed to use as reference standards. First, Malaria Screener was evaluated using the two reference standards. Then, during post-study experiments, the evaluation was repeated for a newly developed algorithm, PlasmodiumVF-Net.

**Results:**

Malaria Screener reached 74.1% (95% CI 63.5–83.0) accuracy in detecting *Plasmodium falciparum* malaria using expert microscopy as the reference after a threshold calibration. It reached 71.8% (95% CI 61.0–81.0) accuracy when compared with PCR. The achieved accuracies meet the WHO Level 3 requirement for parasite detection. The processing time for each smear varies from 5 to 15 min, depending on the concentration of white blood cells (WBCs). In the post-study experiment, Malaria Screener reached 91.8% (95% CI 83.8–96.6) accuracy when patient-level results were calculated with a different method. This accuracy meets the WHO Level 1 requirement for parasite detection. In addition, PlasmodiumVF-Net, a newly developed algorithm, reached 83.1% (95% CI 77.0–88.1) accuracy when compared with expert microscopy and 81.0% (95% CI 74.6–86.3) accuracy when compared with PCR, reaching the WHO Level 2 requirement for detecting both *Plasmodium falciparum* and *Plasmodium vivax* malaria, without using the testing sites data for training or calibration. Results reported for both Malaria Screener and PlasmodiumVF-Net used thick smears for diagnosis. In this paper, both systems were not assessed in species identification and parasite counting, which are still under development.

**Conclusion:**

Malaria Screener showed the potential to be deployed in resource-limited areas to facilitate routine malaria screening. It is the first smartphone-based system for malaria diagnosis evaluated on the patient-level in a natural field environment. Thus, the results in the field reported here can serve as a reference for future studies.

**Supplementary Information:**

The online version contains supplementary material available at 10.1186/s12936-023-04446-0.

## Background

Microscopic examination of Giemsa-stained blood films is a primary diagnostic tool for malaria case management [1]. However, manual microscopy is time-consuming and error-prone. Additionally, training qualified personnel comes with a high cost [2, 3]. To address this issue, researchers have spent effort in recent years to automate this process [4].

One approach is to fully automate microscopic examination, which typically involves building a hardware device that can streamline the process from the imaging step to the final diagnosis. Delahunt et al.proposed Autoscope [5], an automated digital microscope coupled with computer vision and machine learning algorithms. It can diagnose *Plasmodium falciparum* malaria by analysing a Giemsa-stained thick smear. Later, this prototype system evolved to a more advanced version and was renamed EasyScan Go [6, 7], adding functions to diagnose non-*P. falciparum* species and an algorithm for thin smear analysis. Several other groups [8–10] also proposed systems with similar hardware designs. EasyScan Go is taking the lead compared to others in that their algorithms can perform patient-level malaria diagnosis in all aspects, including parasite detection, quantitation, and species identification. They have evaluated their system based on the slide set that the World Health Organization (WHO) provides for external competence assessment of malaria microscopists and conducted field evaluation [6, 7].

Another approach is to use a semi-automated method that automates only part of microscopy, such as the field of views (FoVs) analysis, with a camera or smartphone device mounted to the microscope for imaging. A semi-automated system requires more human intervention than a fully automated system. However, it usually consists of less sophisticated hardware components that can make it more affordable and, in some cases, easier to deploy. These are critical features since most malaria-endemic regions are resource-limited areas. Several publications have proposed semi-automated systems [11–14]; however, none of those has been evaluated in the field for patient-level diagnosis.

The system discussed in the following falls into the semi-automated group. The core is Malaria Screener [15], an Android mobile application that automates parasite detection using machine learning and computer vision algorithms. This application includes an image acquisition module, a parasite detection module that can detect malaria parasites by analysing the FoVs of microscopy, and a data management module that saves and can export diagnostic records. This study reports the performance of the semi-automated mobile system during a field evaluation in Sudan.

## Methods

The performance evaluation was conducted during a case–control study organized by FIND (global alliance for diagnostics) [16] with the help of the Institute of Endemic Diseases, University of Khartoum, Sudan (IEND), to evaluate the Malaria Screener software developed by the National Library of Medicine (NLM) at the National Institutes of Health (NIH). Patients were recruited at two primary hospitals in Sudan, one in the Alsororab (SOR) area and another one in the Gezira Slanj (GS) area, 40 and 50 km north of Khartoum where *P. falciparum* and *Plasmodium vivax* are endemic. The patients were recruited during the second malaria season between October 2020 and March 2021.

Sample size calculation was performed according to [17]. It was estimated that 100 patients positive for malaria (cases) by on-site microscopy (approx. 1.1xN) would need to be recruited for the evaluation to obtain a reliable estimate of the expected sensitivity, with 95% power of getting a 95% confidence interval of ± 10% or less, while allowing for procedural errors in 10% of all cases. Furthermore, it was estimated that 90 patients negative for malaria (controls) by on-site microscopy (approx. 1.4xN) would need to be recruited for the evaluation to obtain a reliable estimate of the expected specificity with 95% power of getting a 95% confidence interval of ± 10% or less while allowing for procedural errors in 10% of all controls.

**Fig. 1:**
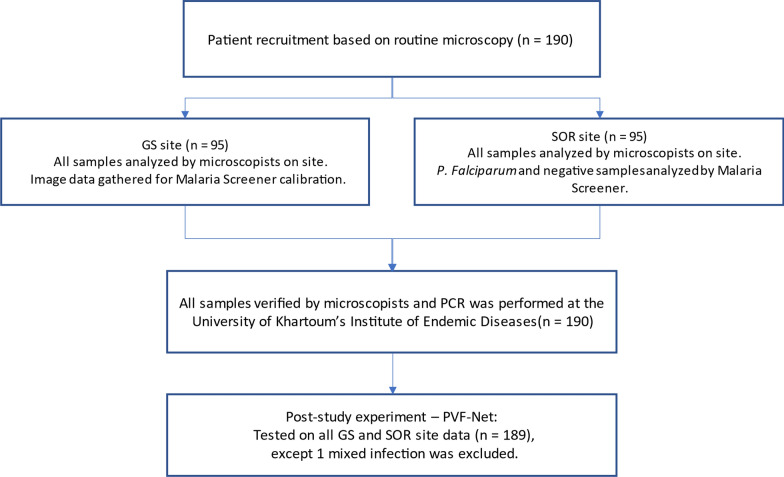
Flow chart of the study procedures. For Malaria Screener, *P. vivax* samples were excluded since it can only process P*. falciparum* malaria. For PVF-Net, a newly developed deep learning-based algorithm, one mixed infection sample was excluded since it cannot process mixed infection

Patients were enrolled consecutively until reaching the calculated numbers (190 patients in total, 95 from each site). Patients were five years of age and older. Patients with symptoms and signs of severe disease or comorbidities such as central nervous system or cardiovascular disease, as defined by World Health Organization (WHO) guidelines, were excluded, as were those who had received anti-malarial treatment in the four weeks before enrollment. Patients were enrolled after signing informed consent documents. Finger-prick blood samples were collected by a capillary tube to prepare blood smears, and dried blood spots (DBS) were prepared for PCR analysis. Figure 1 describes the procedures that were performed during this study.

### Manual light microscopy

Light microscopy was performed for malaria diagnosis, species identification, and parasite counting. Blood smears were stained by a 3% freshly prepared Giemsa solution for 45 min before washing and air-dried for one hour at room temperature according to WHO standard procedures [18]. The slides were first examined at the hospitals of each region by site microscopists and later verified by a second microscopist at the University of Khartoum’s Institute of Endemic Diseases. The Obare method calculator [19] was used to determine whether the two readings agreed. A third expert microscopy reading was performed in the event of a discrepancy. All microscopists were WHO Level 1 expert microscopists.

### Blood film examination using Malaria Screener

**Fig. 2:**
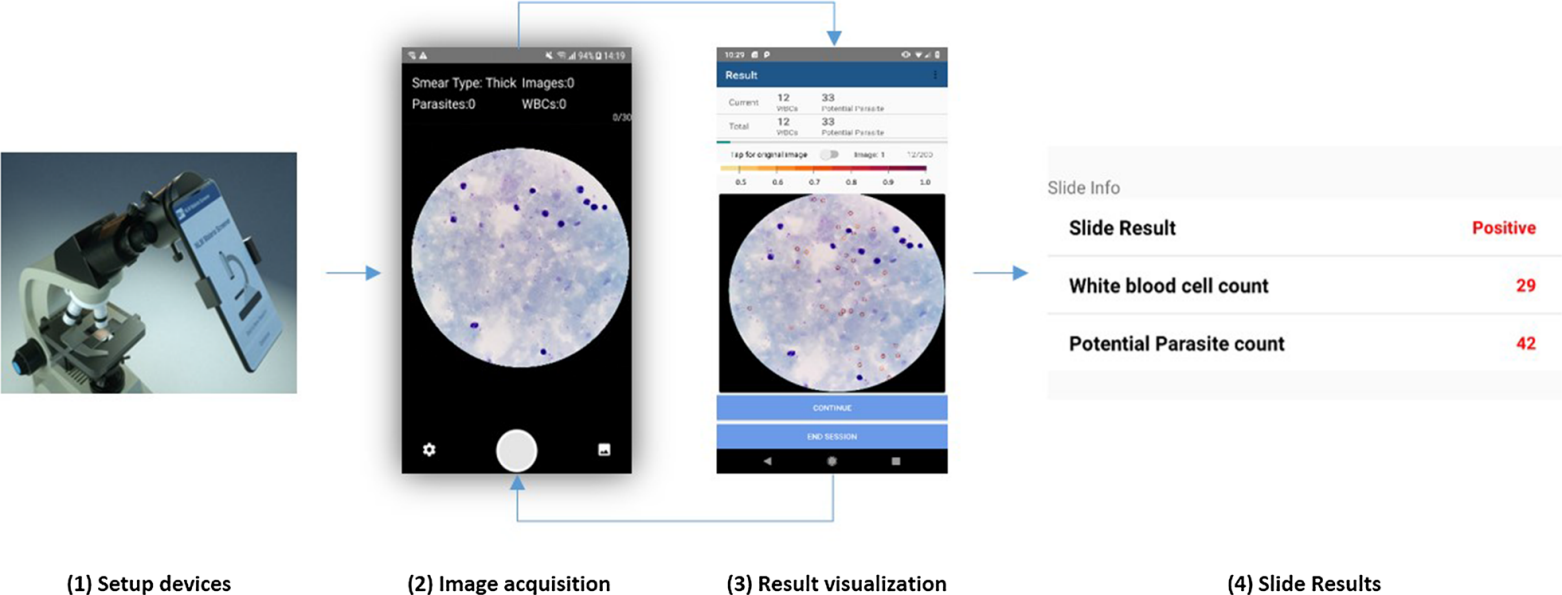
System workflow overview

Malaria Screener-assisted microscopy requires mounting a smartphone onto the eyepiece of a microscope. Each site was equipped with a Samsung Galaxy A10 smartphone and an Olympus CX23 microscope. Malaria Screener (version 1.6.6) was downloaded from Google Play Store and installed on each device. While screening a blood smear, a microscopist looked for suitable FoVs for the app to capture, and the app instantly processed the images on the phone. These two actions were repeated until a user-specified WBC threshold was met (This threshold is 200 by default and can be changed by the user). Then, the app displayed the diagnostic results and saved the diagnosis and the captured image data, which were later exported to an external database. A diagram of the workflow is shown in Fig. 2. More details regarding the software can be found in previous publications [15, 20, 21]. Before the study, a training session was provided to the microscopists to teach them how to use the app. This includes attaching the smartphone to the eyepiece with the adapter, selecting proper FoVs, and adjusting settings, for example. A user manual was also provided for future reference (The user manual is attached as Additional file 1).

Slides from the GS site were used to fine-tune the system parameters. Fine-tuning mainly consisted of analysing the receiver operating characteristic (ROC) to find a confidence threshold leading to the highest patient-level accuracy. Then, Malaria Screener was tested on SOR site samples by a microscopist on site. However, the results for *P. vivax* samples were excluded because the algorithm in Malaria Screener was only ready to be used for *P. falciparum* malaria.

### Blood film examination using PVF-Net (Post-study experiment)

After the study, an improved version of the diagnostic algorithm was developed. This new framework is called PlasmodiumVF-Net [22] (PVF-Net). Its algorithm design is different from Malaria Screener. It can detect both *P. falciparum* and *P. vivax* infections. However, the mechanism to aggregate results from object to image level is like Malaria Screener in that PVF-Net averages probabilities, although it manages different thresholds for *P. falciparum* and *P. vivax*. PVF-Net was trained and calibrated using a dataset from Bangladesh, as described by Kassim et al. [22]. Images collected from the Sudan blood slide samples were re-examined using PVF-Net in a post-study experiment.

### DNA extraction and nested PCR

DNA extraction was performed from DBS using a QIAamp DNA extraction kit following the manufacturer’s instructions (Qiagen, Germany). Nested PCR was performed to identify malaria species following Snounou et al. using previously published primers [23]. In each assay, a positive control of *P. falciparum* and *P. vivax* [DNA sample from *P. falciparum* (3D7 strain) and *P. vivax* (Sudanese isolate)] was included*,* and a negative control (DNA sample negative for malaria) was included as well.

### Patient-level diagnosis

An object-level (parasite) diagnosis was performed first within each blood smear image; then, results were combined across images to determine whether a patient was positive or negative.

The deep learning classifier provided a confidence score for each detection in an image. The average score of all detections was computed and used as the confidence score of an image (Eq. 1). Then, the average score of all images was taken as the confidence score of the corresponding smear, meaning patient (Eq. 2). If the patient score exceeds a certain threshold, then the patient is considered positive; otherwise, the patient is considered negative. Slides from the GS site were used for fine-tuning to determine this threshold.

1 $${Conf}_{img}= \frac{1}{{n}_{1}}\sum_{i=1}^{{n}_{1}}{p}_{i}$$

2 $${Conf}_{patient}= \frac{1}{{n}_{2}}\sum_{i=1}^{{n}_{2}}{Conf}_{img}$$

p_i_ prediction likelihood of parasite candidate.

n_1_ number of parasite candidates in the image.

Conf_img_ confidence score of the image.

n_2_ number of images captured for the smear.

Conf_patient_ confidence score of the smear.

### Evaluation

System performance was evaluated on the patient-level based on accuracy, sensitivity, and specificity. Microscopy and PCR were used as reference standards for evaluation, where decisions of WHO Level 1 microscopists were used for microscopy.

## Results

### Sudan data statistics

A total of 380 slides were prepared with blood collected from the 190 patients of the two participating sites. 103 (54.2%) patients were male, and 87 (45.8%) were female. The average age of the patients was 29.8, with a standard deviation of 15.6. Specifically, two slides were collected for each patient, one for analysis and another for backup, containing both a thin and a thick smear. Of the 190 slides used for analysis, 100 (52.6%) tested positive by expert microscopy, and 90 (47.4%) tested negative. Among the positive slides, 61 were *P. falciparum*, 38 were *P. vivax*, and one was a *P. falciparum* + *P. vivax* mixed infection.

**Fig. 3:**
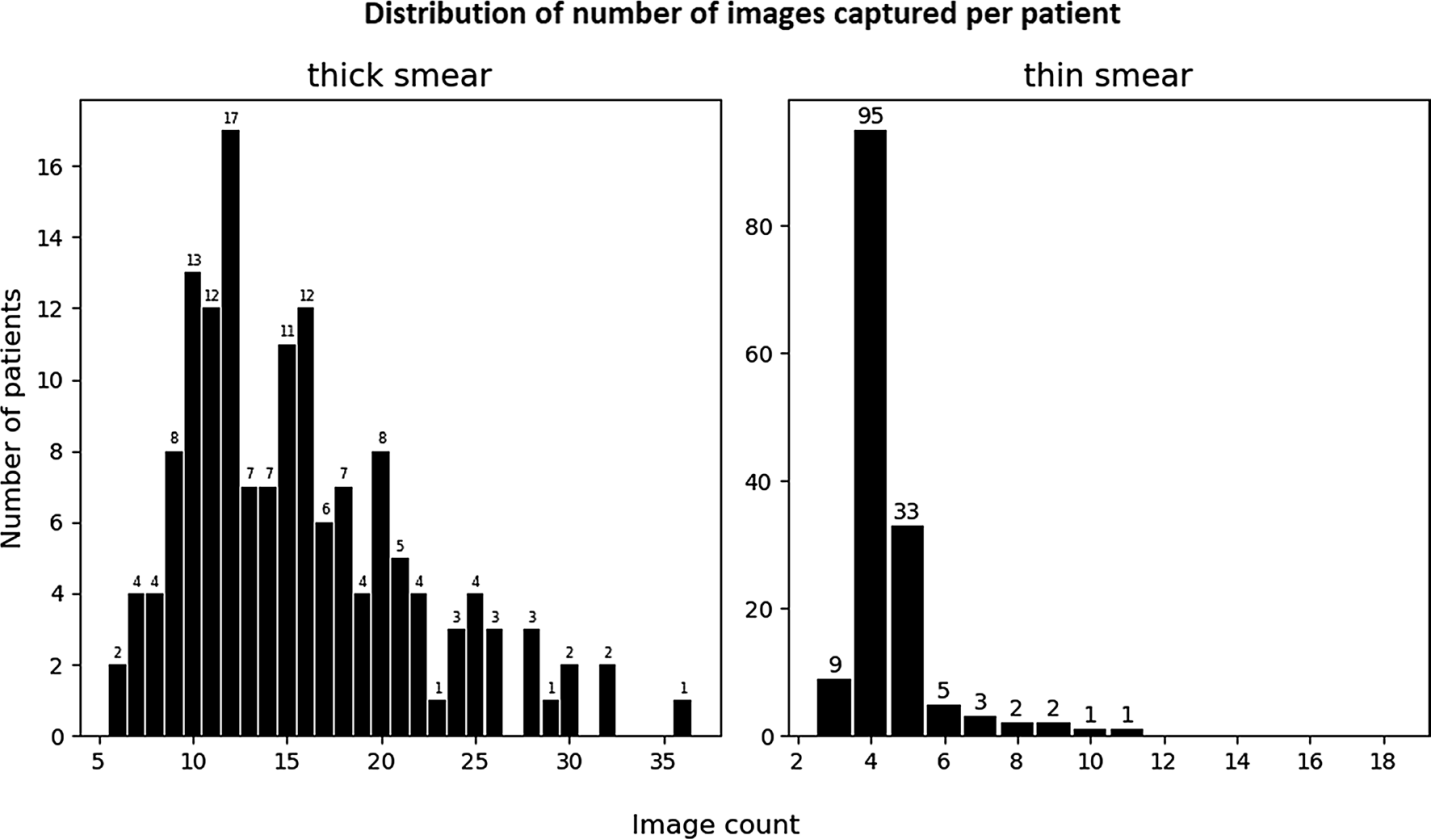
Histogram distribution of patient image counts

A total of 2944 images were collected from thick blood smears (15.5 images/patient), and 875 images were collected from thin blood smears (4.6 images/patient). Details about the image collections can be found in Table 1. More images were gathered from thick smears because the minimum WBC count threshold used (1000) was high compared to the WBC concentration of the slides. Approximately 10 to 20 images were collected for each thick smear, and around 4 to 6 images were gathered for each thin smear, as shown in Fig. 3.

**Table 1:** Overview of the dataset collected in Sudan

		Number of slides	Thick smear images	Thin smear images
Site GS	P. Falciparum	21	395	90
	P. Vivax	28	409	125
	Negative	45	705	197
	Mixed infection of P. F. & P. V	1	28	4
	Sum	95	1537	416
Site SOR	P. Falciparum	40	609	197
	P. Vivax	10	124	53
	Negative	45	674	209
	Sum	95	1407	459

### Evaluation using expert microscopy as a reference

Malaria Screener was tested on SOR *P. falciparum* and negative samples only. This part of the dataset includes 85 patients (40 positive patients and 45 negative patients). Meanwhile, during post-study experiments, PVF-Net was tested on both *P. falciparum* and *P. vivax* species from both sites, including 189 patients (99 positive and 90 negative patients). The evaluation results are listed in Table 2.

**Table 2:** Malaria Screener and PVF-Net evaluation using microscopy as a reference

Evaluation Metrics	Accuracy, % (95% CI)	Sensitivity, % (95% CI)	Specificity, % (95% CI)
Index tests			
Malaria Screener (85 patients)	74.1 (63.5—83.0)	100 (91.2—100)	51.1 (35.8—66.3)
PVF-Net (189 patients)	83.1 (77.0—88.1)	86.9 (78.6—92.8)	78.9 (69.0—86.8)

#### Parasite detection with Malaria Screener (P. falciparum only)

Malaria Screener achieved 74.1% (95% CI 63.5–83.0) accuracy in detecting *P. falciparum* malaria through thick smears. It correctly saw whether malaria is present in 63 of 85 patients. This result meets the WHO Level 3 criterion in the parasite detection category [24]. The application has a high sensitivity of 100% (95% CI 91.2–100) and a relatively low specificity of 51.1% (95% CI 35.8–66.3). During a post-study experiment, a different patient-level classification method was tried. Specifically, a threshold based on the number of parasite candidates was used to determine whether a patient was infected or uninfected. As a result, Malaria Screener achieved 91.8% (95% CI 83.8–96.6) accuracy, 92.5% (95% CI 79.6–98.4) sensitivity, and 91.1% (95% CI 78.8–97.5) specificity. This result meets the WHO Level 1 criterion in the parasite detection category.

**Table 3:** Malaria Screener and PVF-Net evaluation using PCR as a reference

Evaluation Metrics	Accuracy, % (95% CI)	Sensitivity, % (95% CI)	Specificity, % (95% CI)
Index tests			
Malaria Screener (85 patients)	71.8 (61.0–81.0)	89.6 (77.3–96.5)	48.7 (31.9–65.6)
PVF-Net (189 patients)	81.0 (74.6–86.3)	81.1 (72.6–87.9)	80.8 (70.3–88.8)

#### Parasite detection with PVF-Net—post-study experiment

The images of Sudan data were re-analysed during this post-study experiment. Results are listed below (PVF-Net cannot handle mixed infections; therefore, one patient with a mixed infection of *P. falciparum* and *P. vivax* was excluded. Thus, the total number of patients is 189 rather than 190 for this experiment). PVF-Net correctly identified whether there was a malaria infection for 157 of 189 patients through thick smear analysis, yielding an accuracy of 83.1% (95% CI 77.0–88.1). This result meets the WHO Level 2 requirement for parasite detection. The sensitivity is 86.9% (95% CI 78.6–92.8), and the specificity is 78.9% (95% CI 69.0–86.8). For *P. falciparum only*, its accuracy is 82.8% (95% CI 75.8–88.4), sensitivity is 88.5% (95% CI 77.8–95.3), and specificity is 78.9% (95% CI 69.0–86.8). For *P. vivax only*, its accuracy is 80.5% (95% CI 72.5–86.9), sensitivity is 84.2% (95% CI 68.8–94.0), and specificity is 78.9% (95% CI 69.0–86.8).

#### Detection sensitivity at different parasitaemia levels

**Fig. 4:**
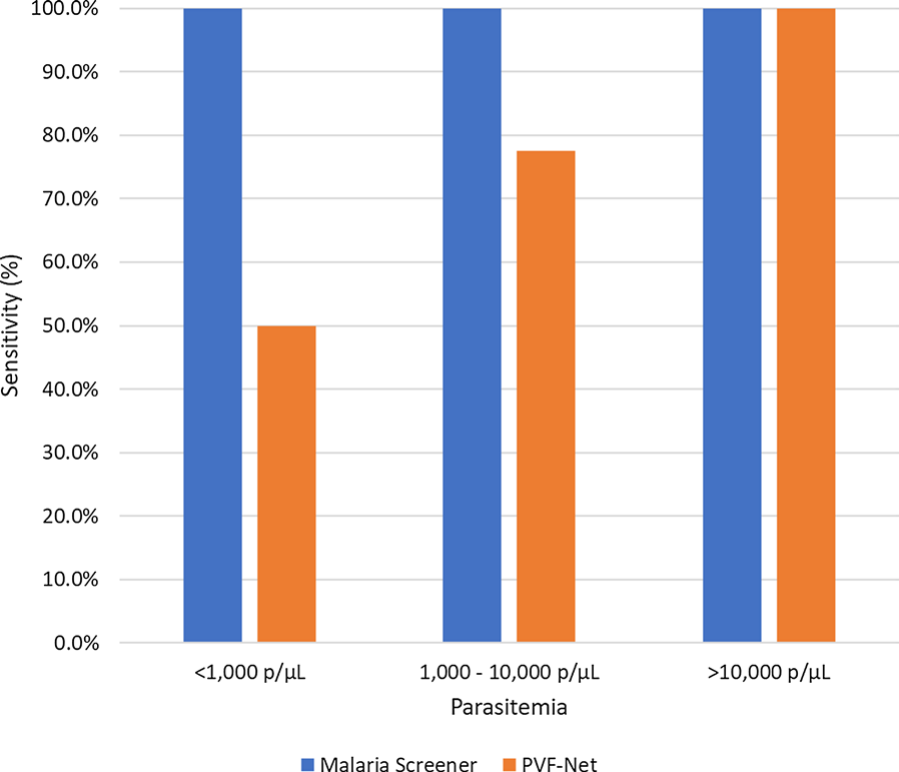
Sensitivity (%) of Malaria Screener and PVF-Net at different parasitaemia levels

The sensitivity of the system was measured at different parasitaemia levels. The samples were separated into three parasite density groups: < 1000 p/µL, 1000 – 10,000 p/µL, and > 10,000 p/µL. Sensitivity maintained the same for Malaria Screener among the three groups. It was 100% (95% CI 2.5-100) at < 1000 p/µL (n = 1), 100% (95% CI 79.4-100) at 1000–10,000 p/µL (n = 16), and 100% (95% CI 85.2-100) at > 10,000 p/µL (n = 23). Sensitivity varied for PVF-Net among the three groups. It was 50.0% (95% CI 15.7-84.3) at < 1000 p/µL (n = 8), 77.5% at (95% CI 61.6-89.2) 1000–10,000 p/µL (n = 40), and 100% (95% CI 93.0-100) at > 10,000 p/µL (n = 51) (Fig. 4).

### Evaluation using nested PCR as a reference

Nested PCR tests were performed on all 190 patients and compared with results from Malaria Screener and PVF-Net. The 85 non-*P. vivax* patients at the SOR site include 40 microscopy-positive patients with *P. falciparum* infection and 45 negative patients. A nested PCR test confirmed microscopy diagnosis for 77 patients while finding parasites in 8 microscopy-negative patients. Malaria Screener only identified three of those eight slides as positive. Thus, compared to PCR, Malaria Screener’s detection accuracy dropped to 71.8% (95% CI 61.0–81.0). The sensitivity is 89.6% (95% CI 77.3–96.5), and the specificity is 48.7% (95% CI 31.9–65.6).

When compared to PCR on 189 patients, PVF-Net correctly detected whether malaria was present in 153 of 189 patients, reaching 81.0% (95% CI 74.6–86.3) accuracy. It has a relatively high sensitivity of 81.1% (95% CI 72.6–87.9) while achieving a relatively high specificity of 80.8% (95% CI 70.3–88.8). More details are shown in Table 3.

### Processing time

Following the semi-automated approach, the app analyses each image automatically, while the user identifies FoVs. Hence, the total time needed to process one smear contains both the app’s runtime and the user’s operating time. It took, on average, only 11.47 and 9.96 s for the app to analyse one thin and thick smear image, respectively, on the used smartphone devices. However, since users also needed time to adjust the microscope between FoVs, they found that the overall processing time per smear for Malaria Screener was only slightly shorter than manual microscopy, although Malaria Screener is much faster in processing each FoV. However, the user’s operating time was not assessed systematically in this study. The above statement is only based on users’ observations.

### Inter-observer variation among microscopists

**Fig. 5:**
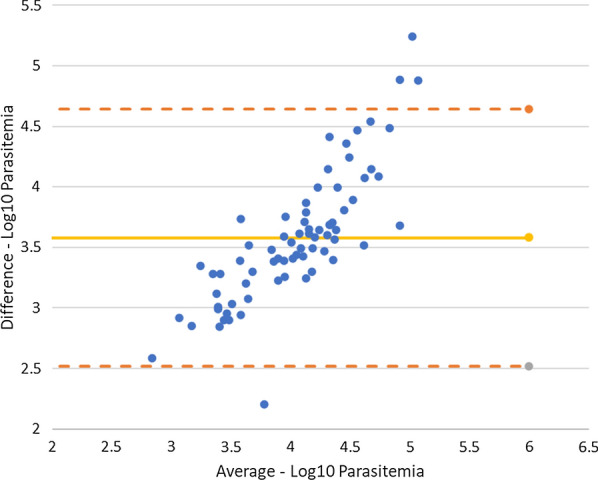
Bland–Altman plot for parasitaemia estimations between the first two microscopist readings

A cross-checking quality control system was implemented during the reference microscopy test. Among 100 patients with positive reads, the first two microscopist readings reached consensus decisions regarding species and parasitaemia for only 27 patients while having discordant diagnoses for 73 patients, according to the Obare method calculator. A Bland–Altman plot (Fig. 5) for assessing agreement of parasitaemia estimations between the first two microscopist readings showed the mean difference to be 3.58, and limits of agreement range from 2.52 to 4.64 on a logarithmic scale.

### Microscopists vs. PCR

Nested PCR tests confirmed microscopy diagnosis for 178 patients while finding parasites in 11 patients that microscopists identified as negative. Thus, compared to PCR, the WHO Level 1 microscopists achieved a 94.2% accuracy, confirming that they meet the WHO Level 1 requirement for parasite detection.

## Discussion

Malaria Screener screened 85 patients from the SOR site for *P. falciparum*. It achieved 74.1% (95% CI 63.5–83.0) accuracy, 100% (95% CI 91.2–100) sensitivity, and 51.1% (95% CI 35.8–66.3) specificity. Switching the patient-level classification method improved the results to 91.8% (95% CI 83.8–96.6) accuracy, 92.5% (95% CI 79.6–98.4) sensitivity, and 91.1% (95% CI 78.8–97.5) specificity. PVF-Net screened 189 patients from both sites for *P. falciparum* and *P. vivax*. It achieved 83.1% (95% CI 77.0–88.1) accuracy, 86.9% (95% CI 78.6–92.8) sensitivity, and 78.9% (95% CI 69.0–86.8) specificity. PVF-Net demonstrated a more robust performance than Malaria Screener, especially in specificity and its ability to detect both *P. falciparum* and *P. vivax.*

Overall, although the results from the two systems are still inferior to the top-tier expert microscopists and the systems were not ready yet for species identification and parasite counting, their performance showed that automated systems have the potential to be used in real-world settings. In addition, the results showed that the developed machine learning algorithms are generalizable in that this field study was conducted in a different malaria-endemic region (Sudan) and by another group of malaria experts, compared to the region in which the original training data was acquired and where the system was trained (Bangladesh, Thailand).

### Patient-level result

This study reported patient-level results, an essential feature of any computer-aided system for malaria diagnosis to be meaningful in the field. However, such field studies have been lacking in smartphone-assisted malaria microscopy. Most studies were only focusing on object-level or image-level evaluations so far. Even outside the scope of smartphone-based systems, only one such patient-level study [6] was found.

There are some limitations to this study. The test data was not collected so that it could be easily organized to evaluate the software’s performance in diagnosing patients with different levels of parasitaemia. Although such an evaluation was included in this paper, the small number of patients at low parasitaemia levels is less than ideal. Besides, the study was not designed to evaluate the software’s performance for different slide qualities.

### High sensitivity and low specificity

Malaria Screener demonstrated high sensitivity and low specificity performance during the field evaluation. This is primarily due to many parasite-like artifacts in the images. Unlike the training data, the field data contains more parasite-like staining artifacts, which are difficult for the model to distinguish. These artifacts caused false detections that led to negative patients being diagnosed as positive and likely increased the parasite count in positive patients. More details about the system’s performance can be found in the additional files 2, 3, 4, and 5 (File 2 includes overlays of blood smear images with detected parasite candidates. File 3 contains microscopy and PCR results for each patient, and File 4 contains object-level scores. Finally, File 5 provides object score histograms for each patient.). There are several options to improve this aspect in future iterations of the software. For instance, one obvious method is to add more real-world data with parasite-like artifacts to the training set, which provides the model with more examples to learn about such artifacts. Another way is to add a filter after the parasite detection step to reduce the number of false-positive detections. In addition, a threshold for the number of parasite candidates in the post-study experiment helped to overcome this problem, leading to a much higher specificity of 91.1% (95% CI 78.8 – 97.5). This is because the number of parasite candidates per slide is much lower for negative patients.

### From object-level scores to patient-level diagnosis

The results of the post-study experiment showed that the method used for computing patient-level results greatly impacts the system’s patient-level performance. Initially, the image score was generated by calculating the mean of the object scores, and then, the patient score was generated by calculating the mean of the image scores. This method ignored the number of parasite candidates detected for a patient. Using a threshold for the maximum number of parasite candidates allowed in a negative patient avoided this issue and led to a much higher performance. If the number of parasite candidates for a patient exceeded the threshold, the patient was considered positive.

### Using PCR as a reference test

PCR was used as a reference test to see what the performance would be when evaluated with a more sensitive method. In addition, sponsors were interested in evaluating the performance of the microscopists compared to PCR. The results showed that the performance of Malaria Screener and PVF-Net dropped when compared to PCR, especially in sensitivity, as expected. However, both systems still achieved the same WHO competency levels. The microscopists achieved an accuracy of 94%, confirming their WHO Level 1 qualification.

### Practicality

For automated malaria diagnostic applications, practicality plays an integral part in whether field practitioners will accept the technology. For this study, the following points are worth highlighting in this respect:

i. Easy to deploy. The app itself can be easily downloaded from Google Play Store. A microscope adapter can be purchased online via a variety of options. Lastly, the app can run on standard Android devices.
ii. Easy to use. A user manual was provided along with the software. Then, field experts learned how to use it after one short online training session.
iii. Affordable. The testing device, a Samsung Galaxy A10, cost around $150 at retail price. An adapter can cost from $10 to $100.
iv. The experts found that the app used in a semi-automated fashion is not necessarily time-saving, especially when WBC concentration is low and more images must be captured to adhere to the protocol.

## Conclusion

This paper reported evaluation results for Malaria Screener in a field study conducted by clinical experts in Sudan. According to WHO standards, Malaria Screener reached the Level 3 competence requirement in the category of parasite detection, although only for *P. falciparum*. This was improved to Level 1 in a post-study experiment. Also, in a post-study experiment, a deep learning network (PVF-Net) achieved Level 2 competence in the category of parasite detection for both *P. falciparum* and *P. vivax*. To the best of our knowledge, this is the first patient-level evaluation study of a smartphone-based malaria diagnostic application. Therefore, this study can serve as a reference for evaluating similar systems in the future. The application shows promise for malaria screening in resource-limited areas. With continued improvements, especially for species identification and parasitaemia quantitation, Malaria Screener has the potential to facilitate malaria screening in the field.

## Supplementary Information

**Additional file 1:** Malaria_Screener_User_Manual.pdf: User manual for Malaria Screener.

**Additional file 2:** Overlay_images.zip: Example images showing overlays of blood smear images with detected parasite candidates.

**Additional file 3:** Patient_level_results_SOR.xlsx: A file with microscopy results and PCR results for each patient.

**Additional file 4:** Object_scores_SOR.txt: A txt file containing all object scores with corresponding identifiers.

**Additional file 5:** Object_score_histograms.zip: Object score histograms for each patient.

## Data Availability

The dataset collected and analysed for this study is publicly available here: https://data.lhncbc.nlm.nih.gov/public/Malaria/MalariaScreener/index.html.

## Abbreviations

DBS: Dried blood spots
FoV: Field of view
FIND: Global alliance for diagnostics
GS: Gezira Slanj
IEND: Institute of Endemic Diseases, University of Khartoum, Sudan
NLM: National Library of Medicine
NIH: National Institutes of Health
PCR: Polymerase chain reaction
PVF-Net: PlasmodiumVF-Net
RBC: Red blood cell
SOR: Alsororab
WHO: World Health Organization
WBC: White blood cell
