# Supplementary material for: Patient-level performance evaluation of a smartphone-based malaria diagnostic application
Source: Malar J. 2023 Jan 27;22:33. doi: 10.1186/s12936-023-04446-0 (PMC9883923; doi:10.1186/s12936-023-04446-0)
Supplement: Supplementary file 1 — Additional file 1: Malaria_Screener_User_Manual.pdf: User manual for Malaria Screener. [file 12936_2023_4446_MOESM1_ESM.pdf]

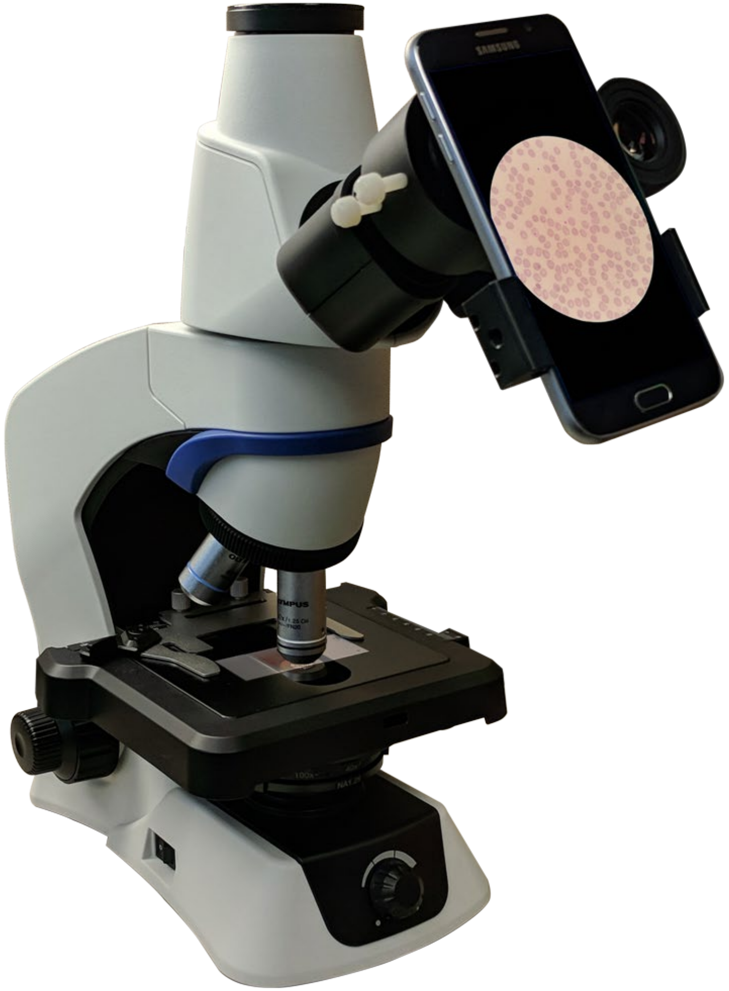

NLM Malaria Screener
User Guideline

Outline

- Steps for Collaboration ………………………………………………………………………………… Page 3
- Malaria Screener User Manual ………………………………………………………………. Page 4
- Frequently Asked Questions ...……………………………………………………………… Page 17

Steps for Collaboration

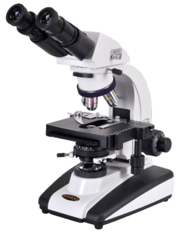

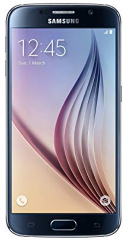

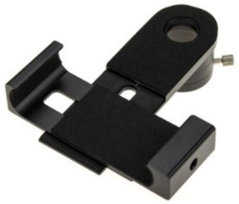

1. Get equipment

- Microscope with 100X objective lens
- Android smartphone with 12+ Mega Pixel camera.
- Adapter

Recommendation: https://www.telescopeadapters.com/128-universal-mounts

* Refer to Question 1 in Frequently Asked Questions for more details on equipment

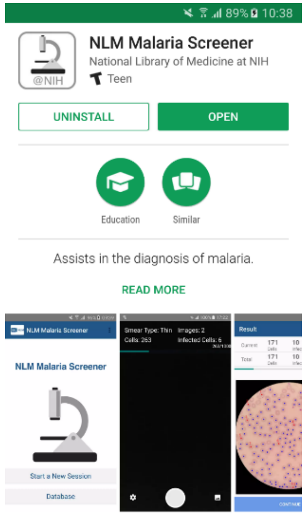

2. Email us

- Email us a Gmail address to join the beta test group

hang.yu@nih.gov

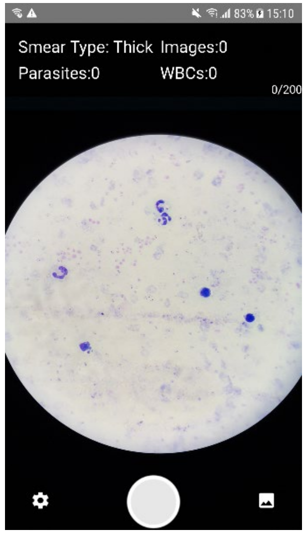

3. Take images

- Uses the app to take images of 10+ thin or thick smears with manual counts.

*No personal data will be collected

Malaria Screener
User Manual

Proper Smear Imaging

To achieve optimal results, proper images should be presented to the app.

- Center Position, field-of-view should fit to image

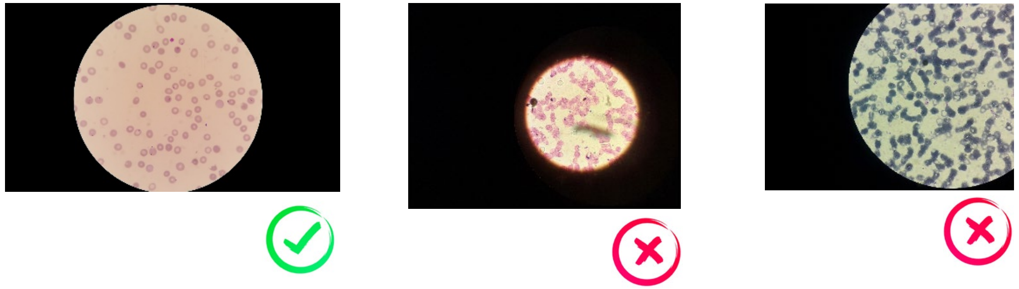

- Even illumination, avoid shadow (mostly along with border)

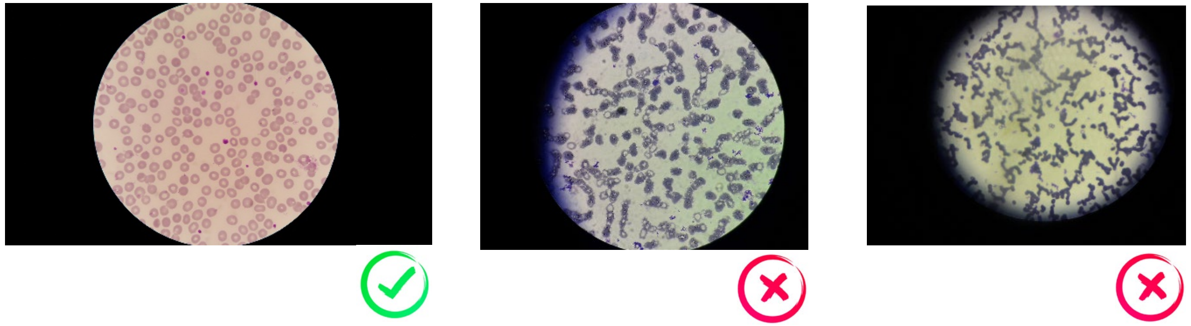

- Clean lens

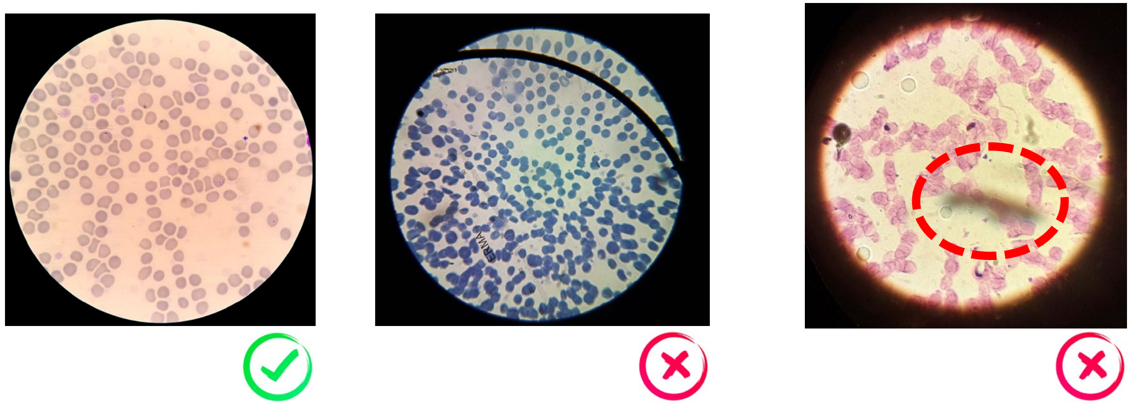

Proper Smear Imaging

- Cells distribution

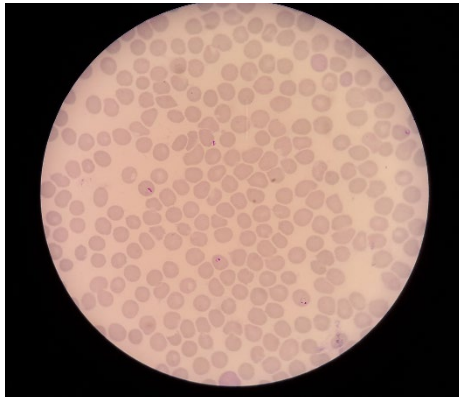

Well separated single cells if possible

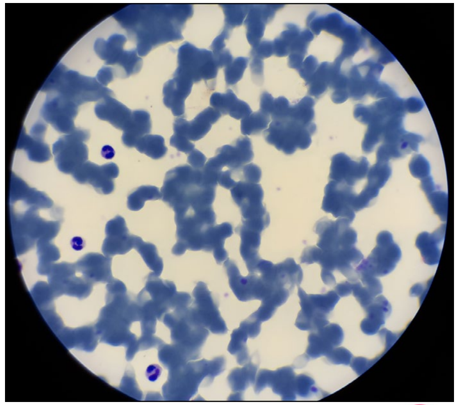

Too dense

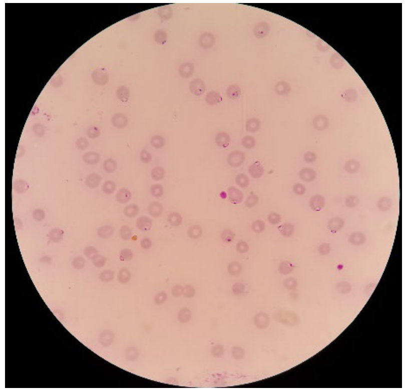

Too sparse

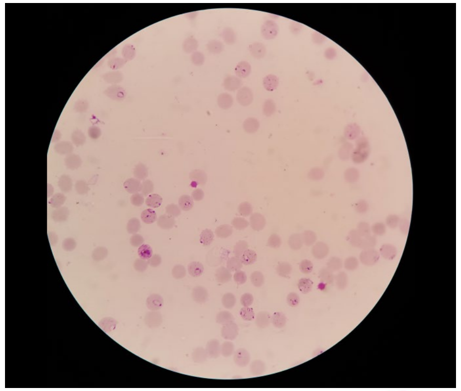

Too sparse

- Sharp image, no blur

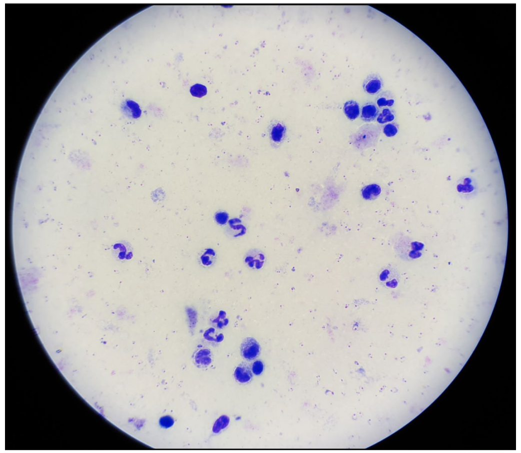

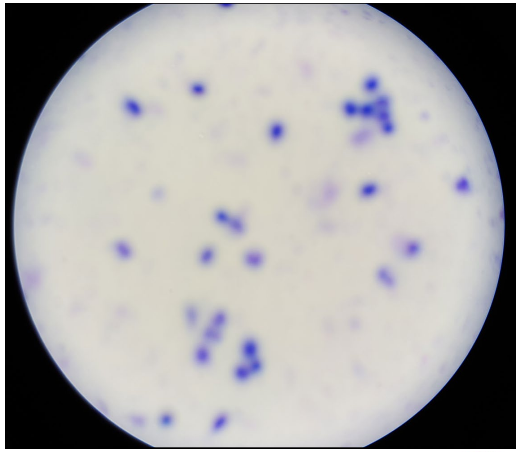

Blood film diagnosis

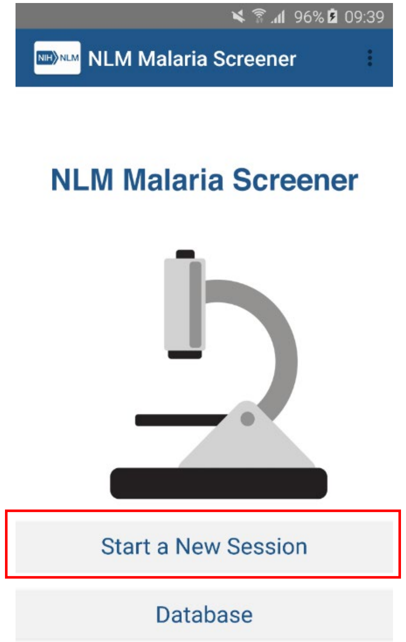

① On the main page of the app, press “Start a New Session”.

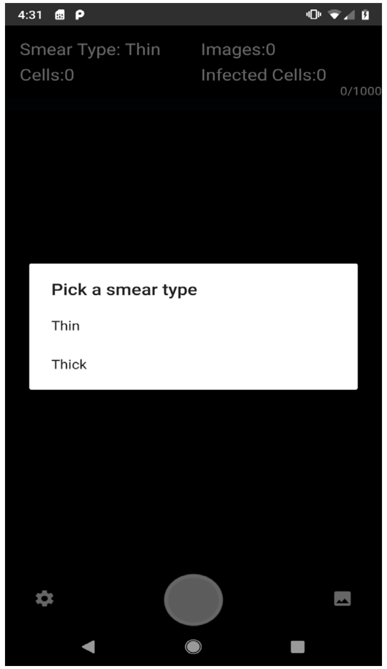

② Select smear type.

Blood film diagnosis

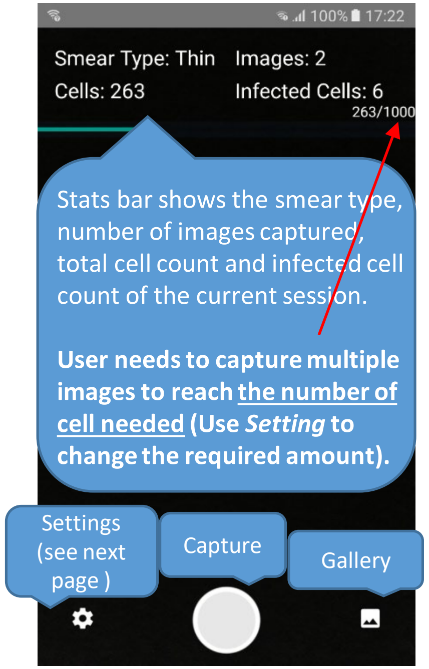

③ Capture an image of the blood film.

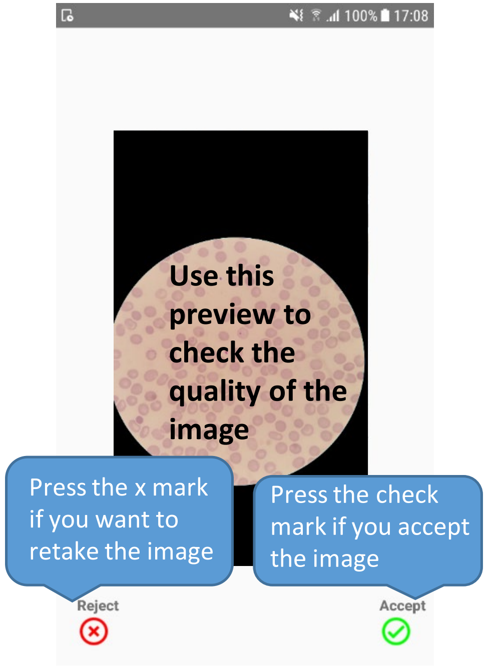

④ Preview the image.

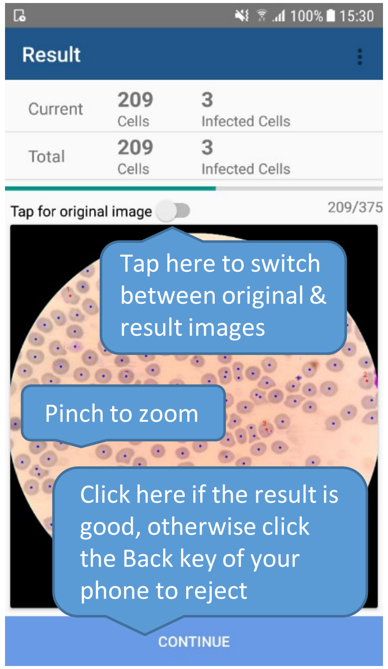

⑤ After the image has been processed, check the result.

Here, thin smear is chosen as an example to show the workflow of a session.

During each session, these three steps are repeated until a certain amount of cells are collected.

Blood film diagnosis

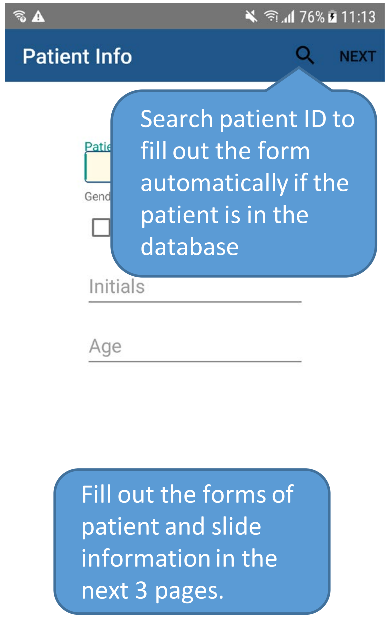

⑥ Input patient information.

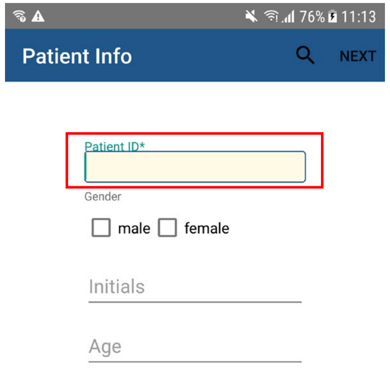

⑦ Fields with asterisk marks are required fields, other fields can be left empty. (Only Patient ID and Slide ID are required fields)

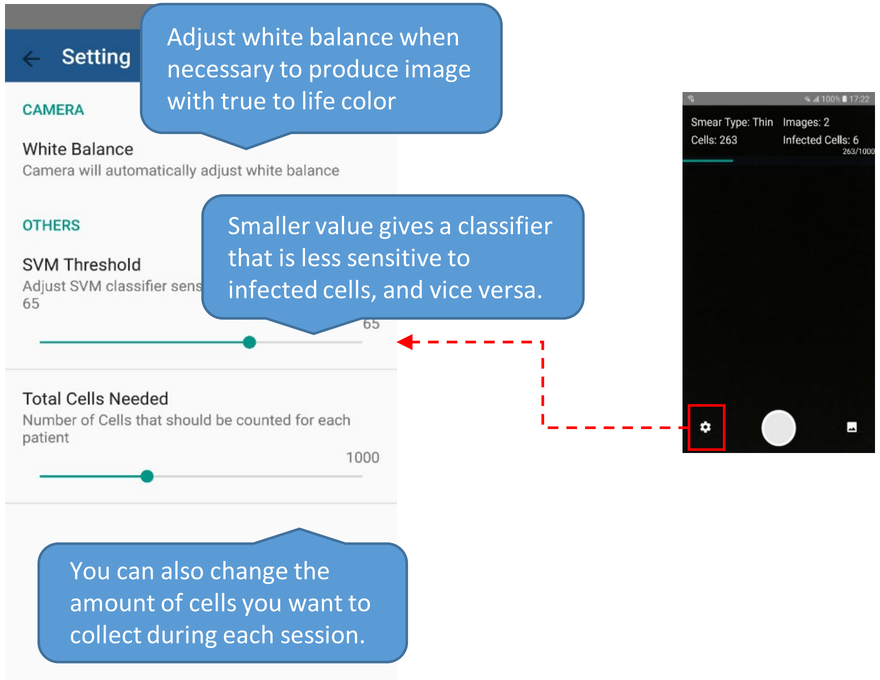

Settings

Database

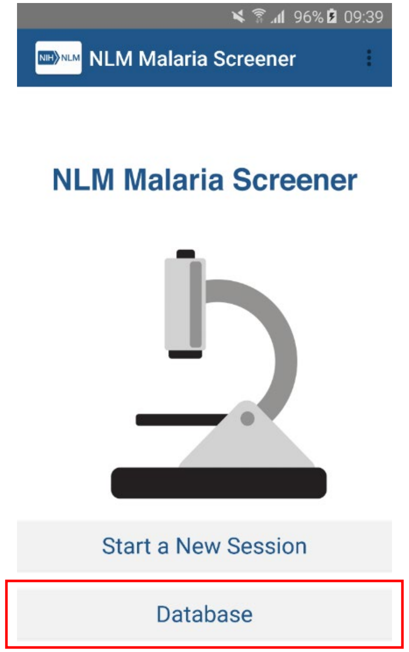

① On the main page of the app, press “Database”.

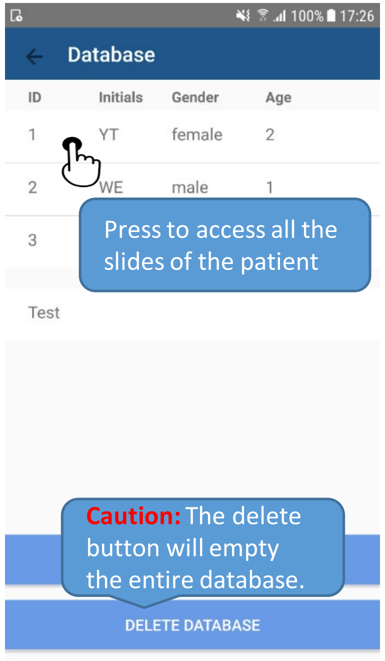

② Data for all the patients are saved in the database.

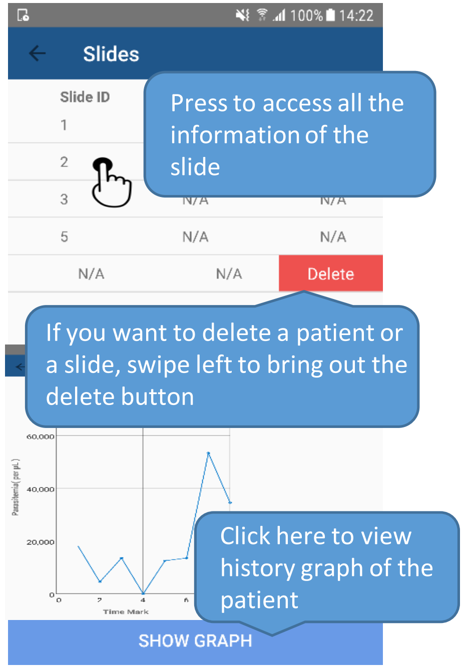

③ Slides for each patient

Database

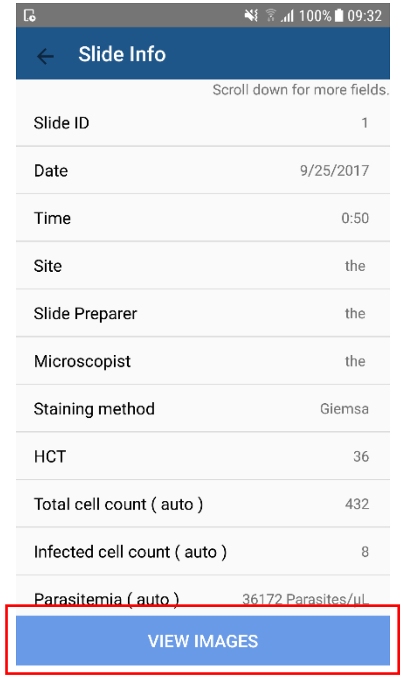

④ Click “VIEW IMAGES” to view all the images of the slide.

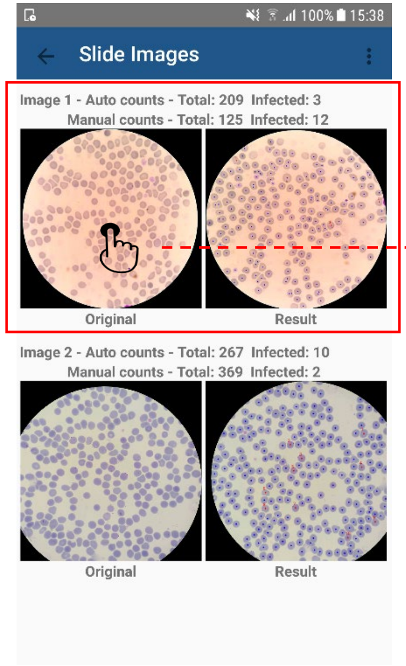

Tap the image to view it in full screen

⑤ Each section displays the original and result images side.

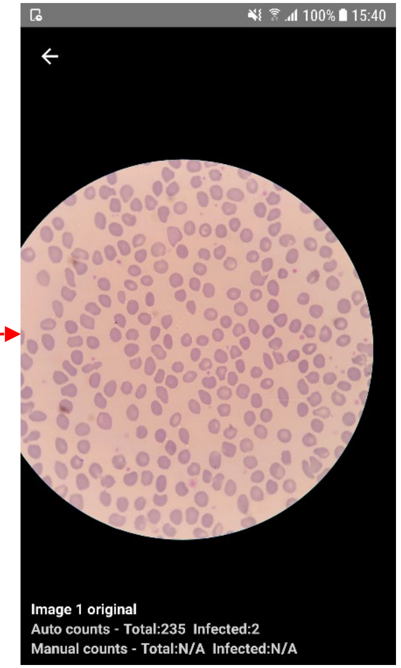

⑥ View image in full screen

Enter manual counts during a session

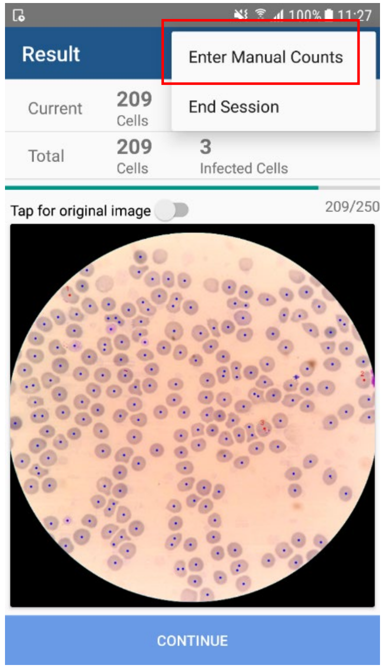

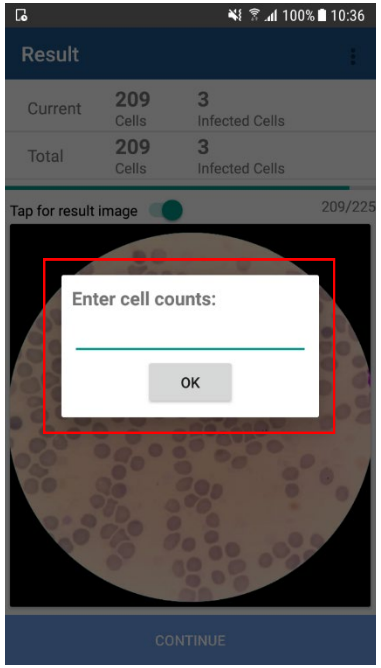

1. Find the more options icon on the top right corner of the result page and press “Enter Manual Counts”.

2. Enter your manual counts in the dialog that appears in the center of the page

Enter manual counts in the database

Ideally, manual counts should be entered during the screening session as shown in the previous page. This is only an alternative method.

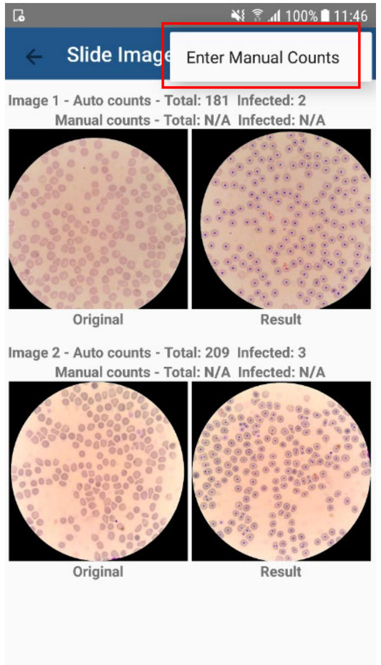

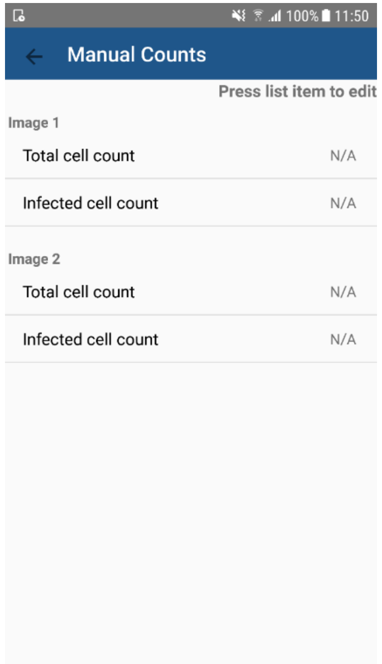

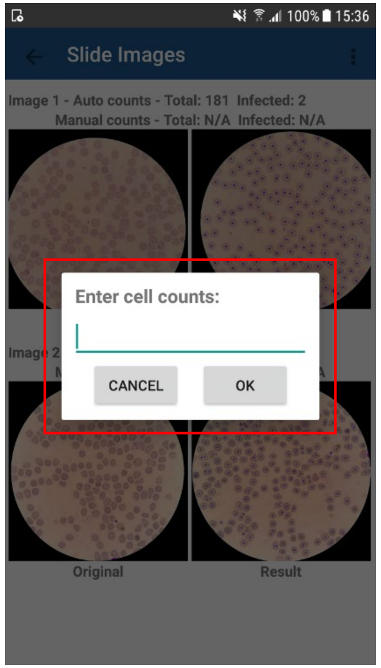

1. Find the more options icon on the top right corner of the "Slide Image" page and press "Enter Manual Counts".
2. The manual counts for each image are listed on this page. Press each list item to edit.
3. Another option is to long press on the image thumbnail, and enter your counts in the dialog that appears.

Upload data

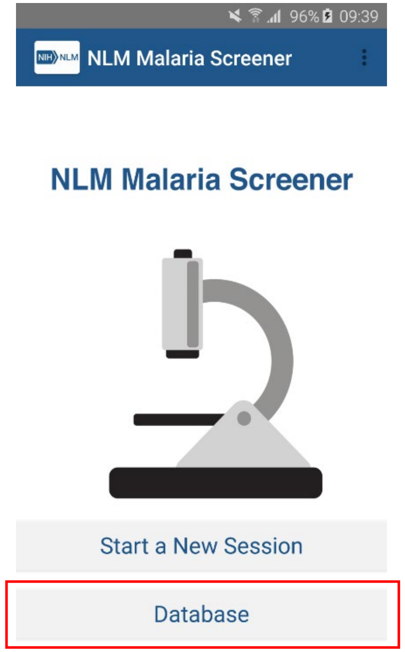

① On the main page of the app, press “database”.

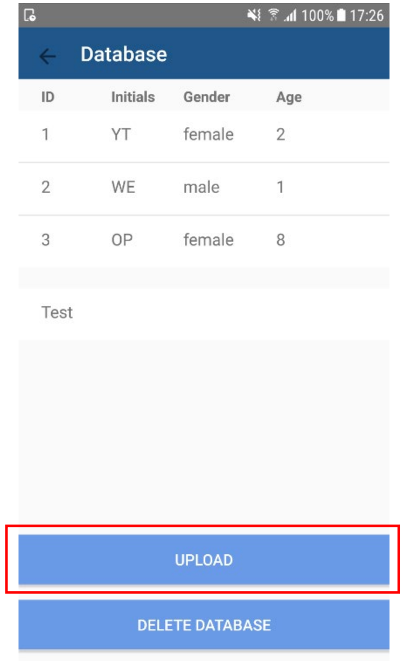

② Press “UPLOAD”.

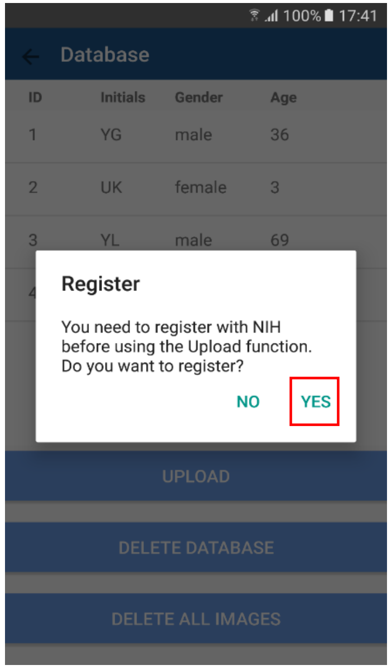

③ Press “YES” and enter registration page.

box

***User needs a Box account in order to use this feature. You can register for a free account at Box.com.**

Upload data

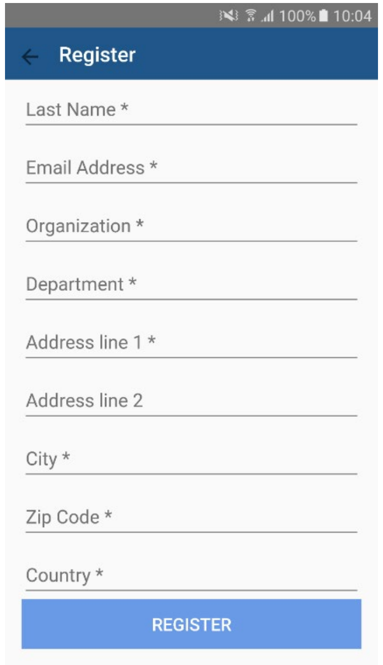

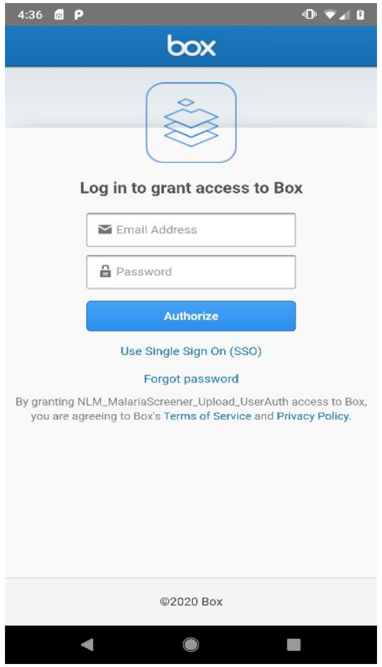

4 Fill out the form and then press “REGISTER”.

5 User will be prompted to enter credentials to grant access to the account.

Upload data

*The app will only perform uploading tasks with a WiFi connection to avoid potential data charges.

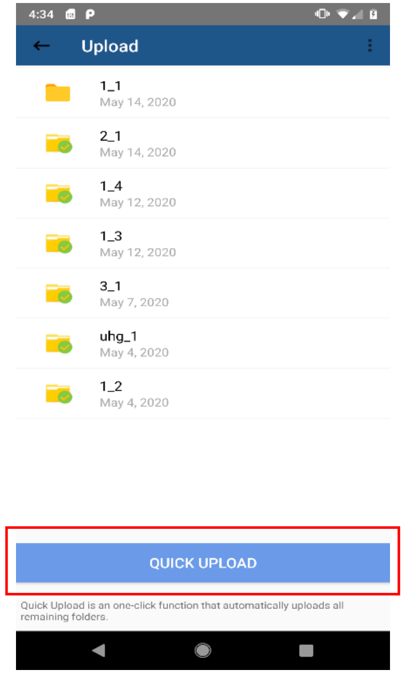

6. **One-click upload**. Simply press the "Quick Upload" button to upload all images that have not been uploaded.

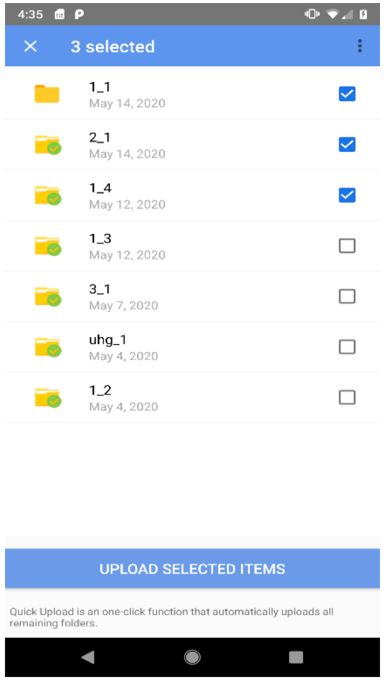

7. **Upload with manual selection**. User can also manually select the folders.

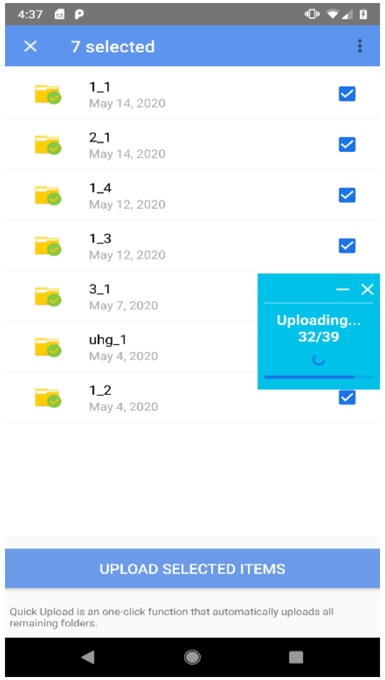

8. Upon uploading, a floating widget indicates the upload progress.

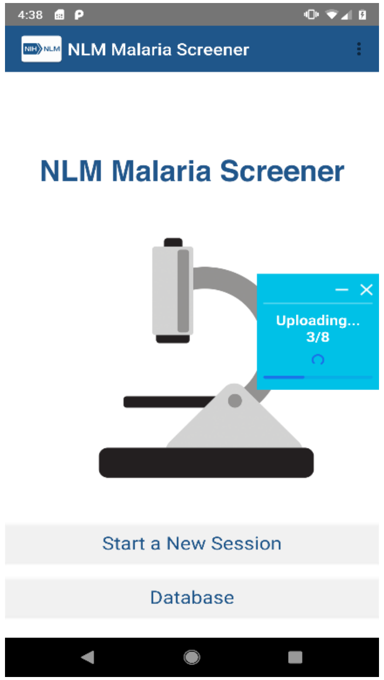

9. **Single Session Upload**. After each session, the app will attempt to upload data from the session. This is to split up the workload.

Image acquisition mode

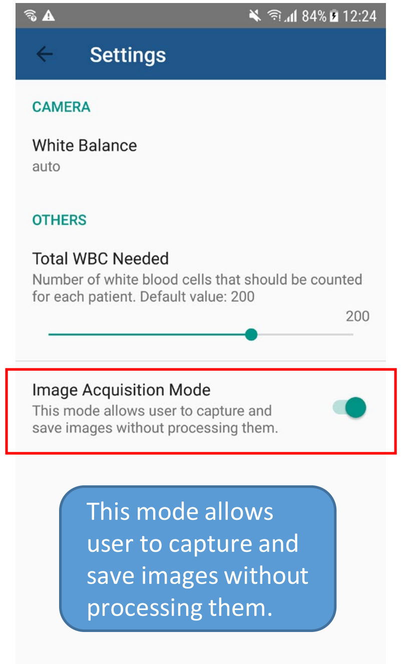

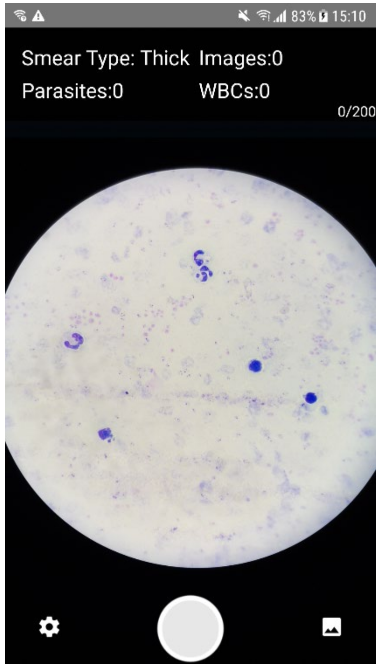

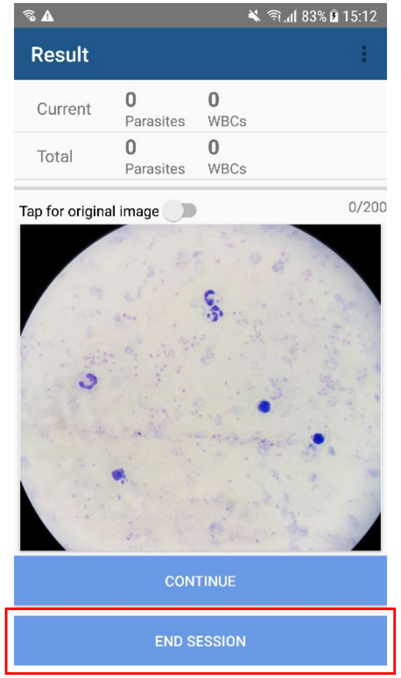

1. At the beginning of a session, turn on Image Acquisition Mode in Settings.
2. Find ideal field and capture images like usual. The image will not be processed, and the app will immediately go to the next page when an image is accepted.
3. The counts will all be zeros since no processing is done in this mode. Press "End Session" when you decide enough images have been captured.

Frequently Asked Questions

1. What smartphone and/or microscope adapter should I order?

Answer: Many smartphones and microscope adapters are good to be used for our app. The key here is for the two to work well with each other, however this is difficult to tell without testing. We give a few tips that will help you when choosing your devices:

A few tips while choosing smartphones & microscope adapters:

- Processor power of the smartphone will determine the processing speed, so a newer model is usually preferred.
- The buttons on the sides of smartphone shall not clash with the clamps of the adapters.
- Smartphones with multi-lens camera are not recommended.
- Go with good quality adapters. A cheap microscope adapter may work. However, it may cause you a lot of trouble.

For example,

   a. It maybe be difficult to get it attached to the microscope.
   b. It maybe be difficult to align the camera with the eyepiece.

Recommendations:

Smartphone: Samsung Galaxy S Series + Microscope adapter: https://www.telescopeadapters.com/128-universal-mounts

Frequently Asked Questions

2. Does the app collect patient’s personal information?

Answer: No, the app does not collect any patient’s personal information.

DEPARTMENT OF HEALTH & HUMAN SERVICES · USA

NIH U.S. National Library of Medicine

LHNCBC

NIH
National Institute of Allergy and Infectious Diseases

wellcome trust
MORU
Tropical Health Network

UNIVERSITY OF OXFORD

M U
