# Supplementary figures and images for: Patient-level performance evaluation of a smartphone-based malaria diagnostic application
Source: Malar J. 2023 Jan 27;22:33. doi: 10.1186/s12936-023-04446-0 (PMC9883923; doi:10.1186/s12936-023-04446-0)

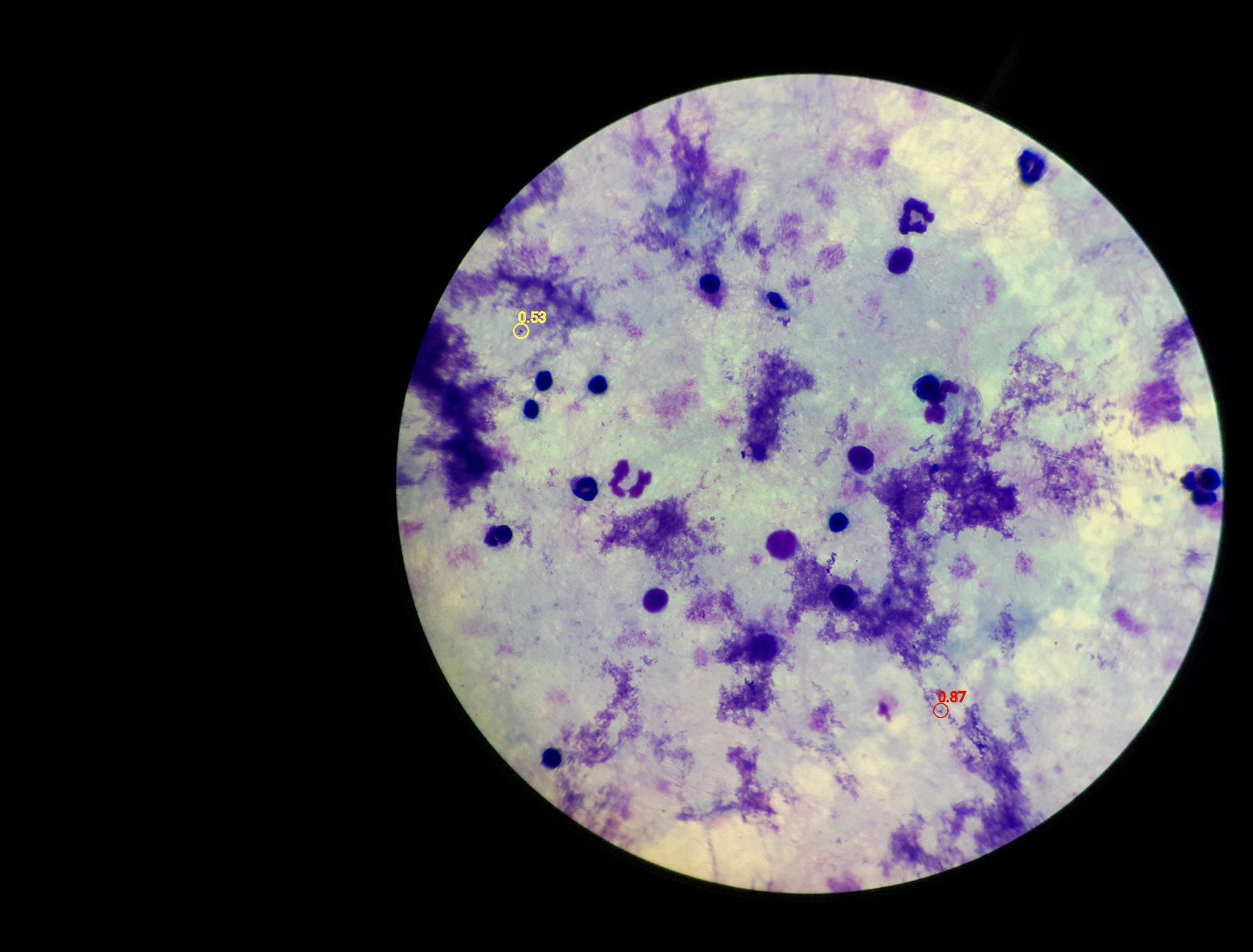

Supplement: Supplementary file 2 — Additional file 2: Overlay_images.zip: Example images showing overlays of blood smear images with detected parasite candidates. [file 12936_2023_4446_MOESM2_ESM.zip › 0002a_1/20210308_083339_Thick_result.jpg]

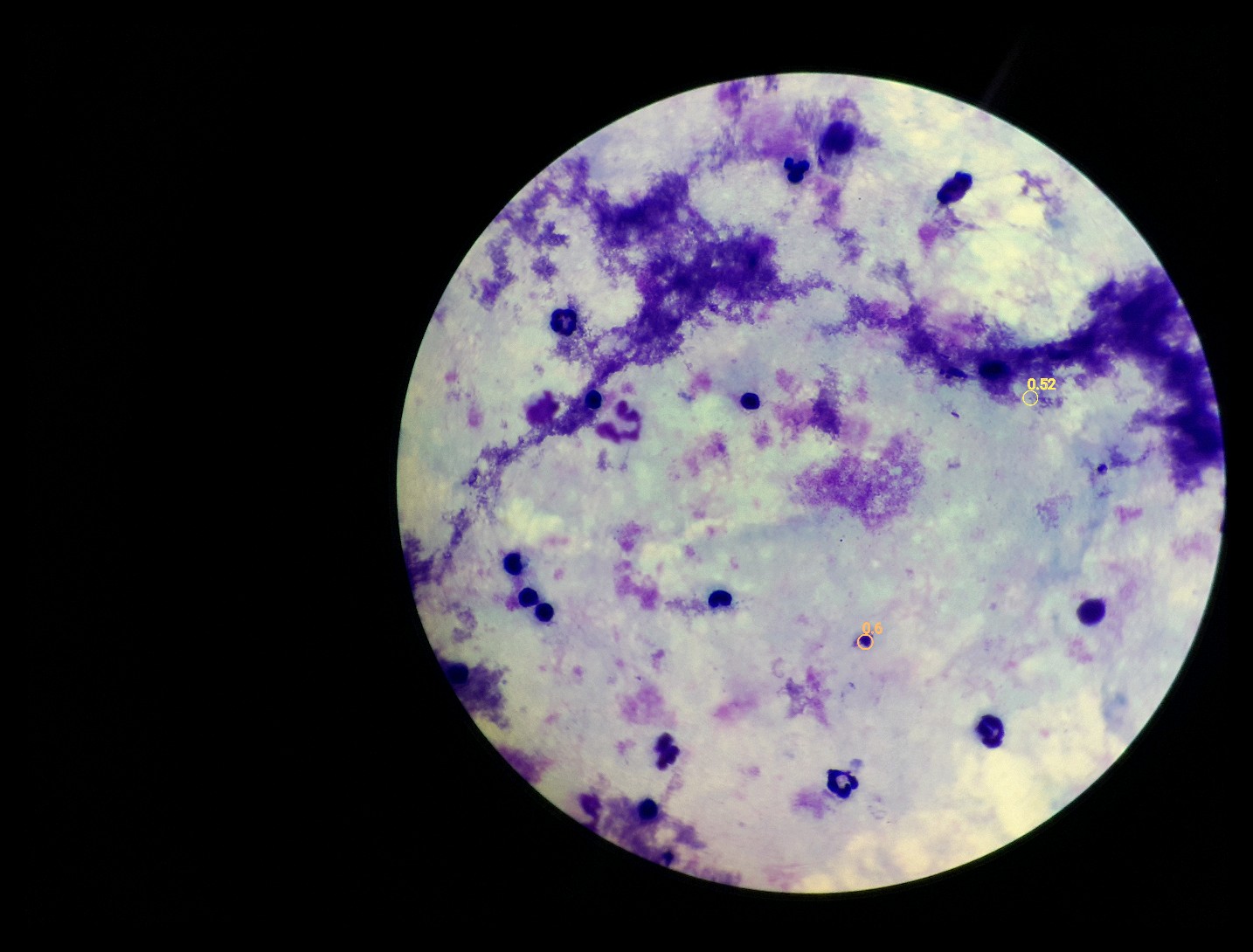

Supplement: Supplementary file 2 — Additional file 2: Overlay_images.zip: Example images showing overlays of blood smear images with detected parasite candidates. [file 12936_2023_4446_MOESM2_ESM.zip › 0002a_1/20210308_083426_Thick_result.jpg]

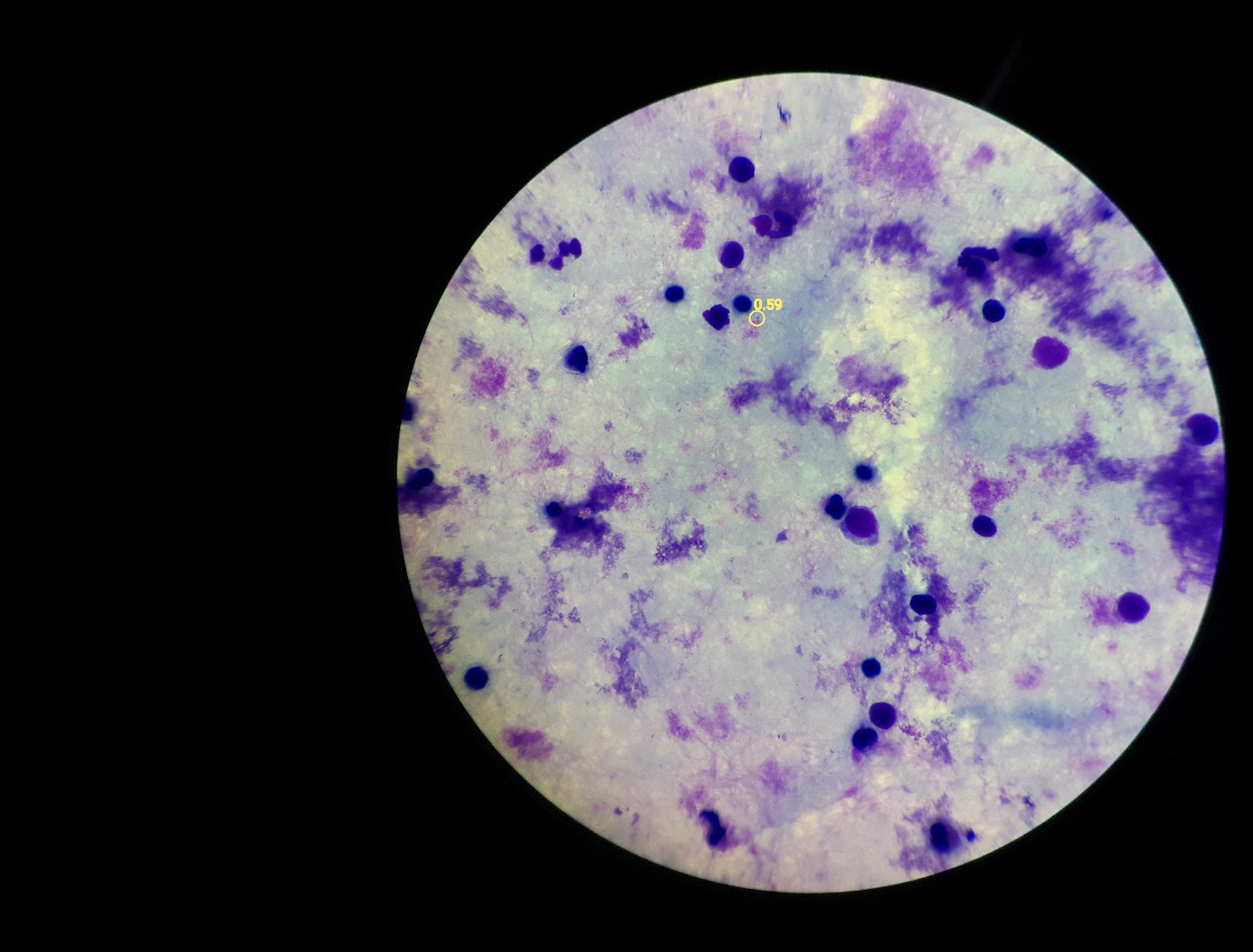

Supplement: Supplementary file 2 — Additional file 2: Overlay_images.zip: Example images showing overlays of blood smear images with detected parasite candidates. [file 12936_2023_4446_MOESM2_ESM.zip › 0002a_1/20210308_083522_Thick_result.jpg]

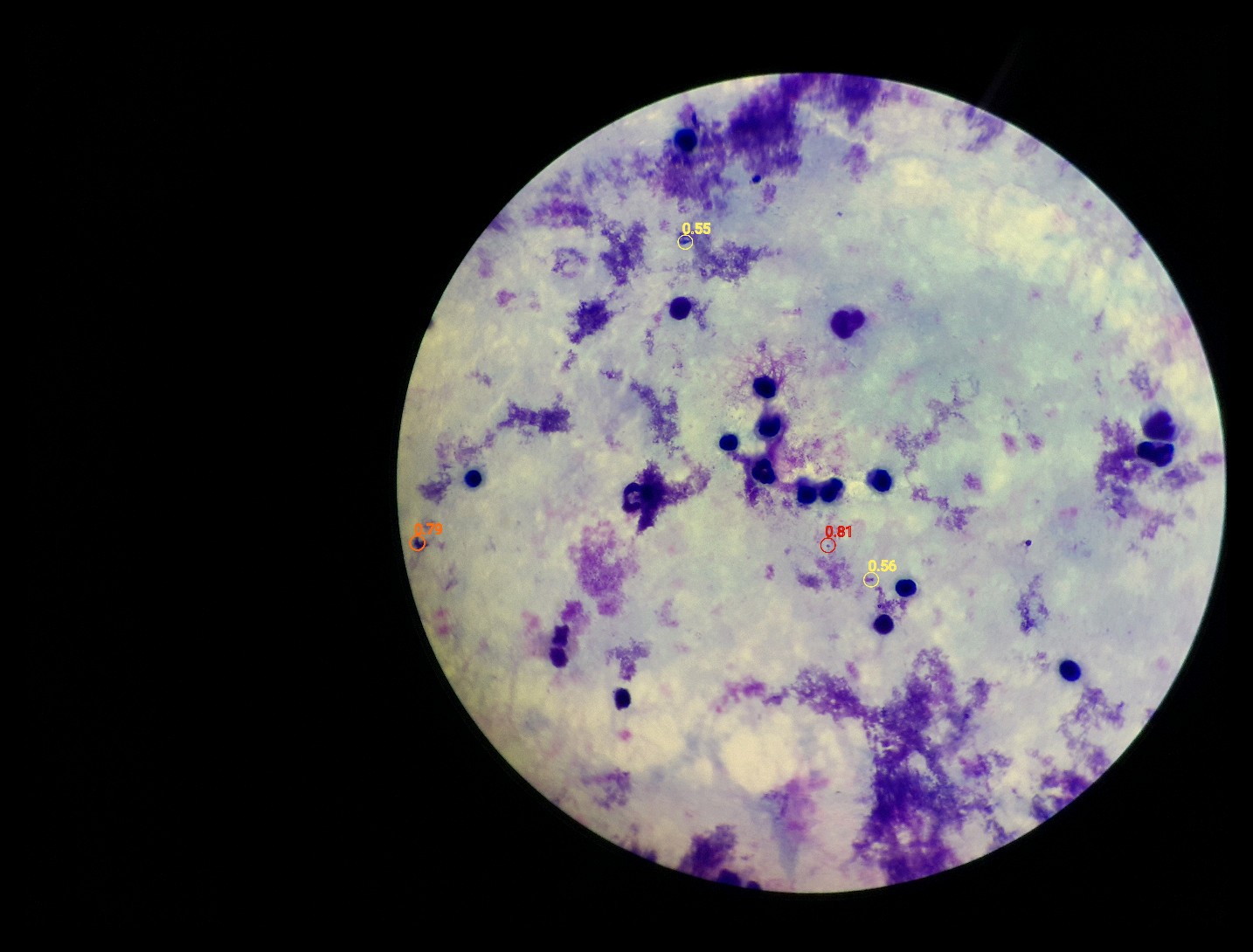

Supplement: Supplementary file 2 — Additional file 2: Overlay_images.zip: Example images showing overlays of blood smear images with detected parasite candidates. [file 12936_2023_4446_MOESM2_ESM.zip › 0002a_1/20210308_083618_Thick_result.jpg]

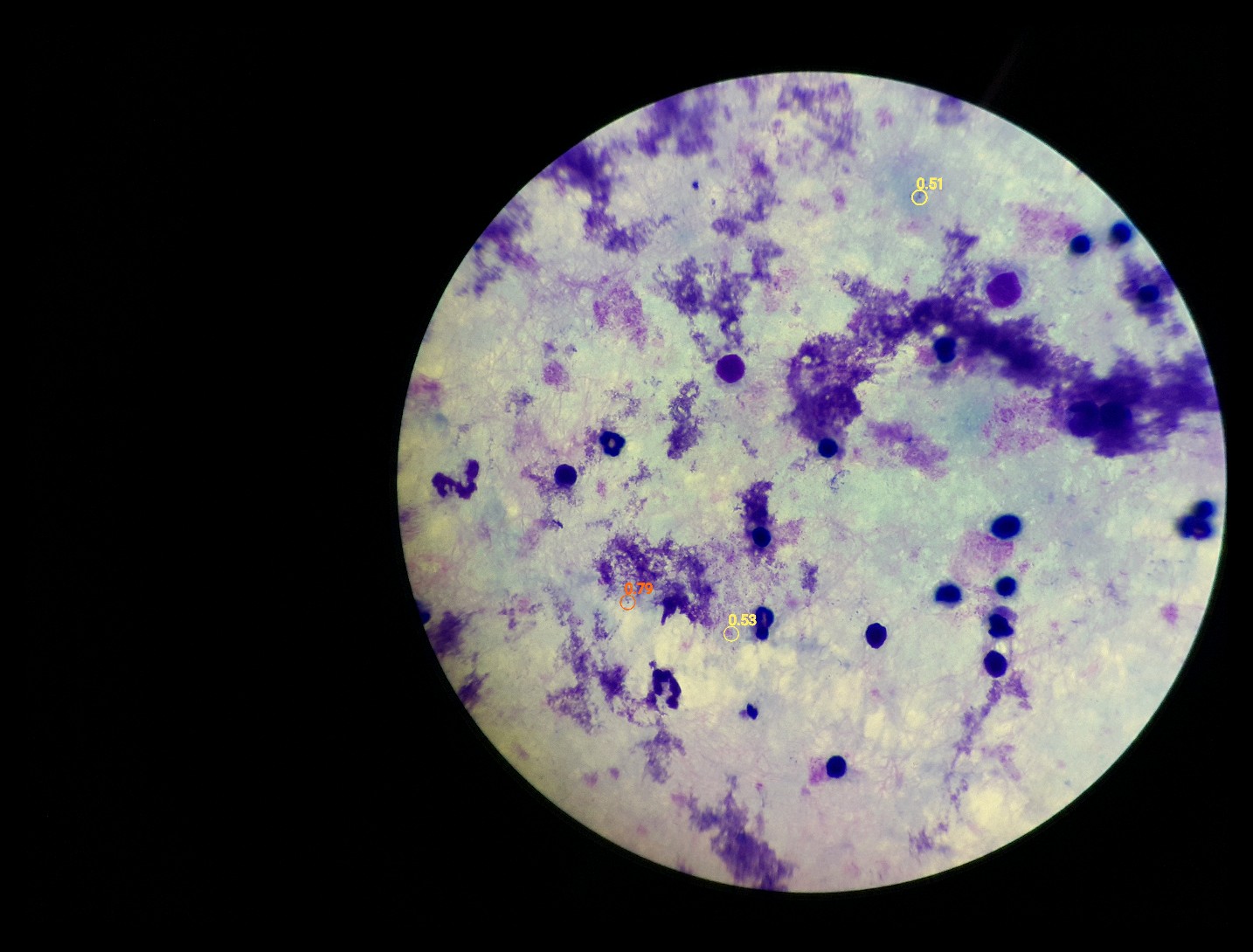

Supplement: Supplementary file 2 — Additional file 2: Overlay_images.zip: Example images showing overlays of blood smear images with detected parasite candidates. [file 12936_2023_4446_MOESM2_ESM.zip › 0002a_1/20210308_083813_Thick_result.jpg]

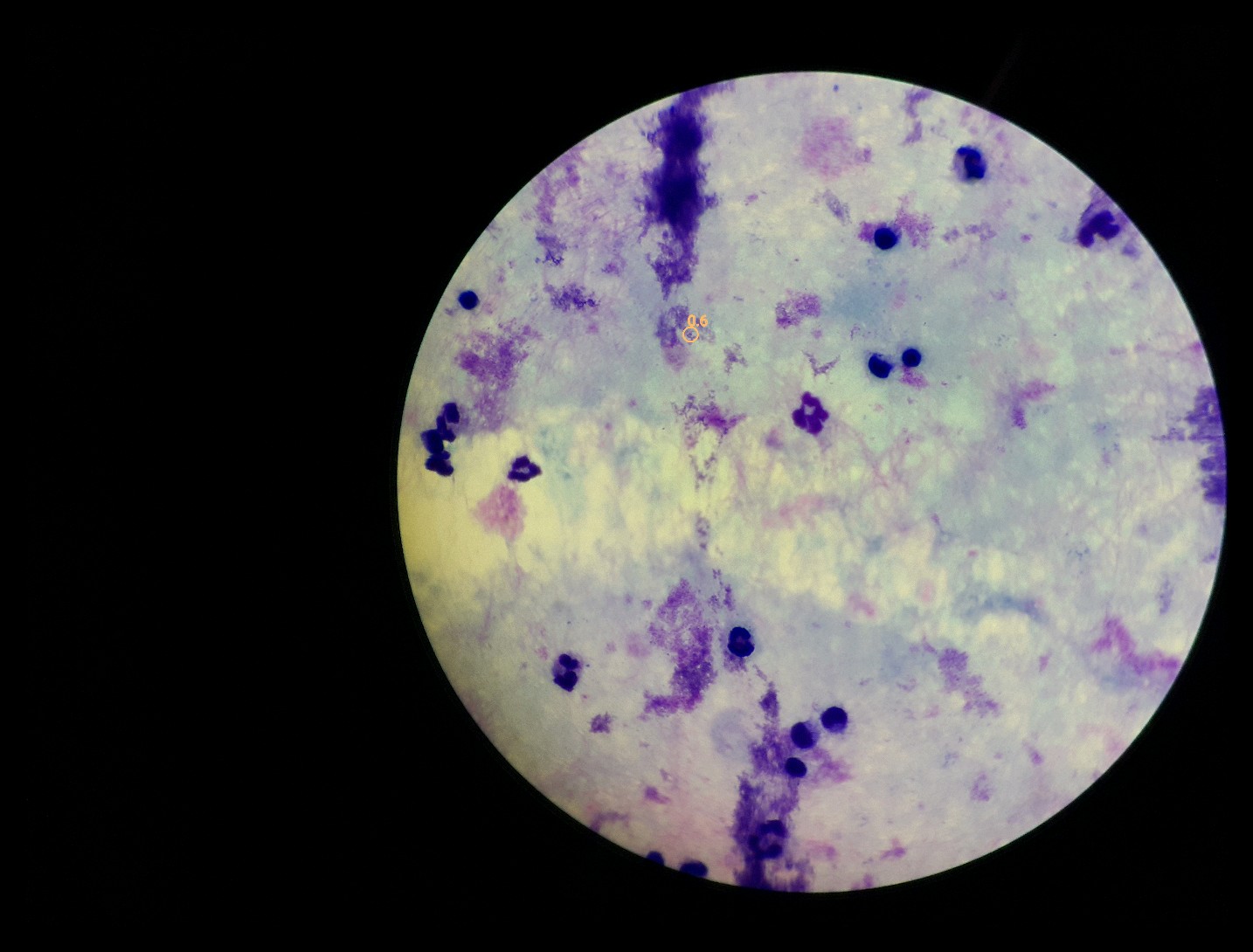

Supplement: Supplementary file 2 — Additional file 2: Overlay_images.zip: Example images showing overlays of blood smear images with detected parasite candidates. [file 12936_2023_4446_MOESM2_ESM.zip › 0002a_1/20210308_083845_Thick_result.jpg]

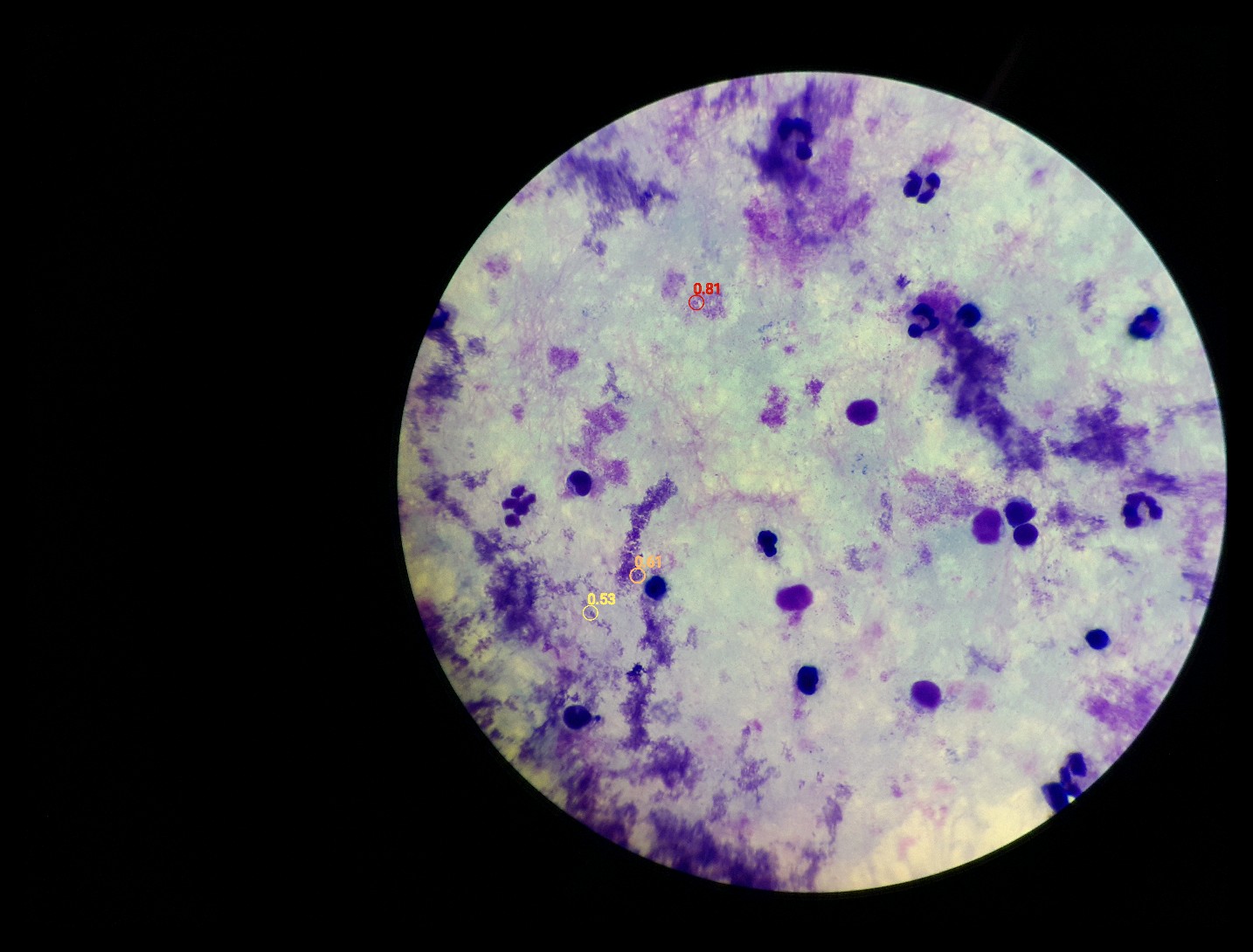

Supplement: Supplementary file 2 — Additional file 2: Overlay_images.zip: Example images showing overlays of blood smear images with detected parasite candidates. [file 12936_2023_4446_MOESM2_ESM.zip › 0002a_1/20210308_083908_Thick_result.jpg]

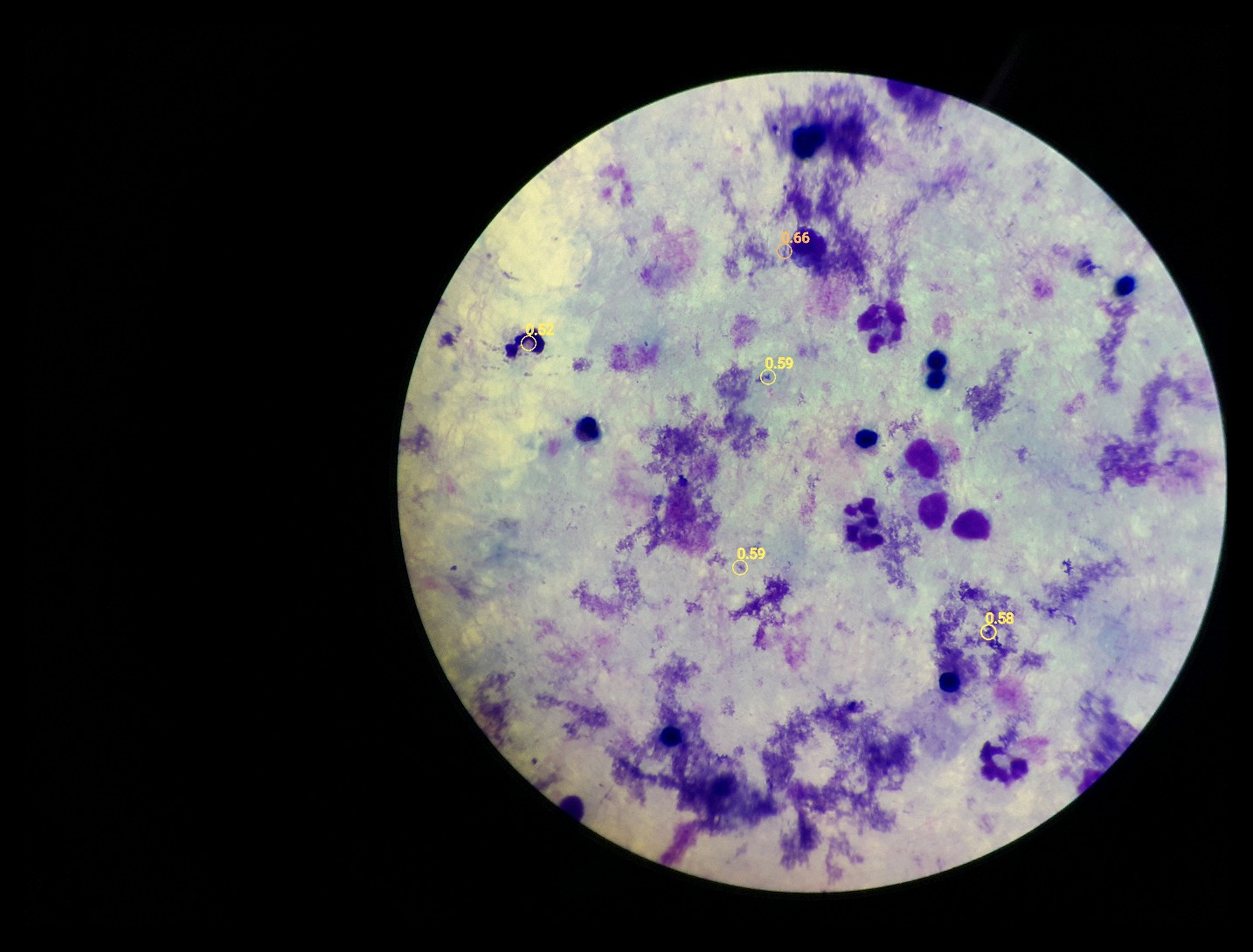

Supplement: Supplementary file 2 — Additional file 2: Overlay_images.zip: Example images showing overlays of blood smear images with detected parasite candidates. [file 12936_2023_4446_MOESM2_ESM.zip › 0002a_1/20210308_083936_Thick_result.jpg]

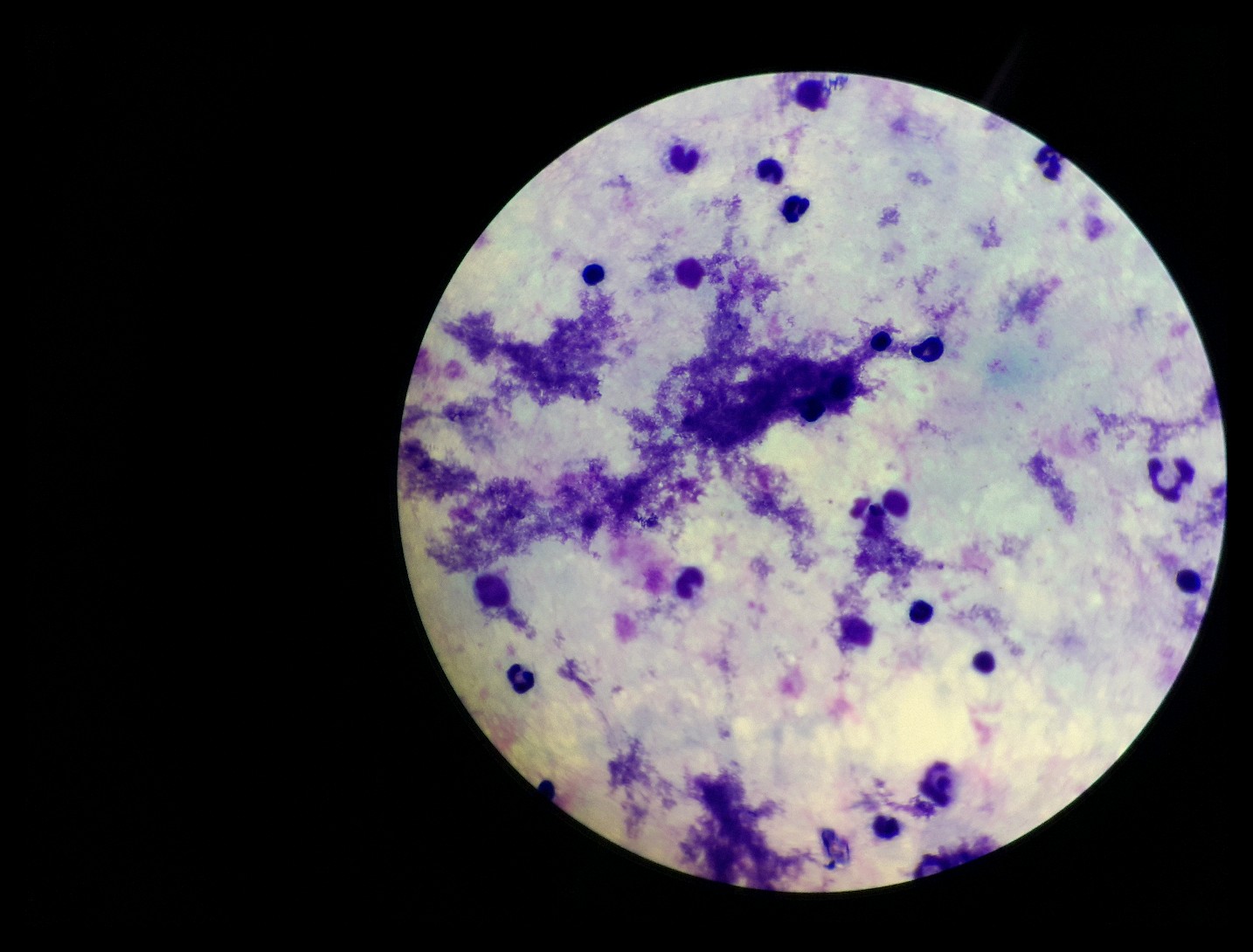

Supplement: Supplementary file 2 — Additional file 2: Overlay_images.zip: Example images showing overlays of blood smear images with detected parasite candidates. [file 12936_2023_4446_MOESM2_ESM.zip › 0002a_1/20210308_084004_Thick_result.jpg]

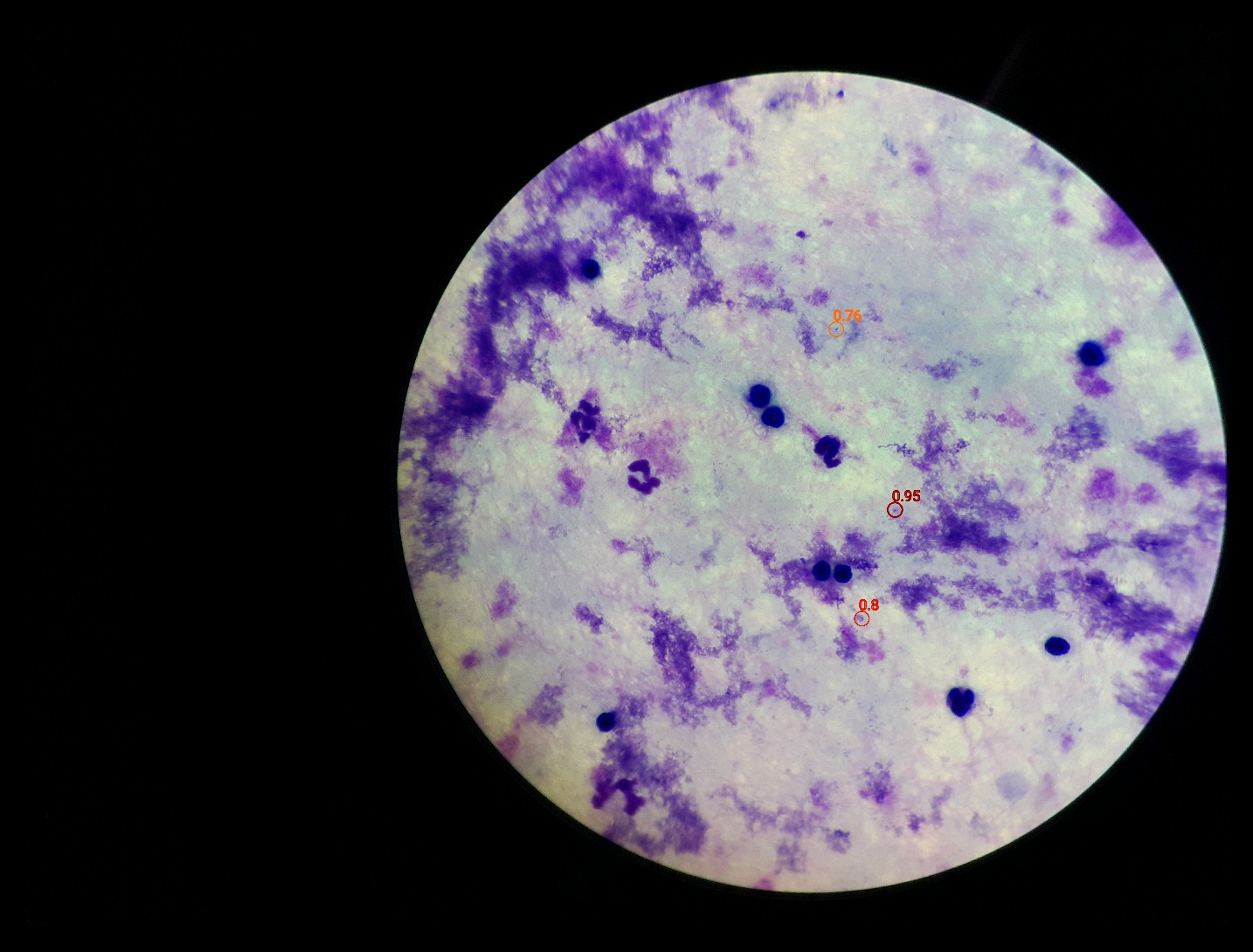

Supplement: Supplementary file 2 — Additional file 2: Overlay_images.zip: Example images showing overlays of blood smear images with detected parasite candidates. [file 12936_2023_4446_MOESM2_ESM.zip › 0002a_1/20210308_084030_Thick_result.jpg]

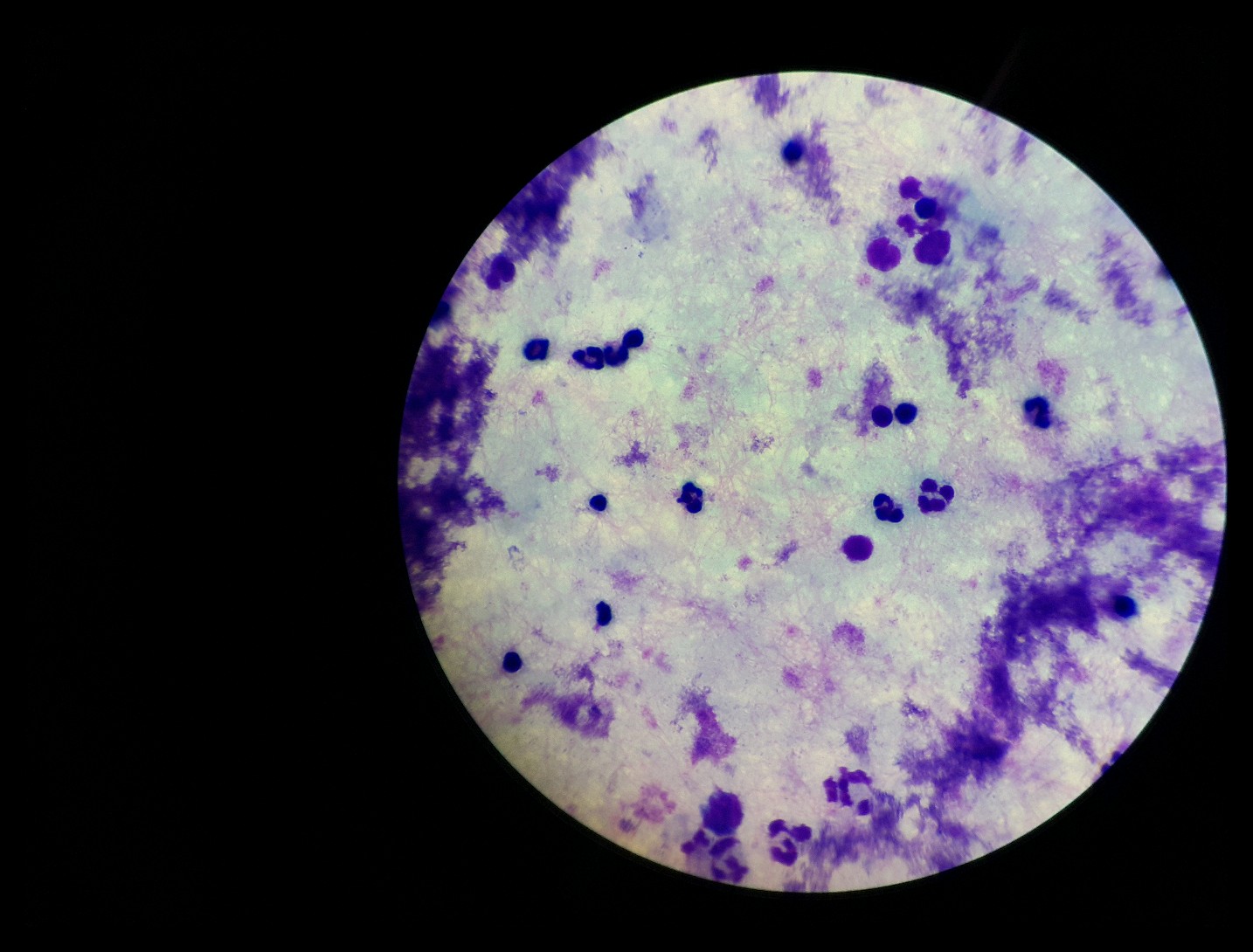

Supplement: Supplementary file 2 — Additional file 2: Overlay_images.zip: Example images showing overlays of blood smear images with detected parasite candidates. [file 12936_2023_4446_MOESM2_ESM.zip › 0002a_1/20210308_084055_Thick_result.jpg]

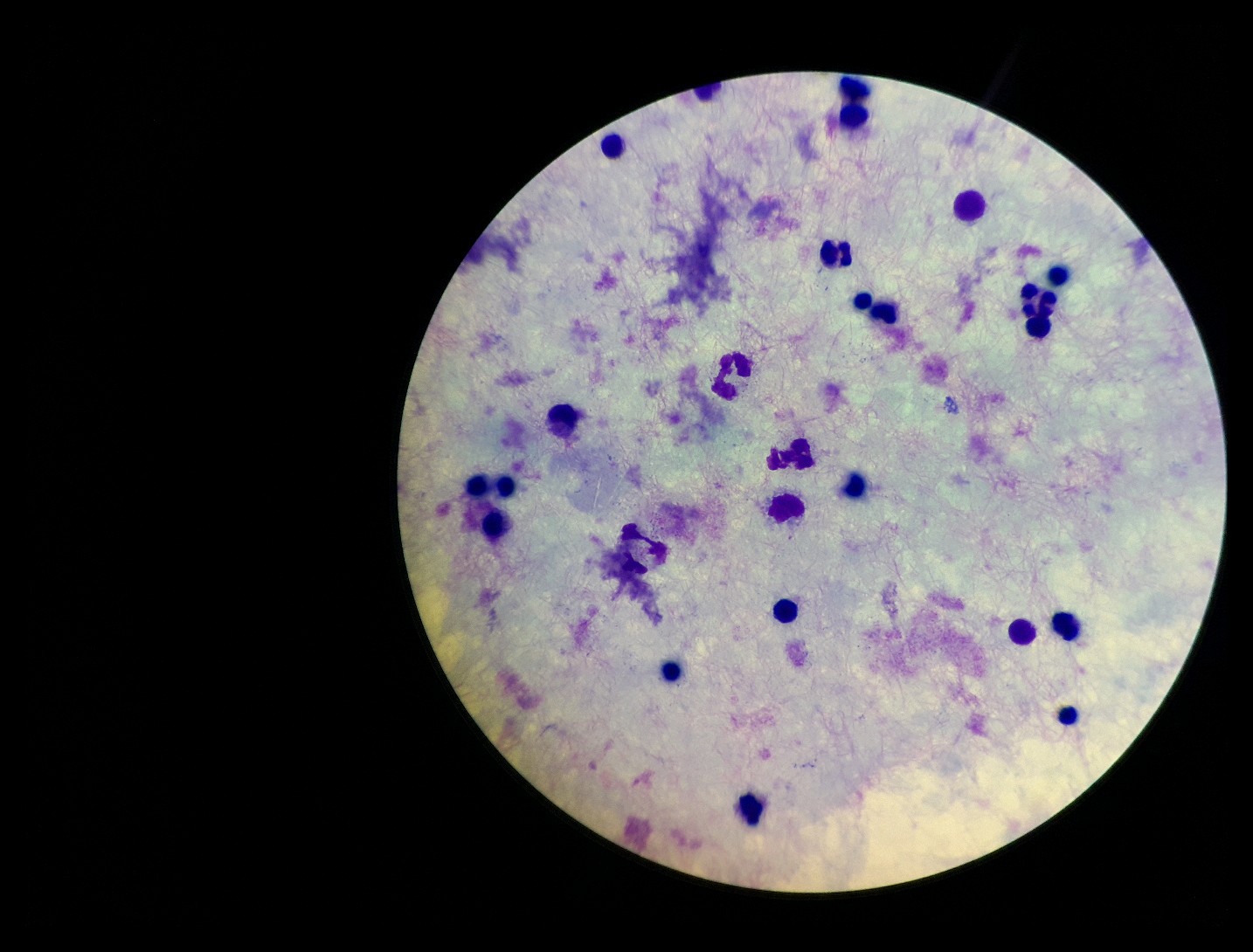

Supplement: Supplementary file 2 — Additional file 2: Overlay_images.zip: Example images showing overlays of blood smear images with detected parasite candidates. [file 12936_2023_4446_MOESM2_ESM.zip › 0002a_1/20210308_084123_Thick_result.jpg]

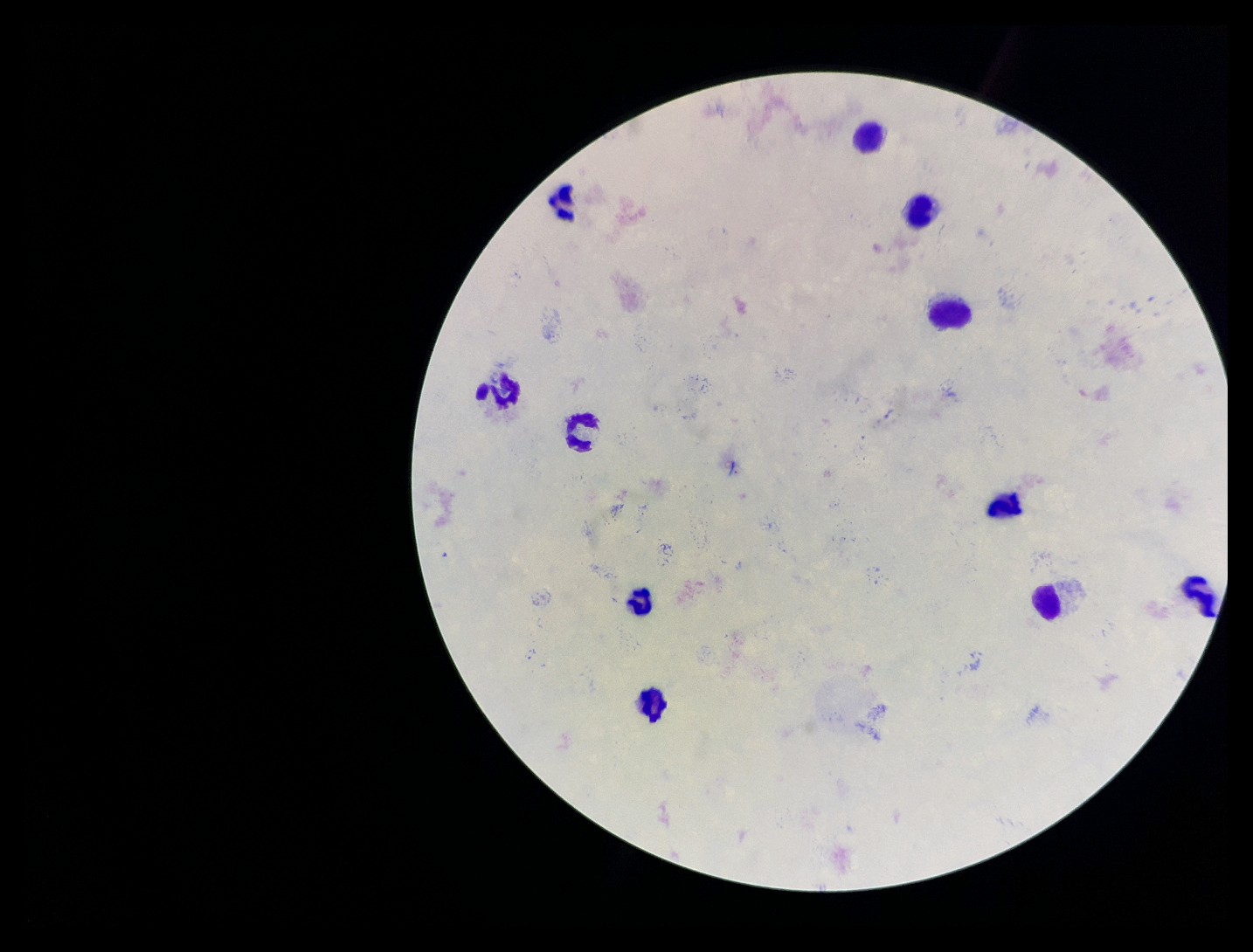

Supplement: Supplementary file 2 — Additional file 2: Overlay_images.zip: Example images showing overlays of blood smear images with detected parasite candidates. [file 12936_2023_4446_MOESM2_ESM.zip › 0004a_1/20210314_134531_Thick_result.jpg]

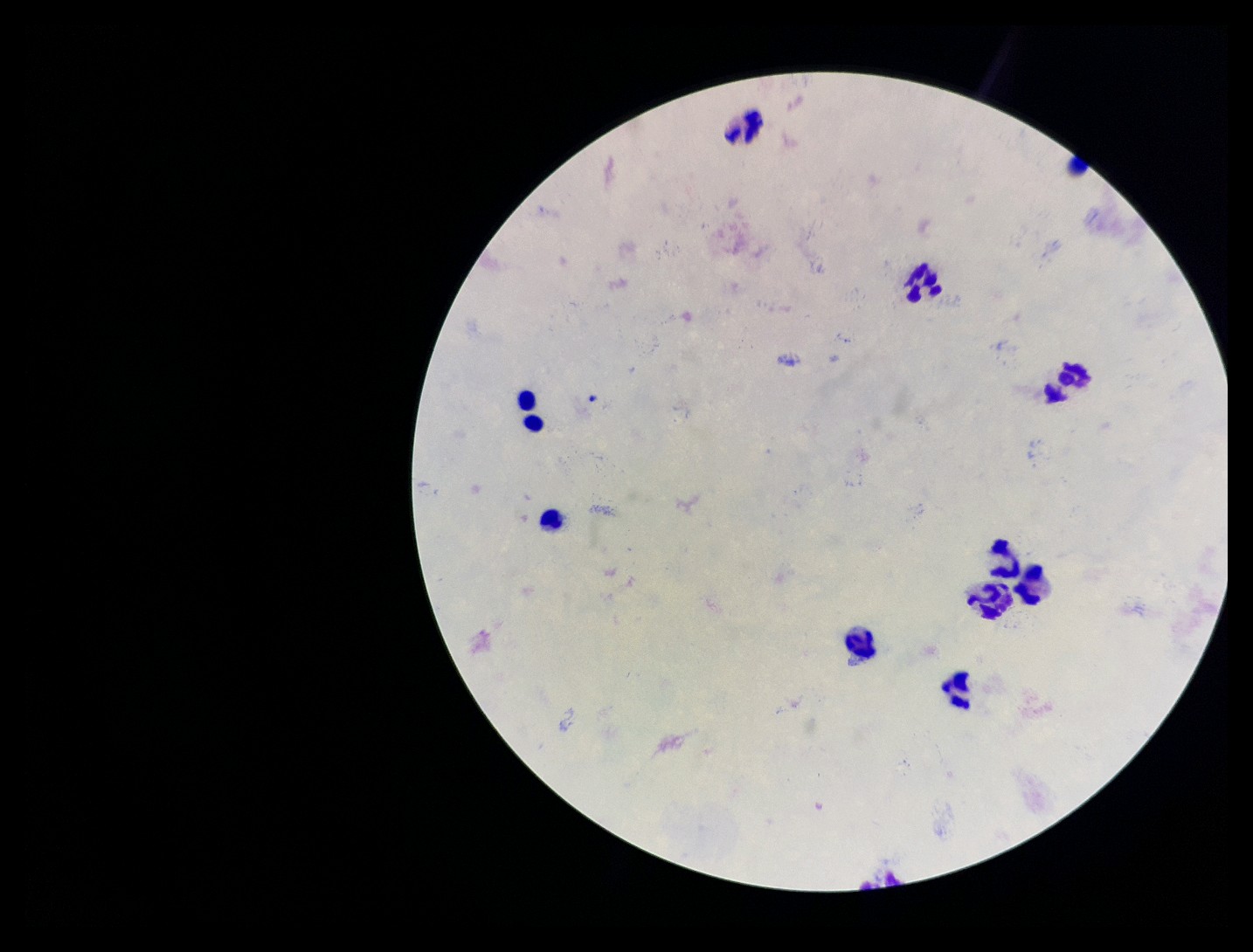

Supplement: Supplementary file 2 — Additional file 2: Overlay_images.zip: Example images showing overlays of blood smear images with detected parasite candidates. [file 12936_2023_4446_MOESM2_ESM.zip › 0004a_1/20210314_134553_Thick_result.jpg]

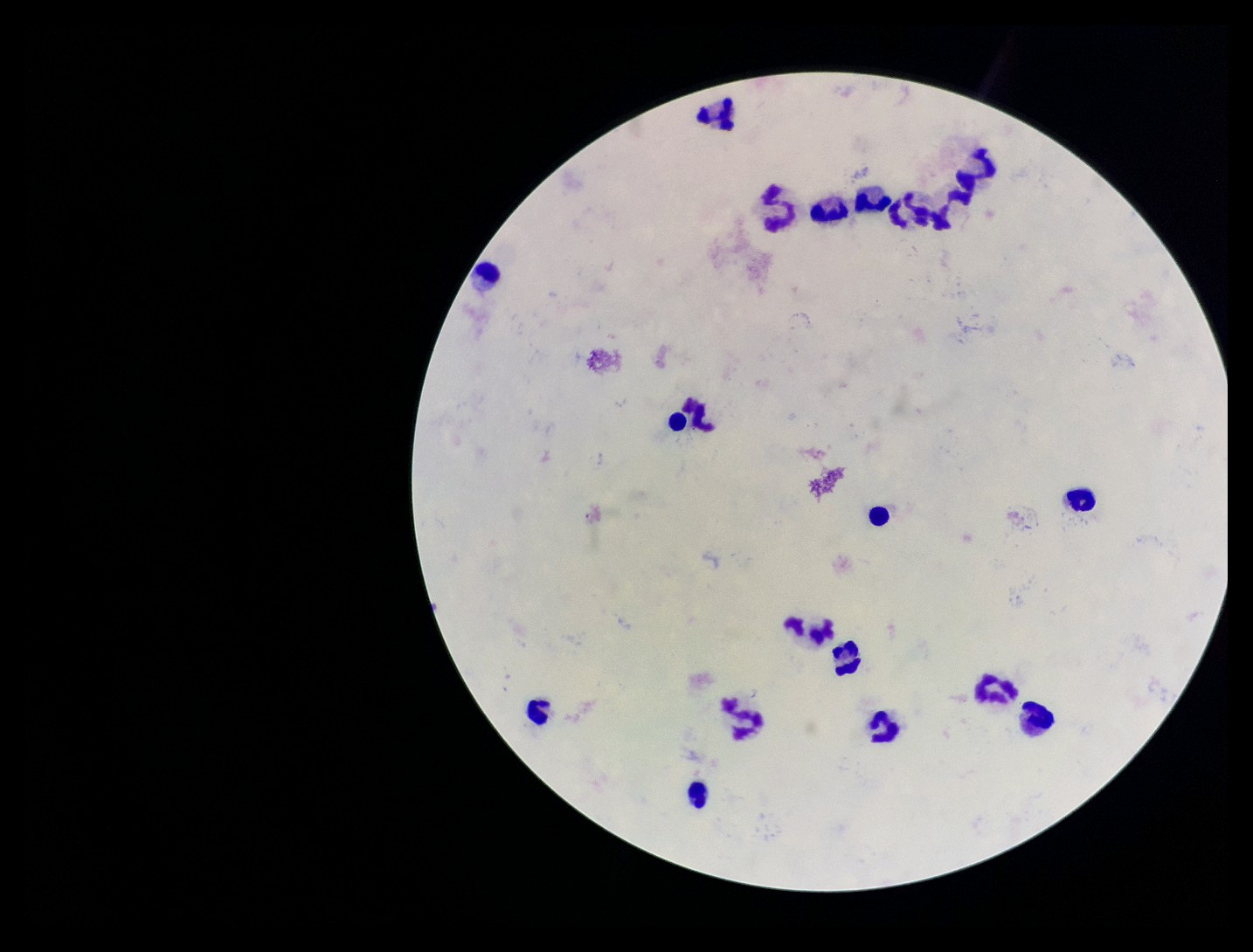

Supplement: Supplementary file 2 — Additional file 2: Overlay_images.zip: Example images showing overlays of blood smear images with detected parasite candidates. [file 12936_2023_4446_MOESM2_ESM.zip › 0004a_1/20210314_134620_Thick_result.jpg]

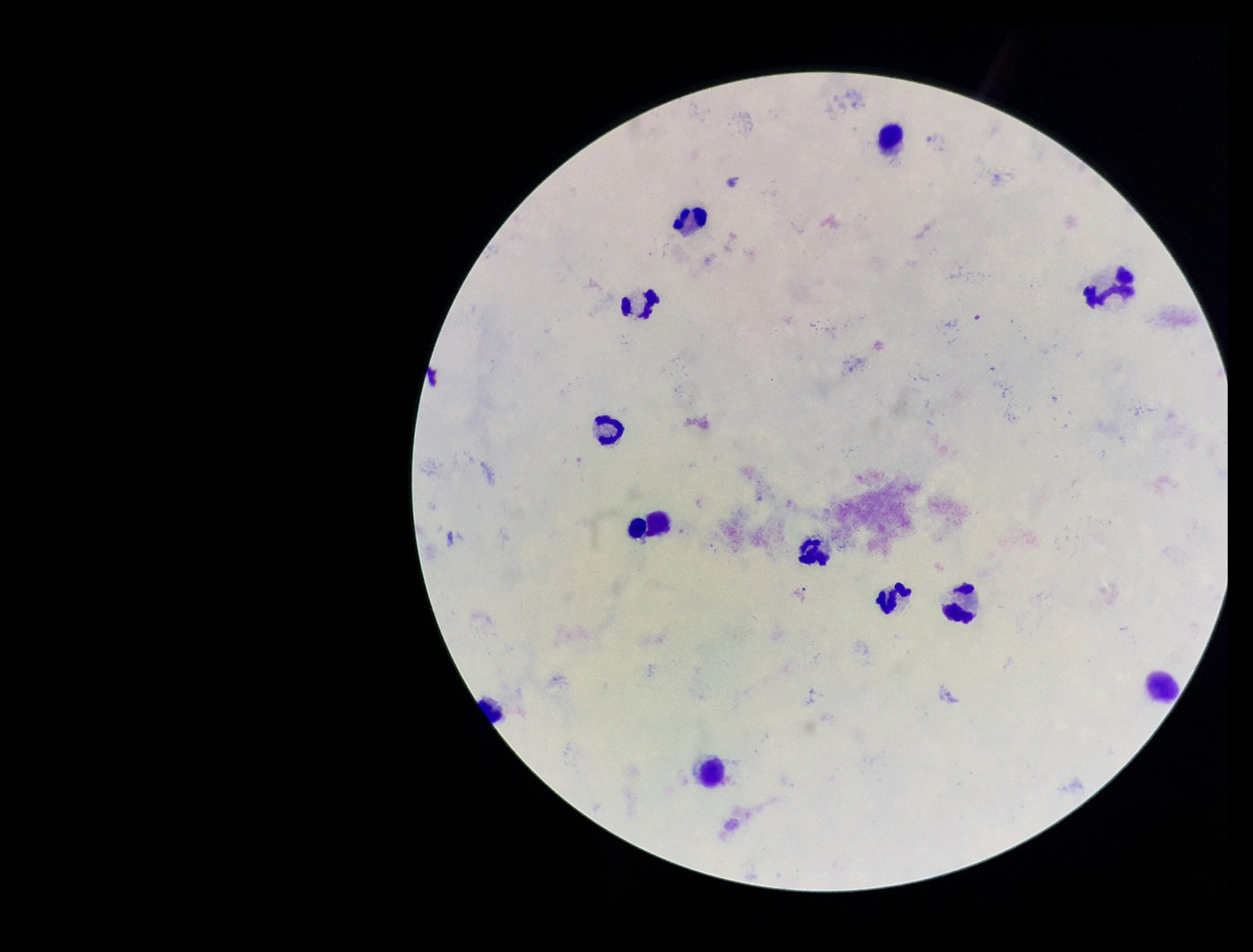

Supplement: Supplementary file 2 — Additional file 2: Overlay_images.zip: Example images showing overlays of blood smear images with detected parasite candidates. [file 12936_2023_4446_MOESM2_ESM.zip › 0004a_1/20210314_134644_Thick_result.jpg]

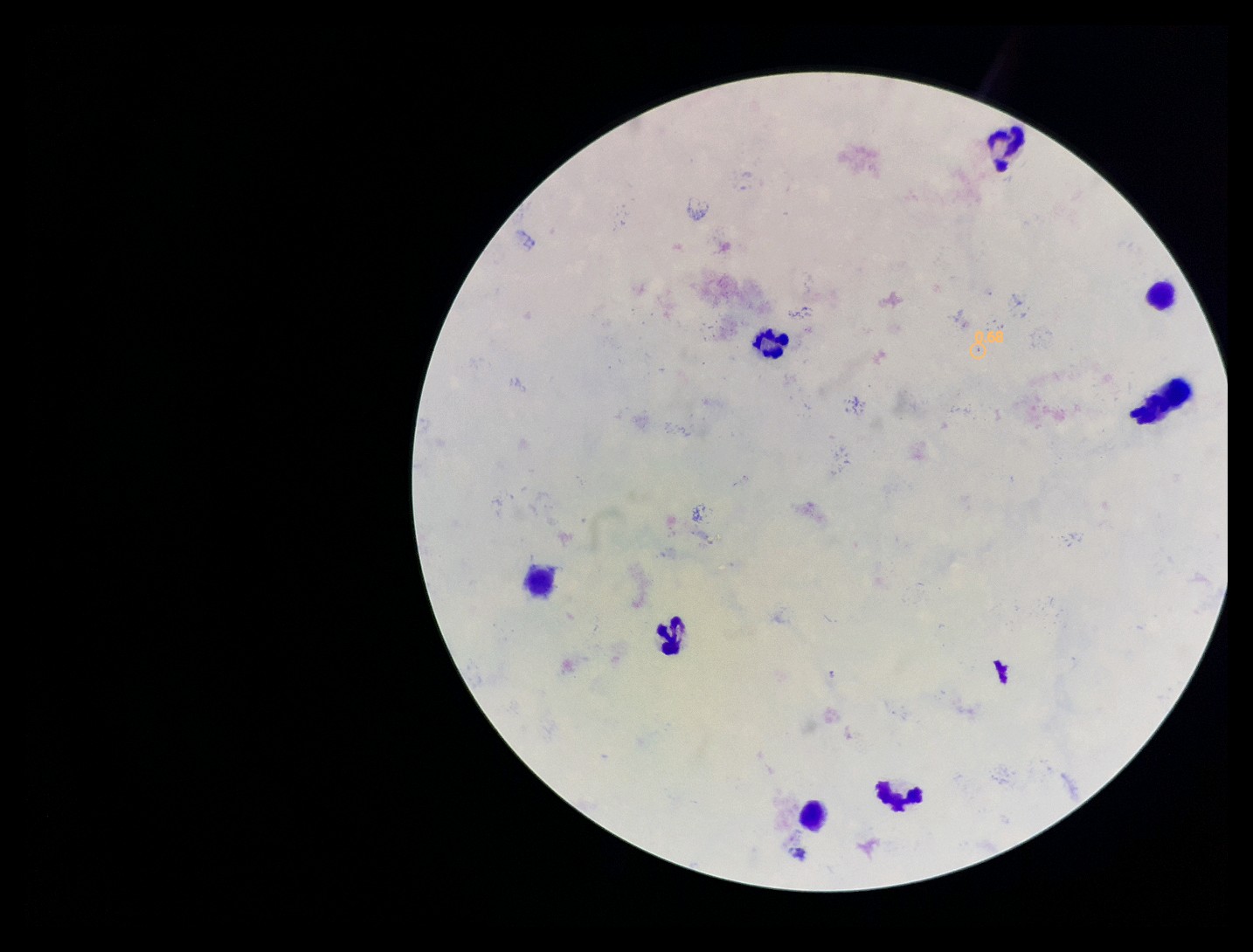

Supplement: Supplementary file 2 — Additional file 2: Overlay_images.zip: Example images showing overlays of blood smear images with detected parasite candidates. [file 12936_2023_4446_MOESM2_ESM.zip › 0004a_1/20210314_134705_Thick_result.jpg]

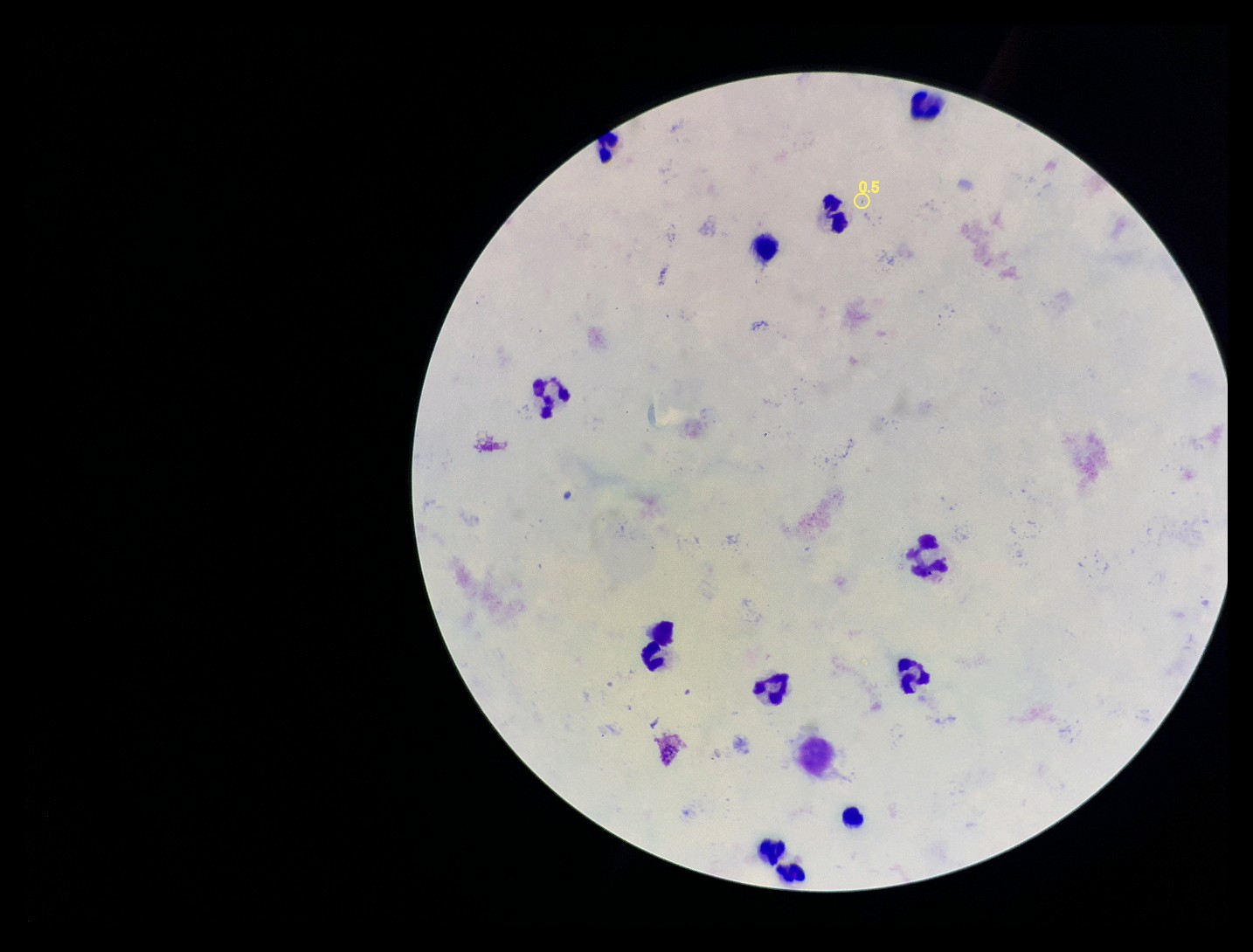

Supplement: Supplementary file 2 — Additional file 2: Overlay_images.zip: Example images showing overlays of blood smear images with detected parasite candidates. [file 12936_2023_4446_MOESM2_ESM.zip › 0004a_1/20210314_134734_Thick_result.jpg]

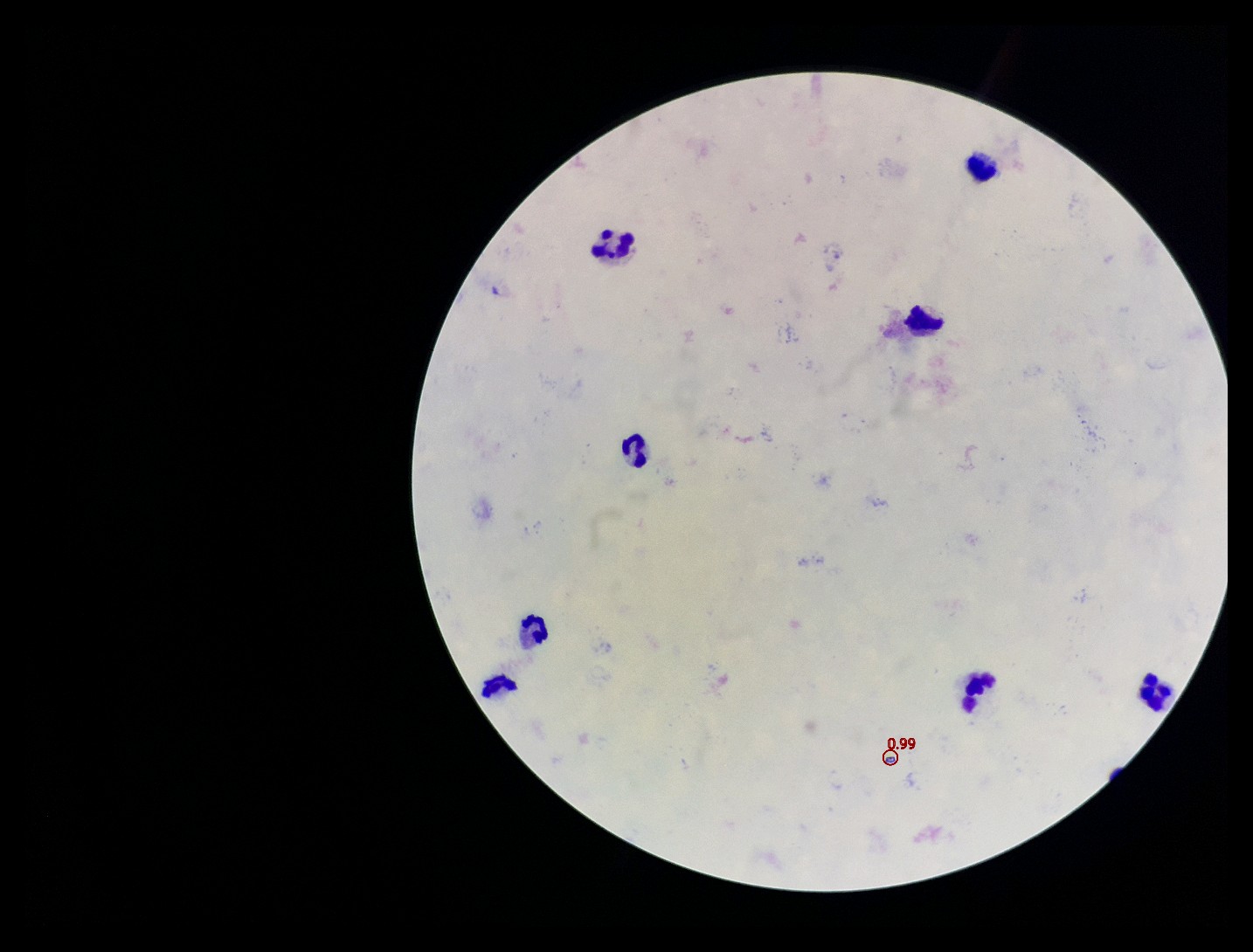

Supplement: Supplementary file 2 — Additional file 2: Overlay_images.zip: Example images showing overlays of blood smear images with detected parasite candidates. [file 12936_2023_4446_MOESM2_ESM.zip › 0004a_1/20210314_134758_Thick_result.jpg]

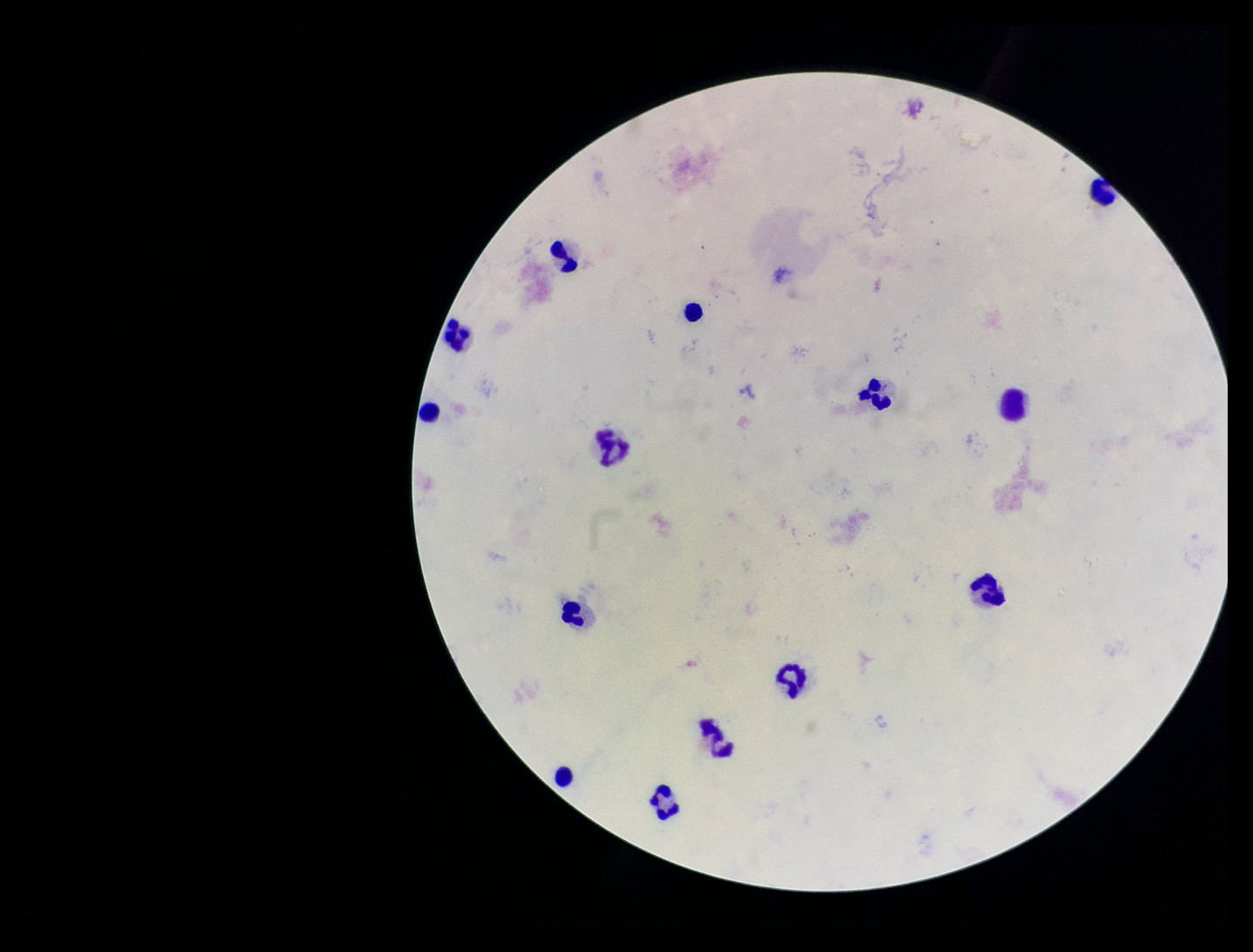

Supplement: Supplementary file 2 — Additional file 2: Overlay_images.zip: Example images showing overlays of blood smear images with detected parasite candidates. [file 12936_2023_4446_MOESM2_ESM.zip › 0004a_1/20210314_134818_Thick_result.jpg]

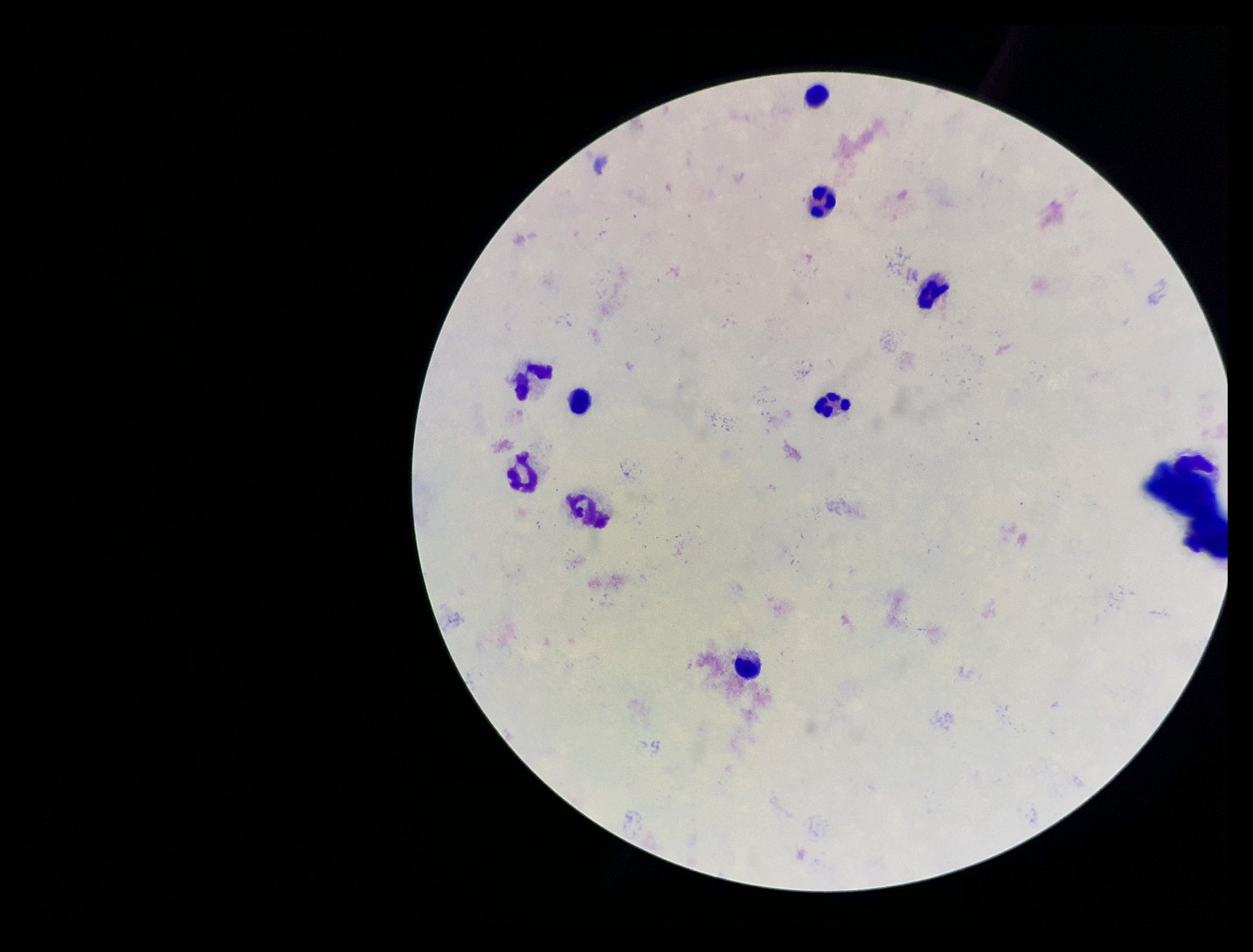

Supplement: Supplementary file 2 — Additional file 2: Overlay_images.zip: Example images showing overlays of blood smear images with detected parasite candidates. [file 12936_2023_4446_MOESM2_ESM.zip › 0004a_1/20210314_134843_Thick_result.jpg]

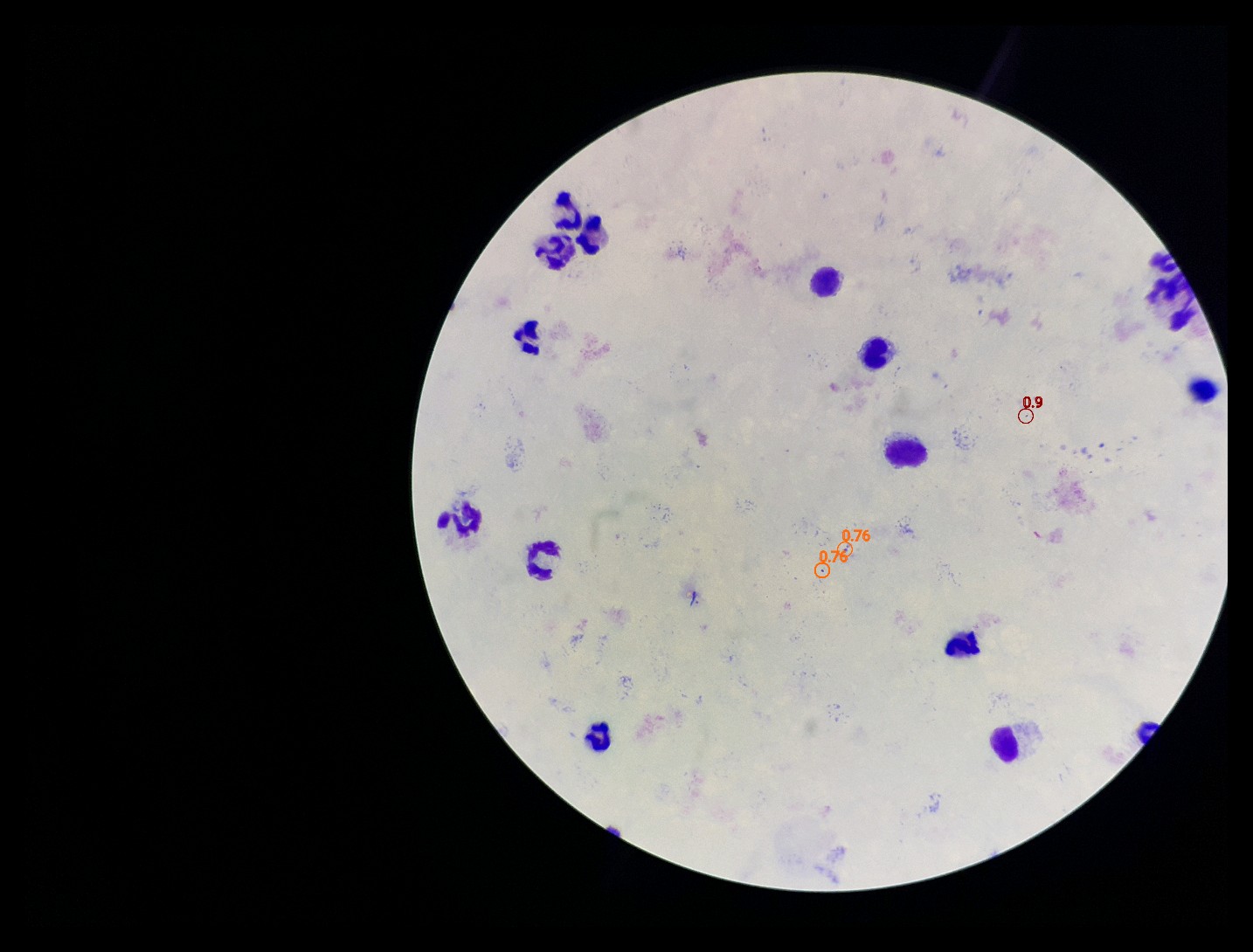

Supplement: Supplementary file 2 — Additional file 2: Overlay_images.zip: Example images showing overlays of blood smear images with detected parasite candidates. [file 12936_2023_4446_MOESM2_ESM.zip › 0004a_1/20210314_134909_Thick_result.jpg]

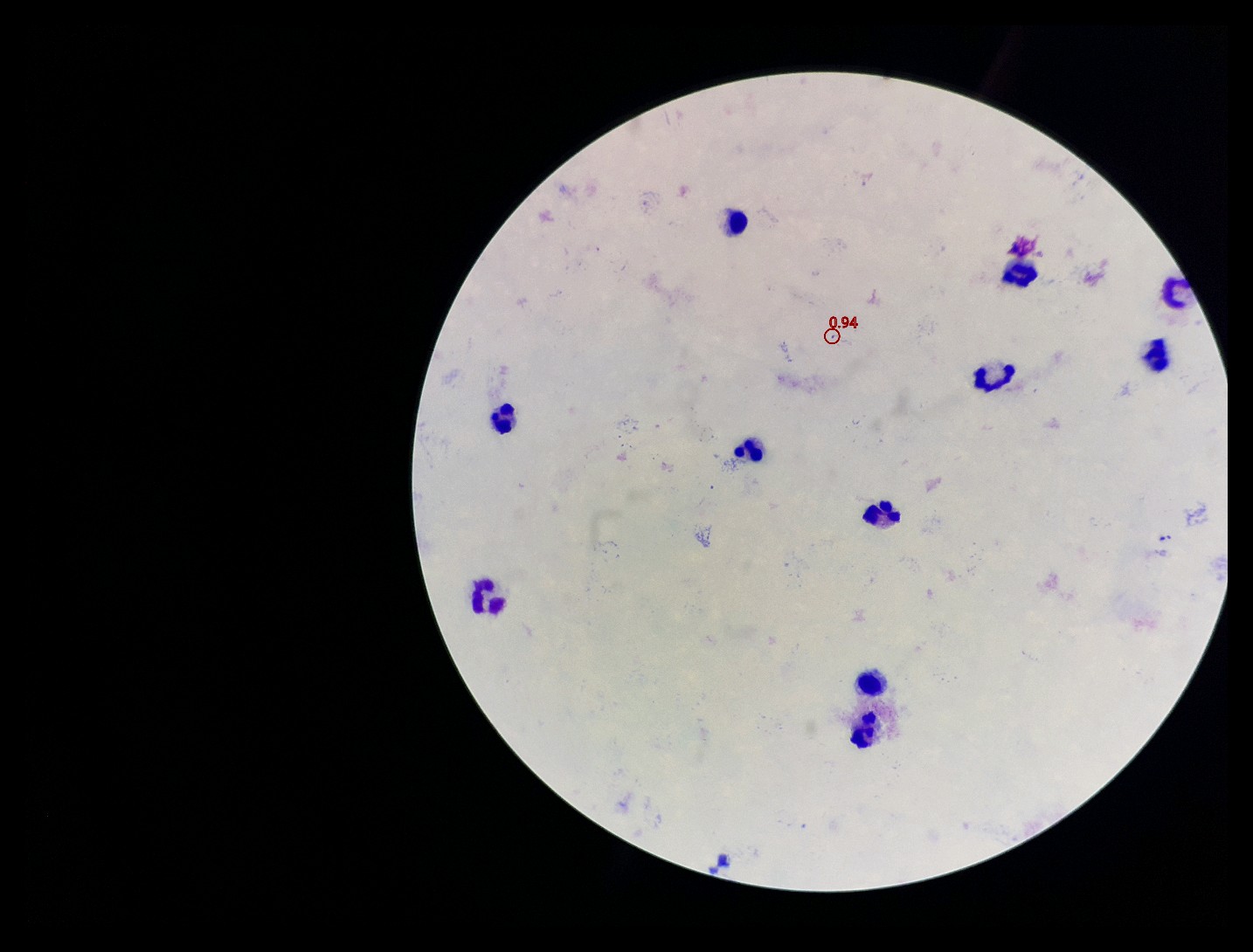

Supplement: Supplementary file 2 — Additional file 2: Overlay_images.zip: Example images showing overlays of blood smear images with detected parasite candidates. [file 12936_2023_4446_MOESM2_ESM.zip › 0004a_1/20210314_134934_Thick_result.jpg]

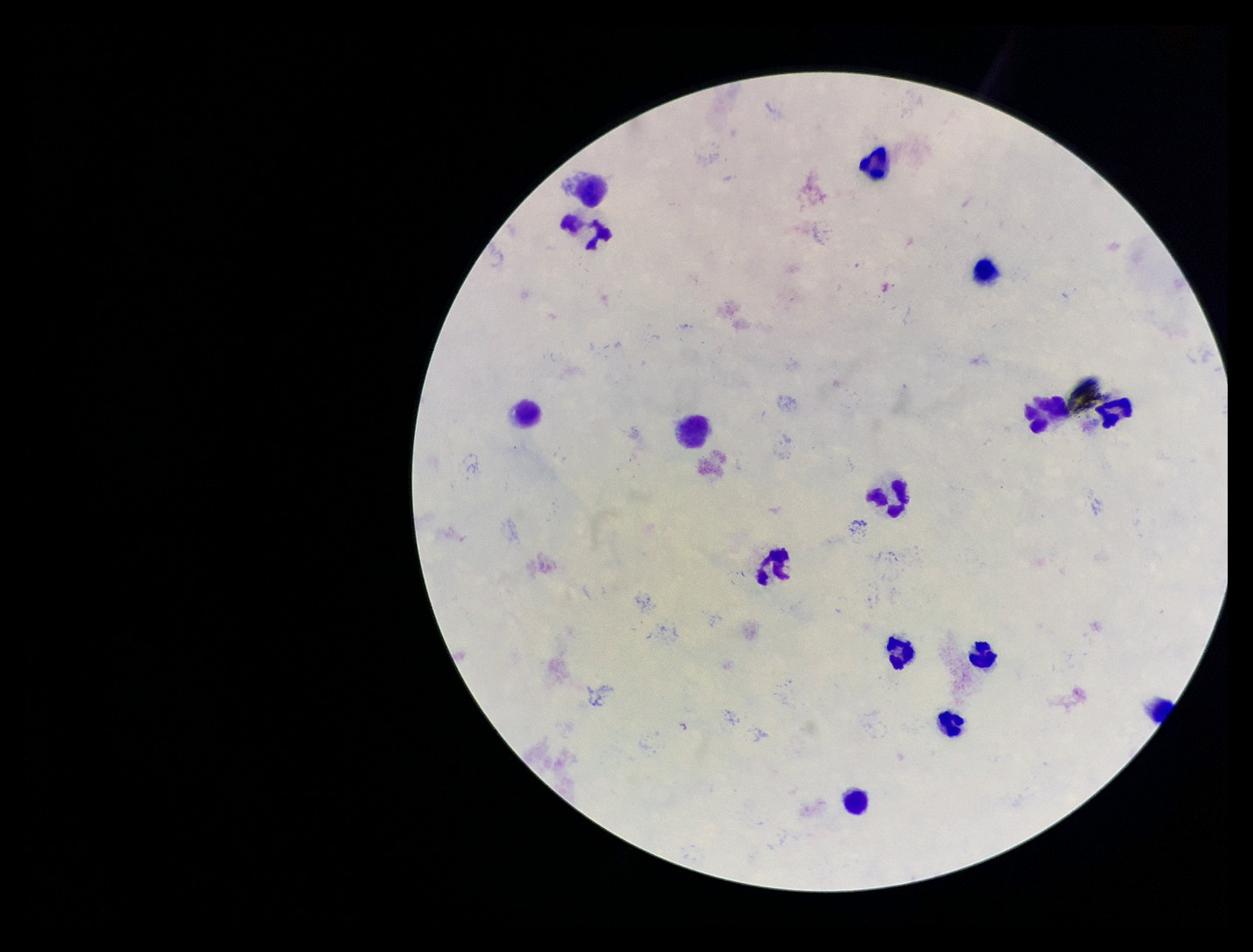

Supplement: Supplementary file 2 — Additional file 2: Overlay_images.zip: Example images showing overlays of blood smear images with detected parasite candidates. [file 12936_2023_4446_MOESM2_ESM.zip › 0004a_1/20210314_134955_Thick_result.jpg]

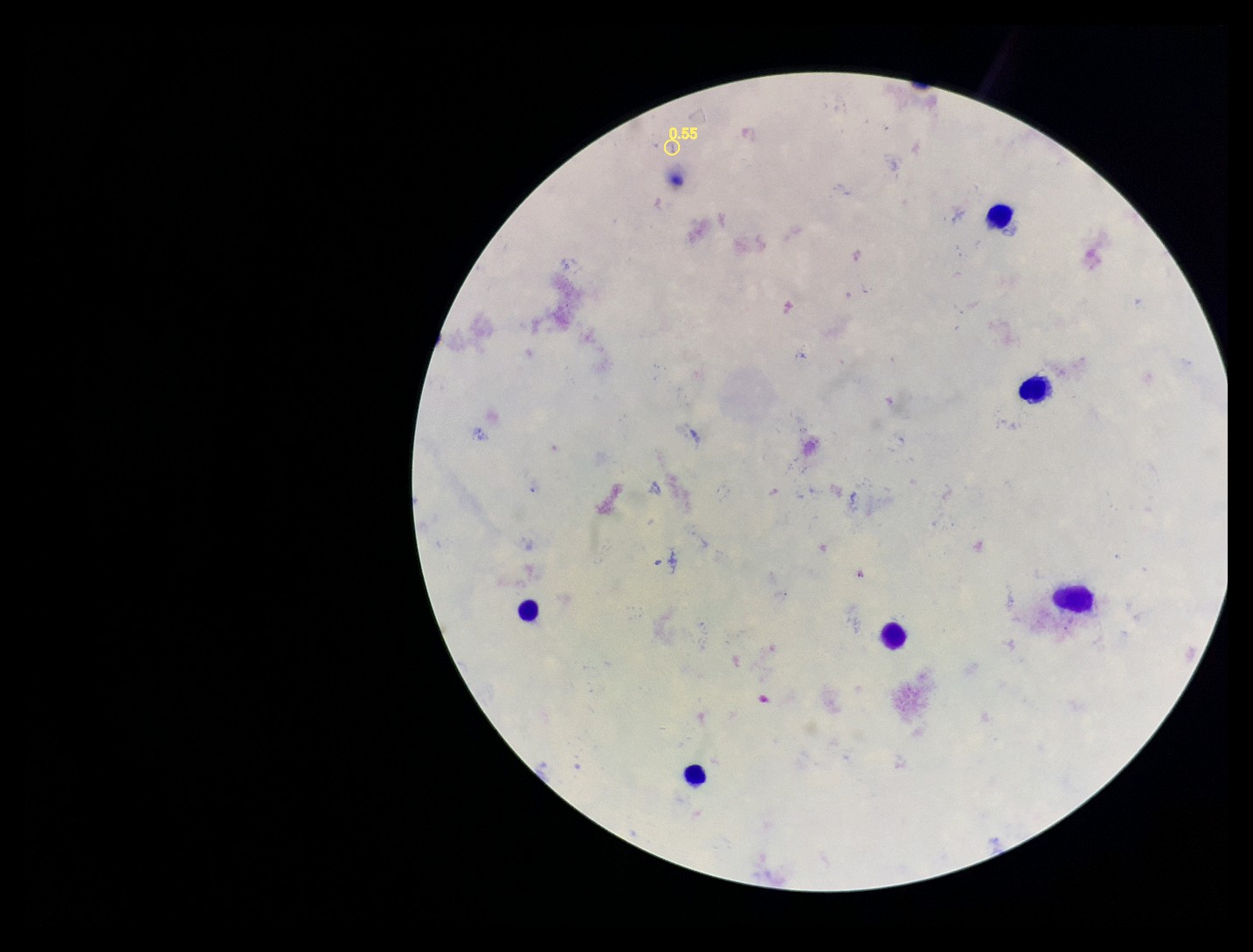

Supplement: Supplementary file 2 — Additional file 2: Overlay_images.zip: Example images showing overlays of blood smear images with detected parasite candidates. [file 12936_2023_4446_MOESM2_ESM.zip › 0004a_1/20210314_135015_Thick_result.jpg]

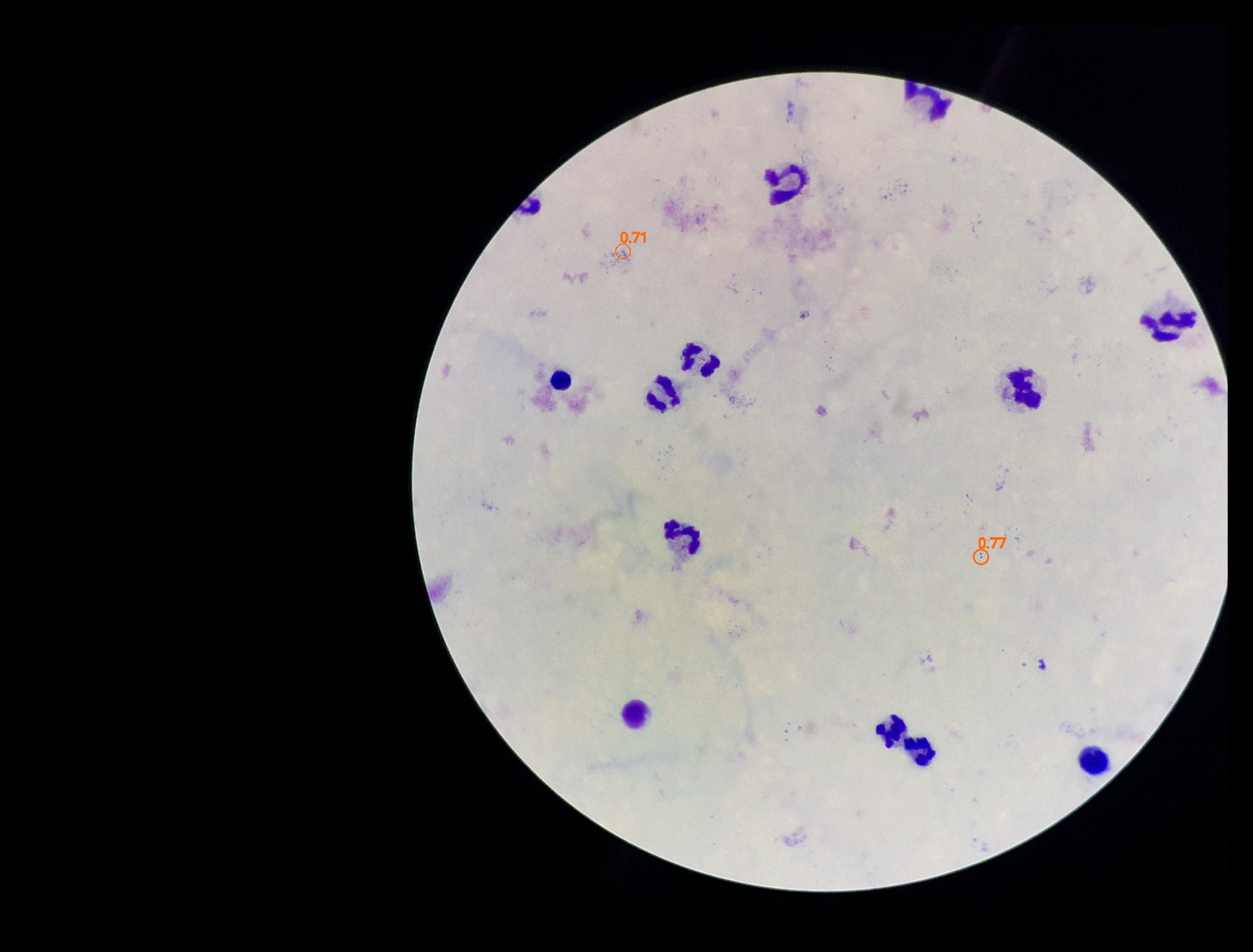

Supplement: Supplementary file 2 — Additional file 2: Overlay_images.zip: Example images showing overlays of blood smear images with detected parasite candidates. [file 12936_2023_4446_MOESM2_ESM.zip › 0004a_1/20210314_135039_Thick_result.jpg]

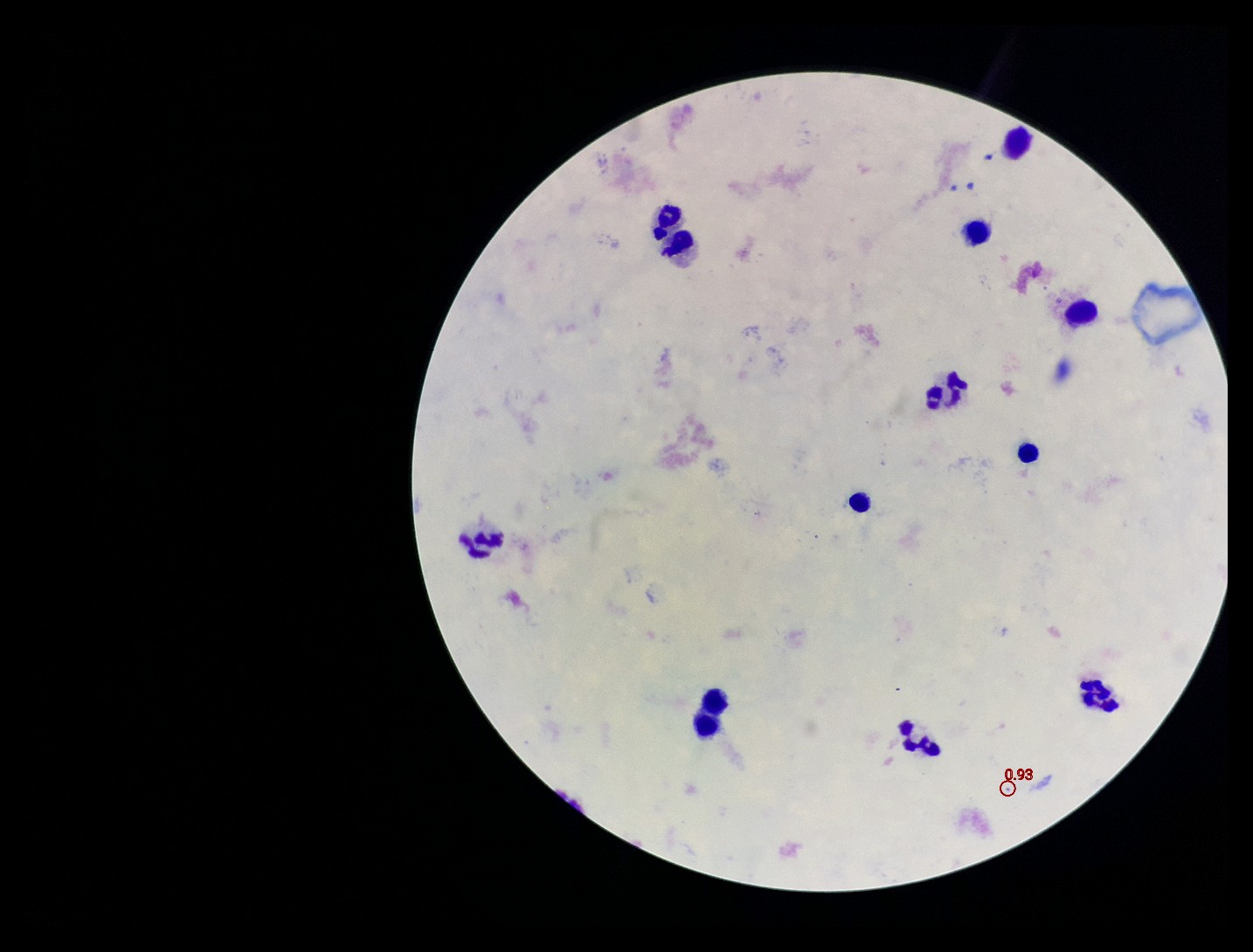

Supplement: Supplementary file 2 — Additional file 2: Overlay_images.zip: Example images showing overlays of blood smear images with detected parasite candidates. [file 12936_2023_4446_MOESM2_ESM.zip › 0004a_1/20210314_135107_Thick_result.jpg]

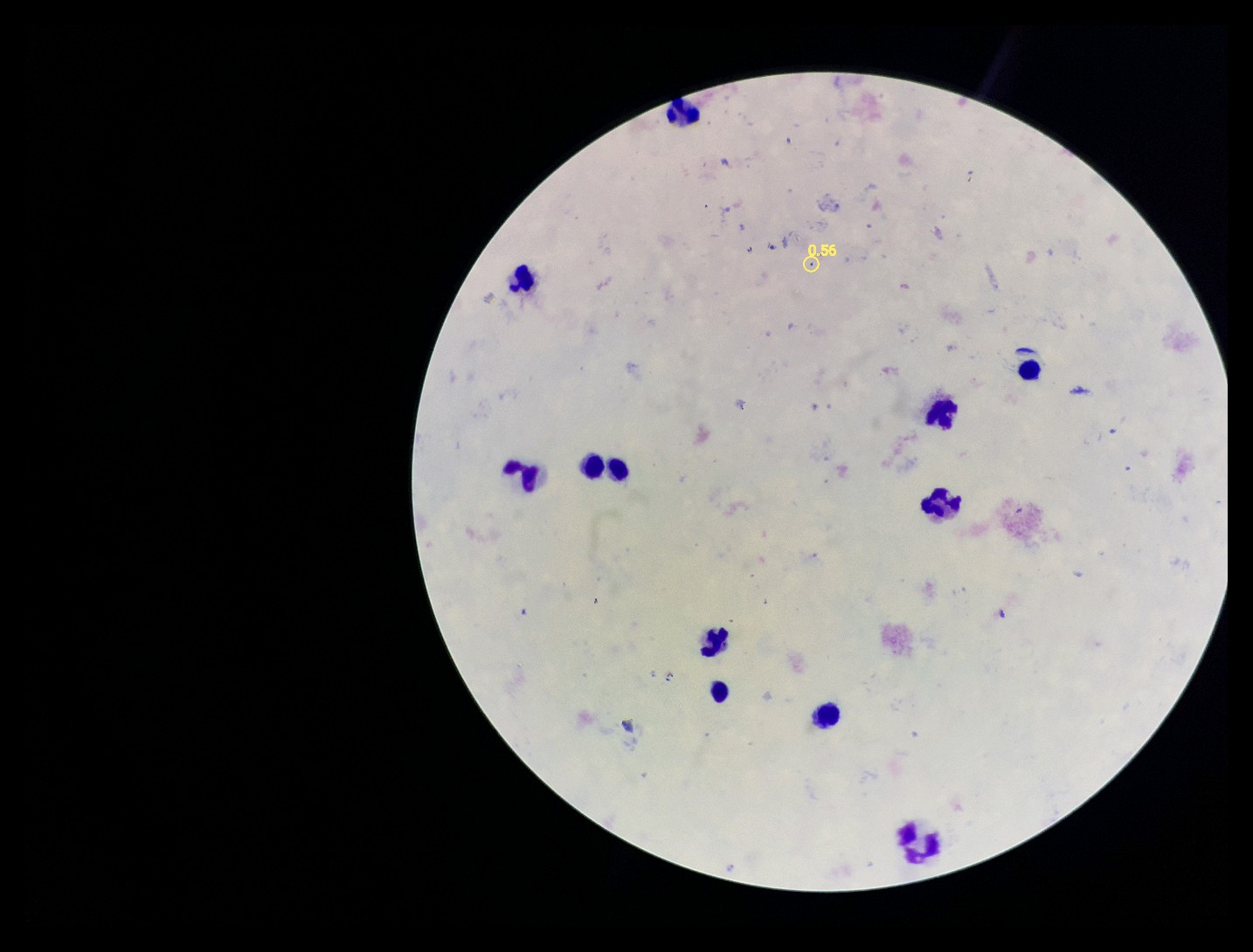

Supplement: Supplementary file 2 — Additional file 2: Overlay_images.zip: Example images showing overlays of blood smear images with detected parasite candidates. [file 12936_2023_4446_MOESM2_ESM.zip › 0004a_1/20210314_135130_Thick_result.jpg]

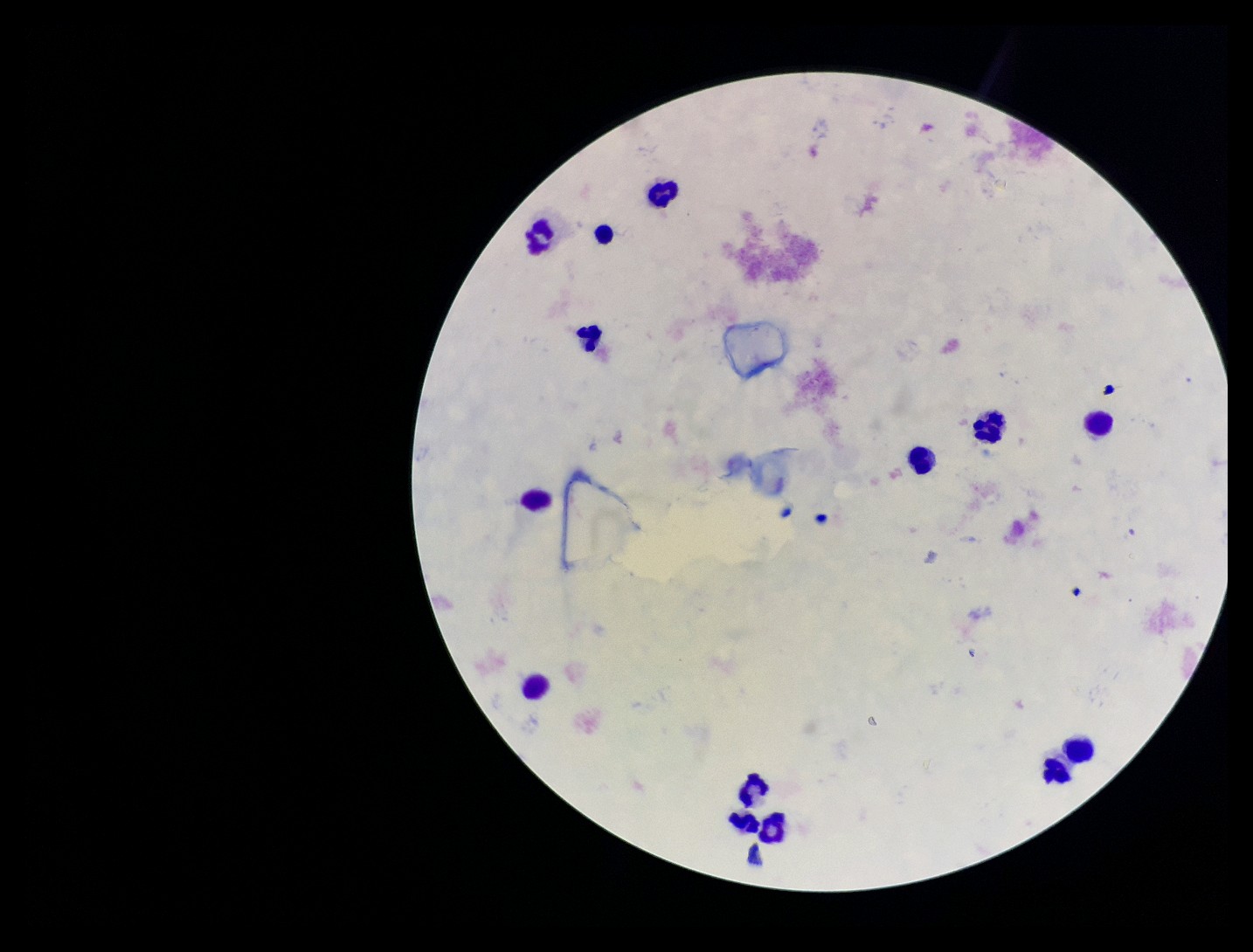

Supplement: Supplementary file 2 — Additional file 2: Overlay_images.zip: Example images showing overlays of blood smear images with detected parasite candidates. [file 12936_2023_4446_MOESM2_ESM.zip › 0004a_1/20210314_135155_Thick_result.jpg]

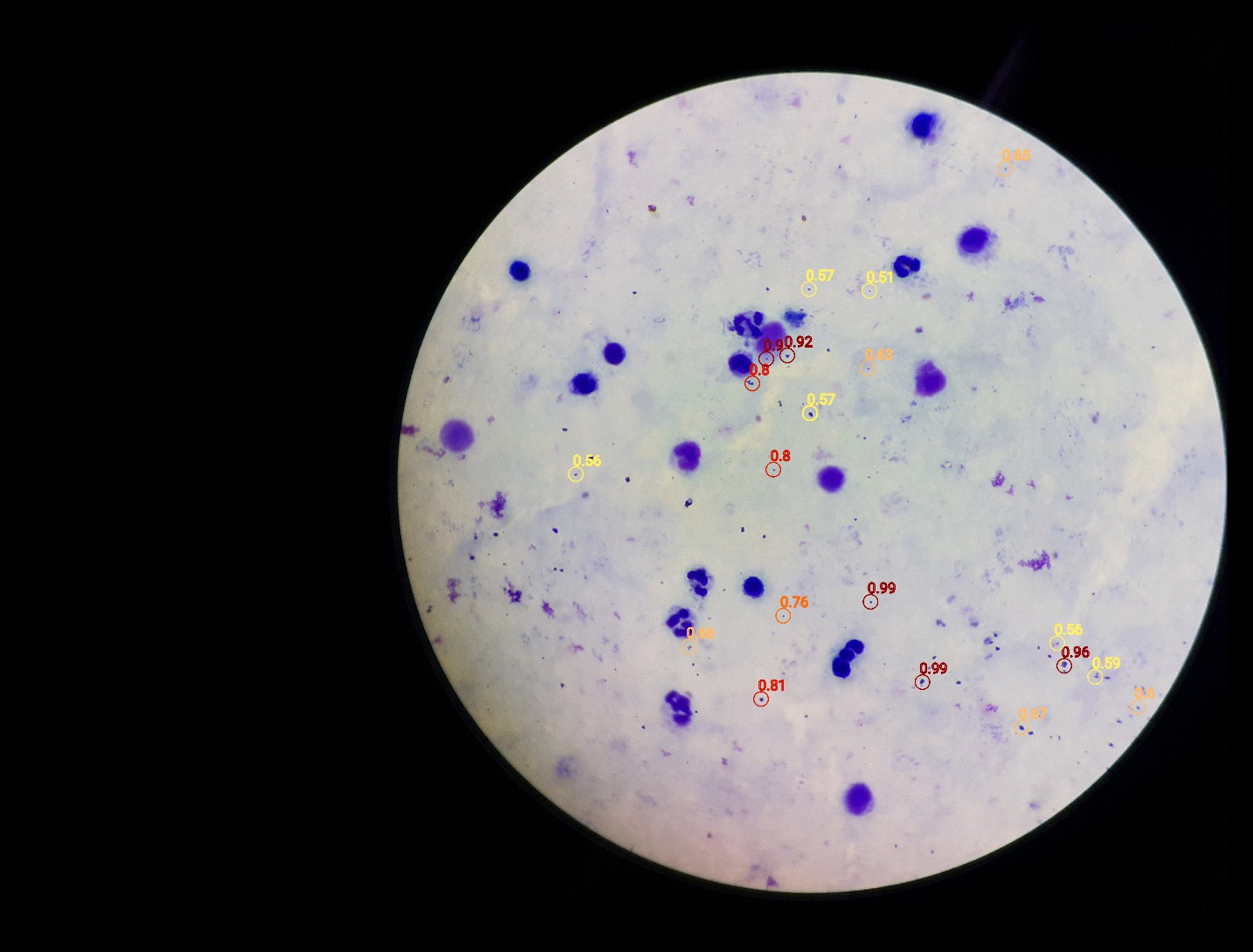

Supplement: Supplementary file 2 — Additional file 2: Overlay_images.zip: Example images showing overlays of blood smear images with detected parasite candidates. [file 12936_2023_4446_MOESM2_ESM.zip › 0006a_1/20210308_093115_Thick_result.jpg]

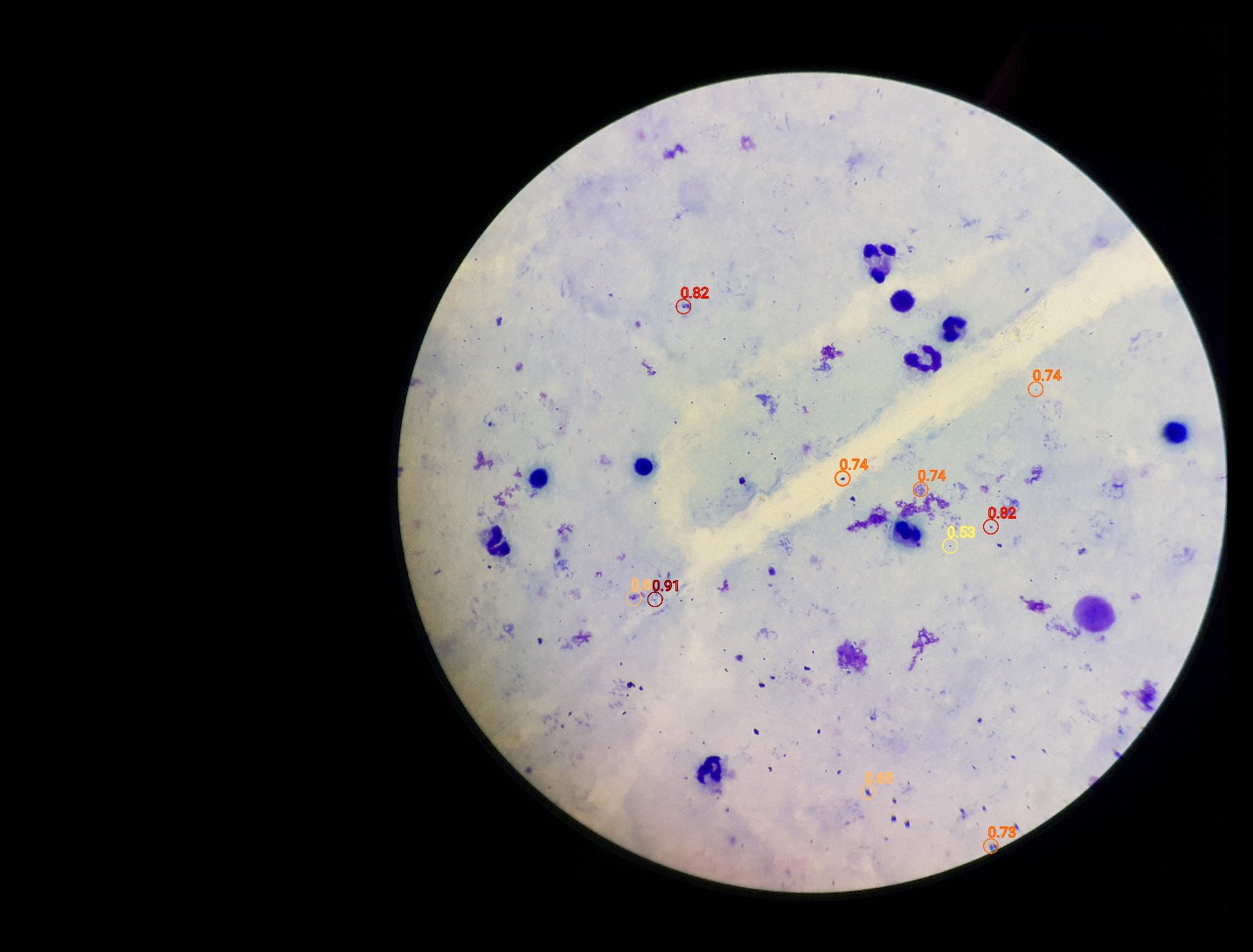

Supplement: Supplementary file 2 — Additional file 2: Overlay_images.zip: Example images showing overlays of blood smear images with detected parasite candidates. [file 12936_2023_4446_MOESM2_ESM.zip › 0006a_1/20210308_093141_Thick_result.jpg]

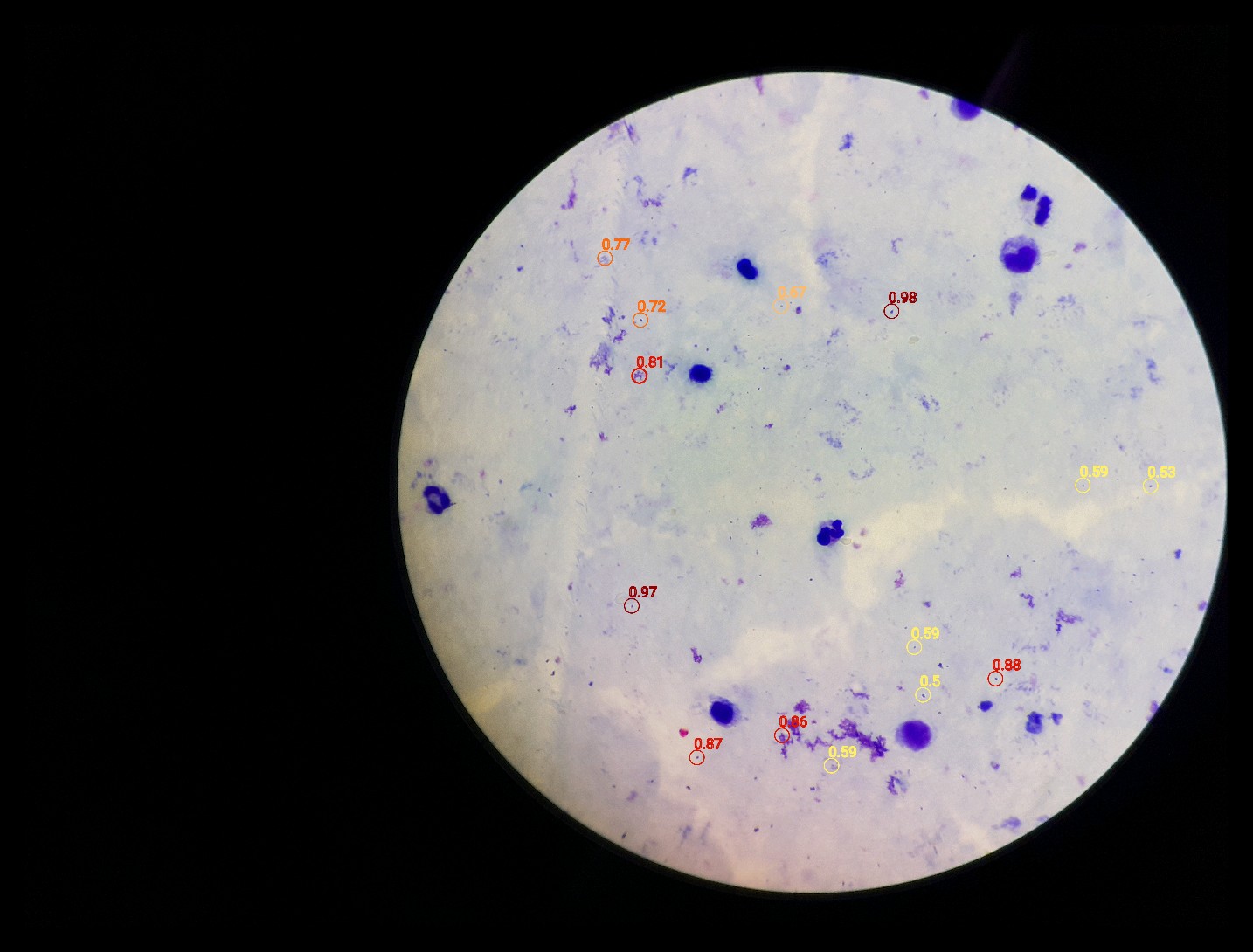

Supplement: Supplementary file 2 — Additional file 2: Overlay_images.zip: Example images showing overlays of blood smear images with detected parasite candidates. [file 12936_2023_4446_MOESM2_ESM.zip › 0006a_1/20210308_093205_Thick_result.jpg]

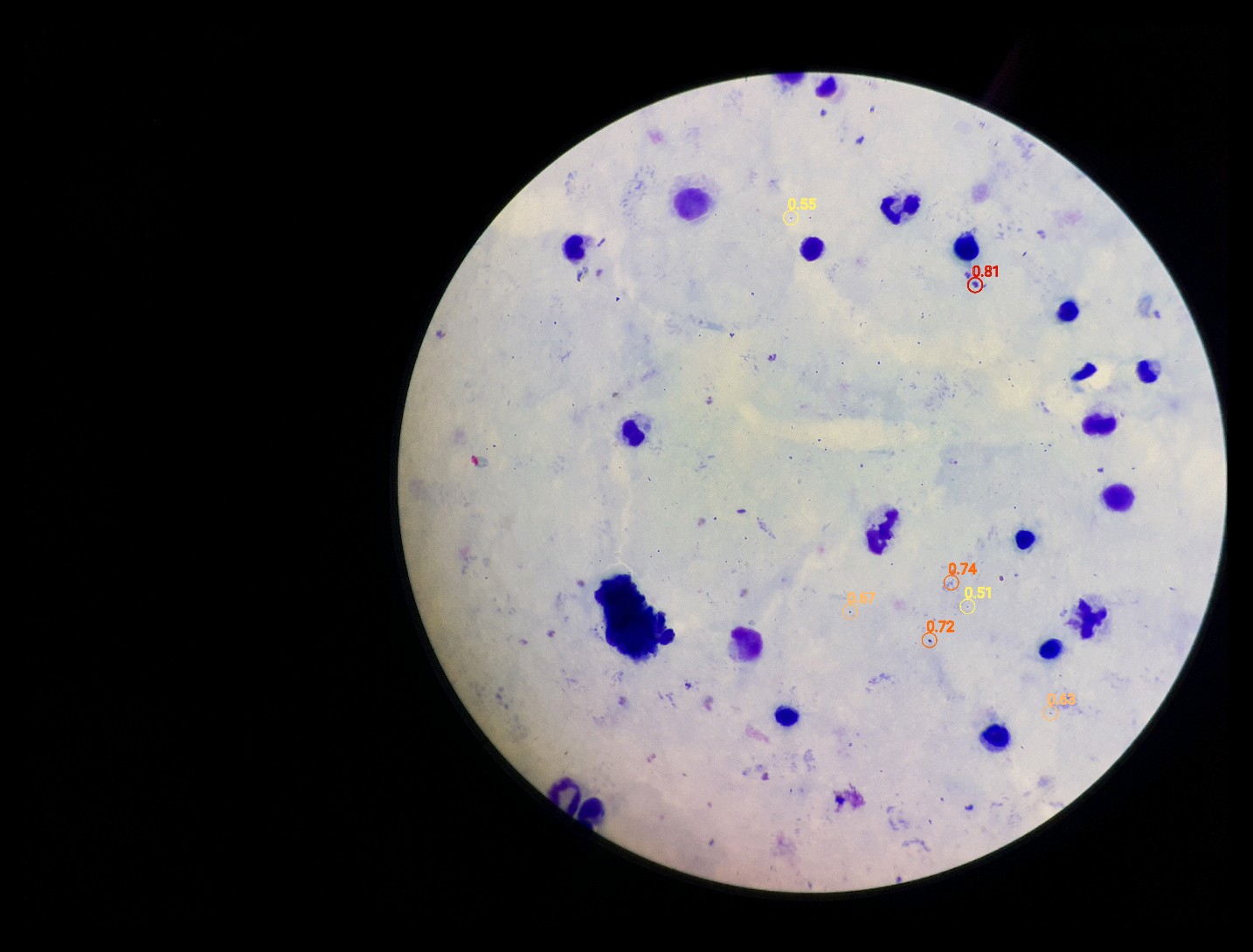

Supplement: Supplementary file 2 — Additional file 2: Overlay_images.zip: Example images showing overlays of blood smear images with detected parasite candidates. [file 12936_2023_4446_MOESM2_ESM.zip › 0006a_1/20210308_093239_Thick_result.jpg]

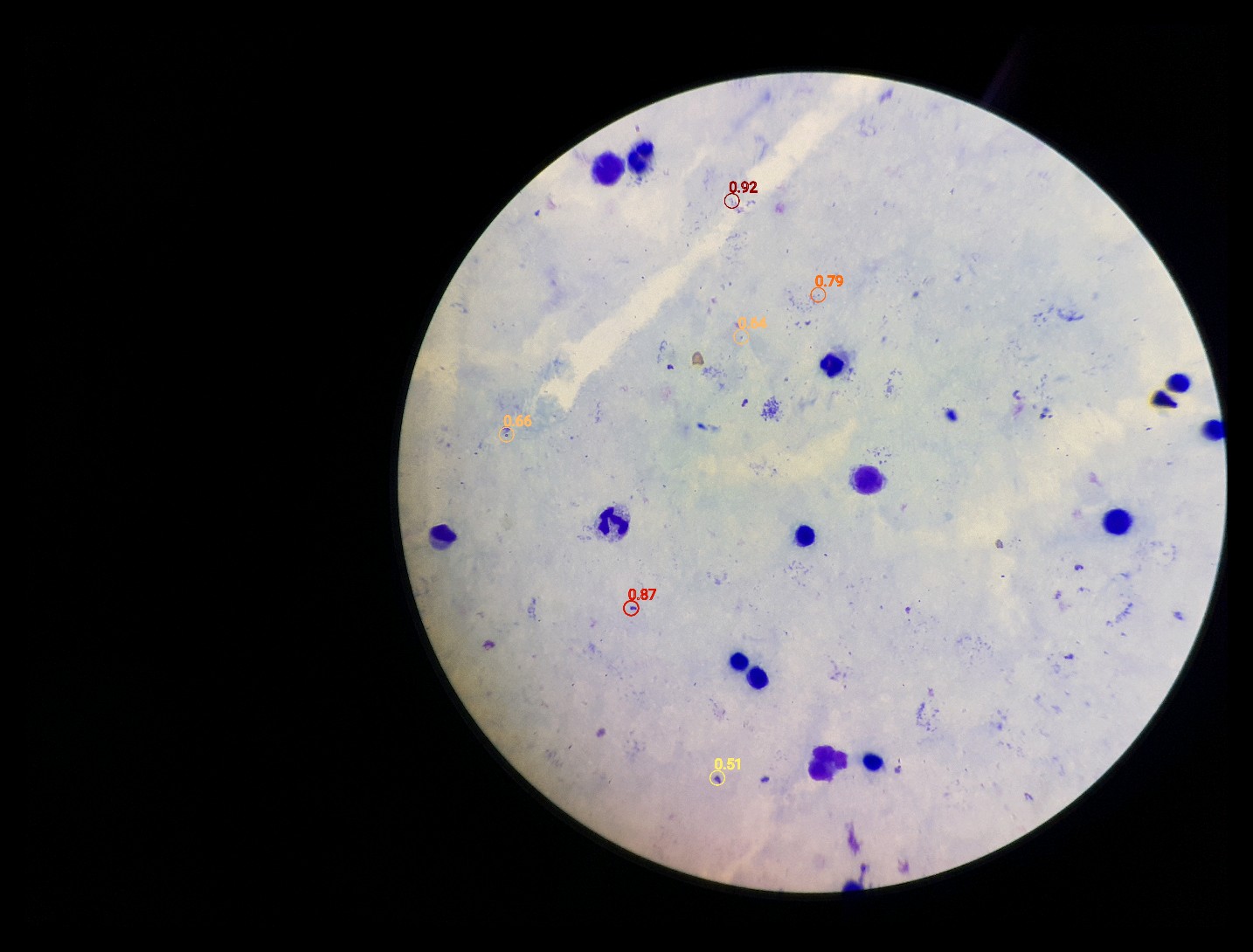

Supplement: Supplementary file 2 — Additional file 2: Overlay_images.zip: Example images showing overlays of blood smear images with detected parasite candidates. [file 12936_2023_4446_MOESM2_ESM.zip › 0006a_1/20210308_093314_Thick_result.jpg]

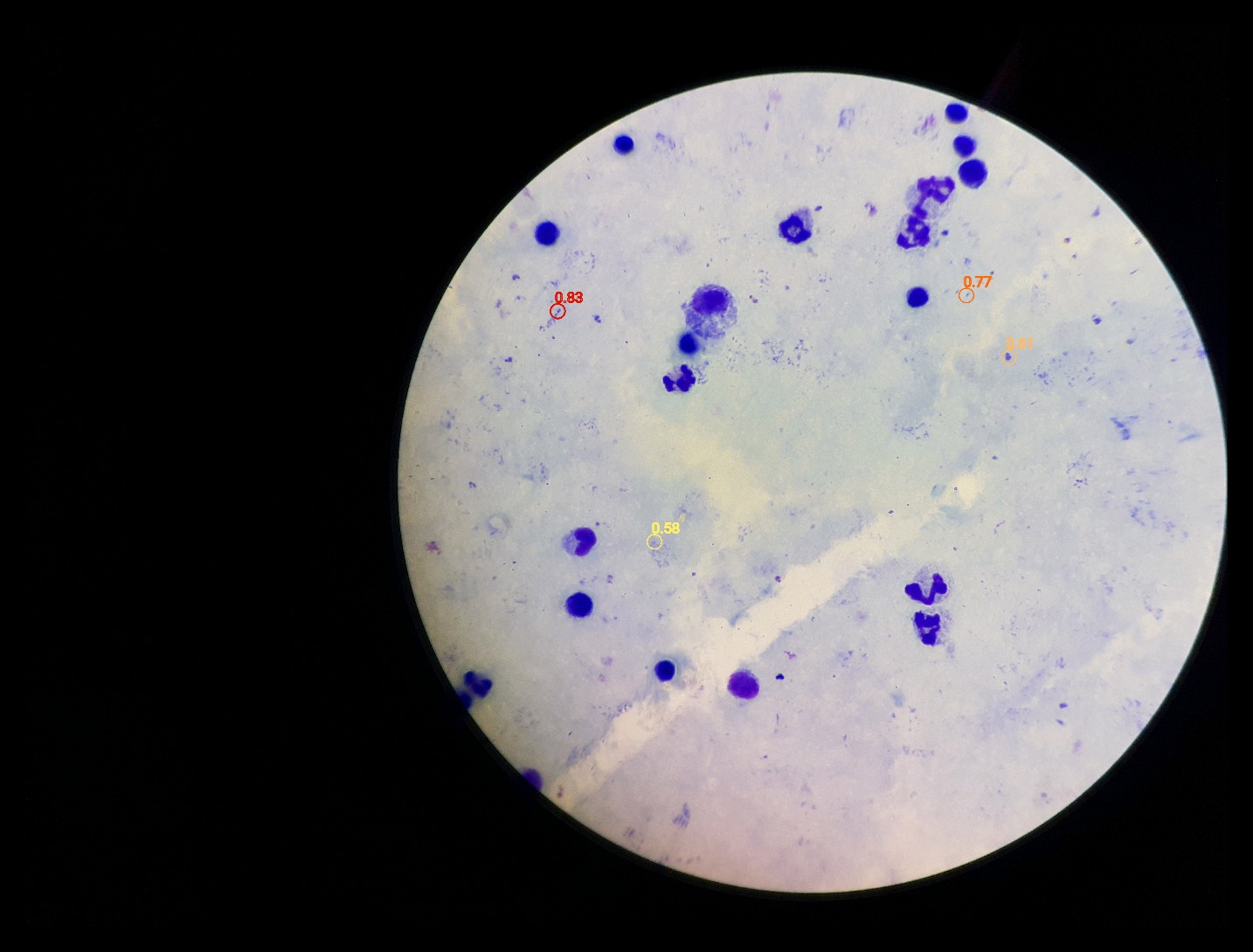

Supplement: Supplementary file 2 — Additional file 2: Overlay_images.zip: Example images showing overlays of blood smear images with detected parasite candidates. [file 12936_2023_4446_MOESM2_ESM.zip › 0006a_1/20210308_093335_Thick_result.jpg]

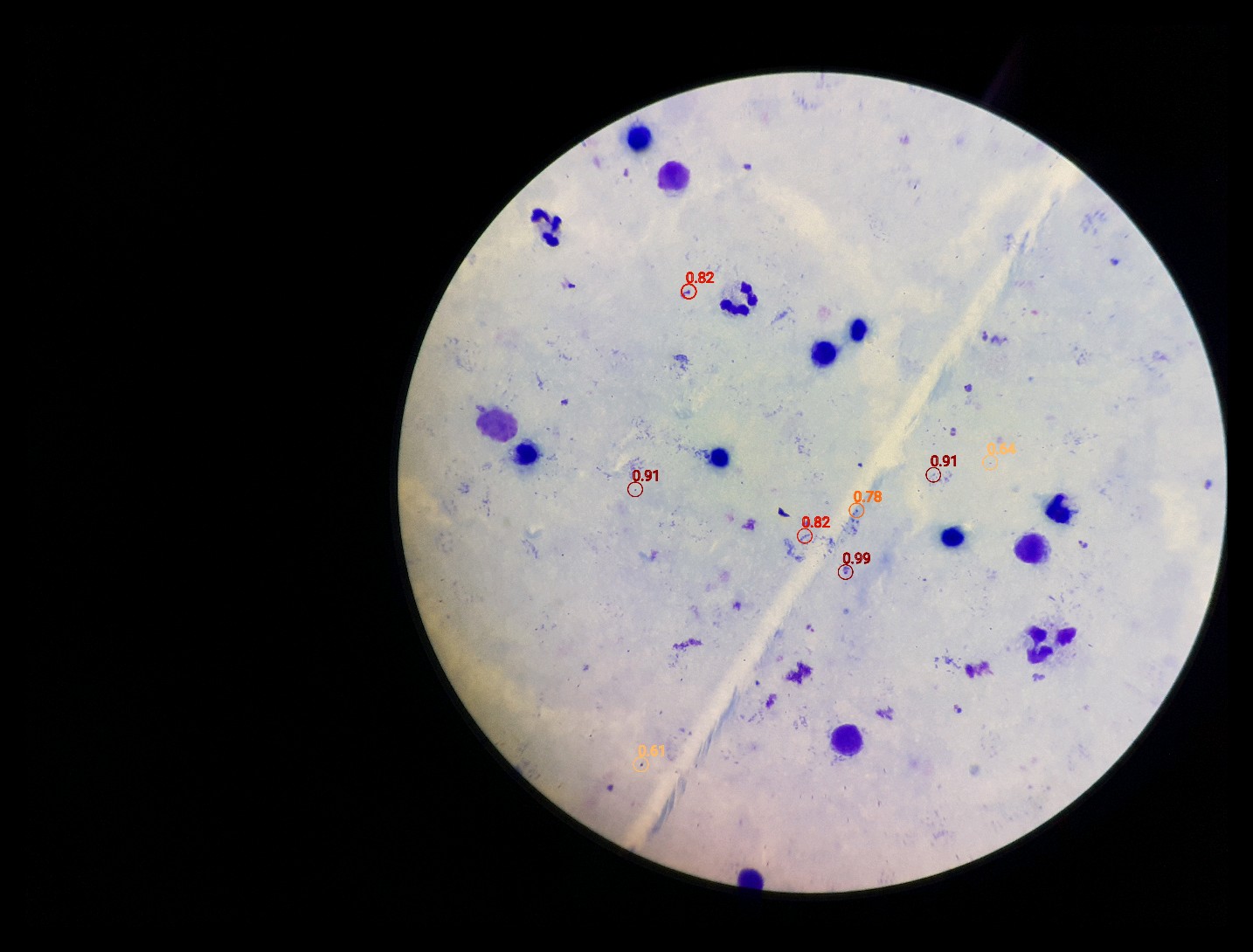

Supplement: Supplementary file 2 — Additional file 2: Overlay_images.zip: Example images showing overlays of blood smear images with detected parasite candidates. [file 12936_2023_4446_MOESM2_ESM.zip › 0006a_1/20210308_093357_Thick_result.jpg]

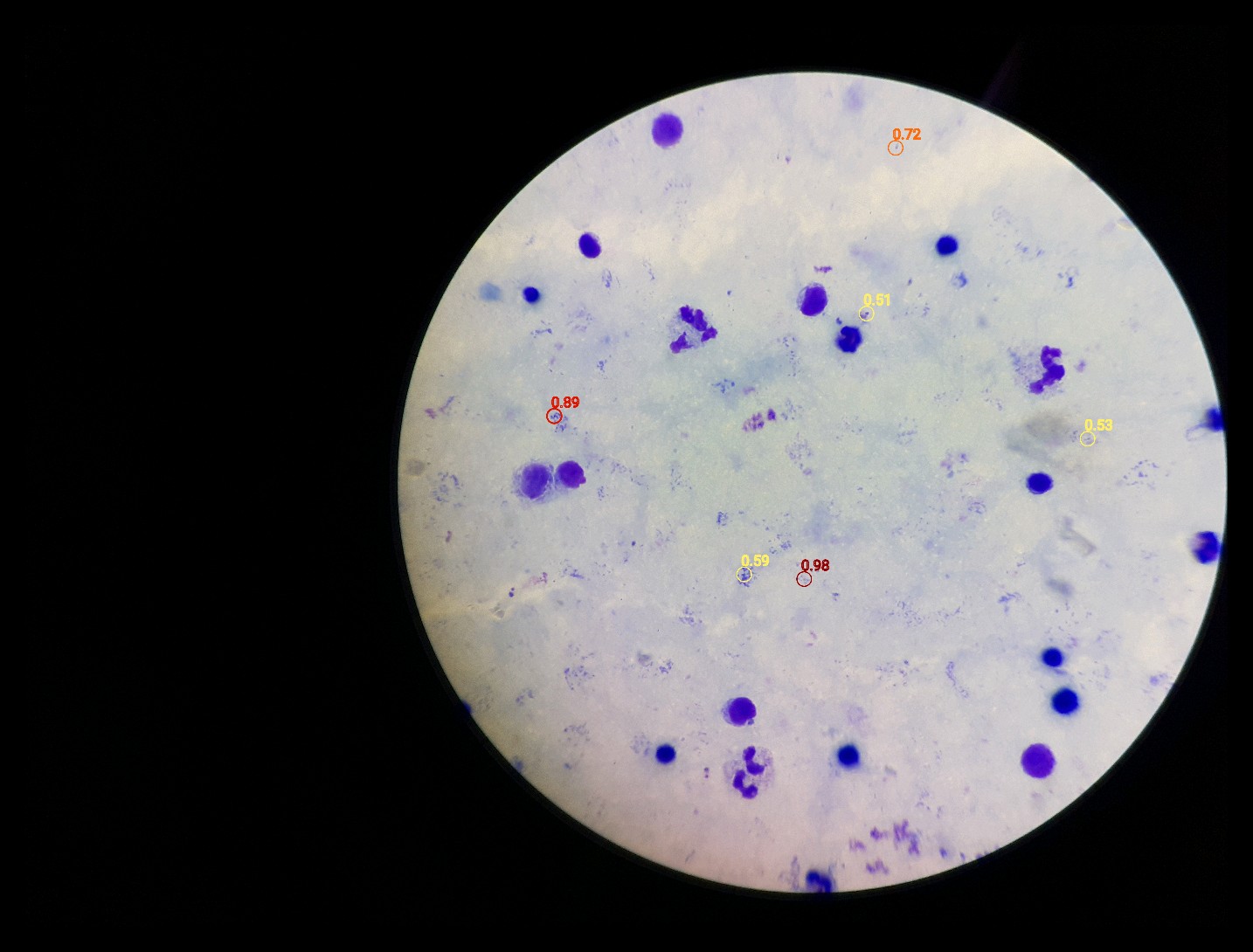

Supplement: Supplementary file 2 — Additional file 2: Overlay_images.zip: Example images showing overlays of blood smear images with detected parasite candidates. [file 12936_2023_4446_MOESM2_ESM.zip › 0006a_1/20210308_093426_Thick_result.jpg]

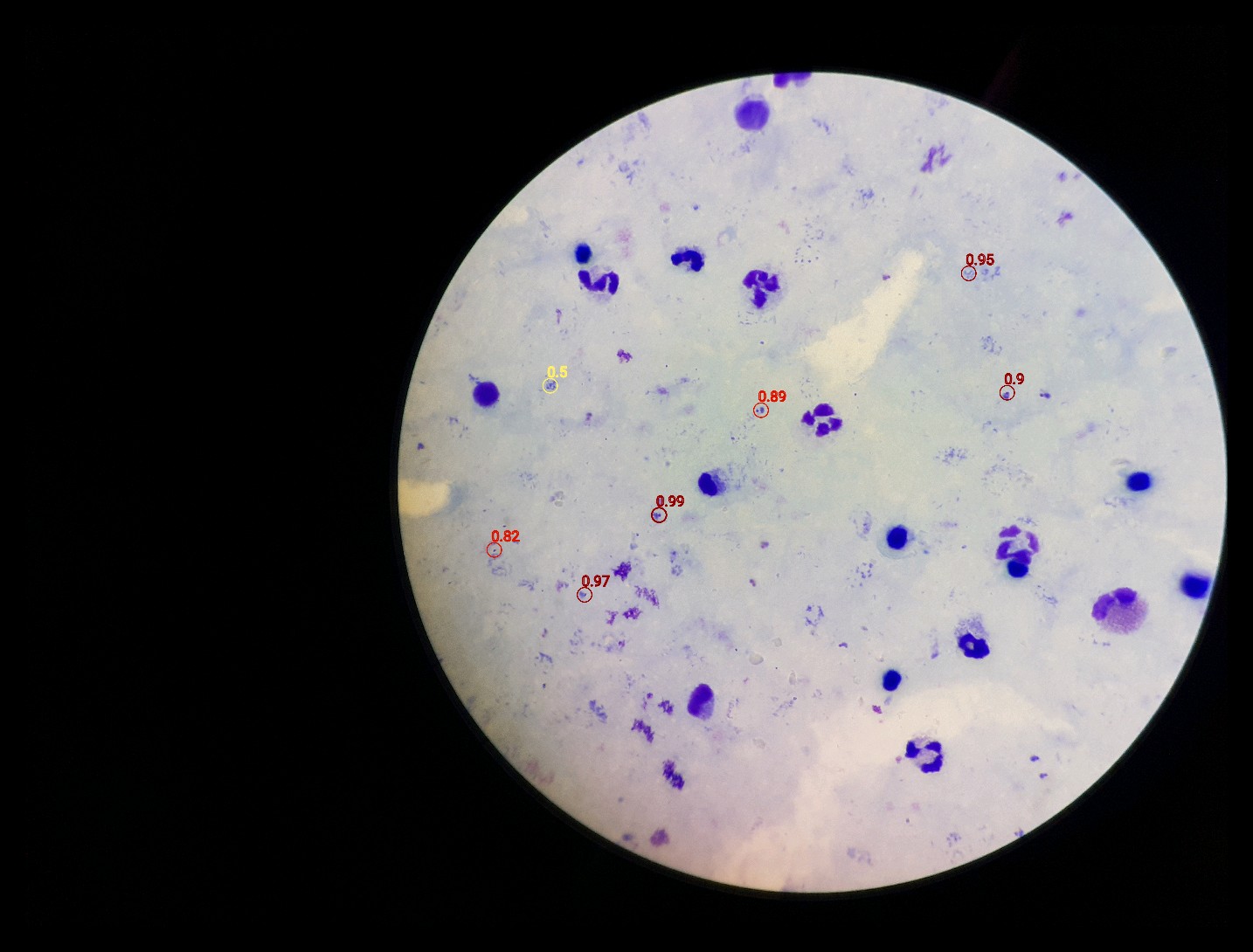

Supplement: Supplementary file 2 — Additional file 2: Overlay_images.zip: Example images showing overlays of blood smear images with detected parasite candidates. [file 12936_2023_4446_MOESM2_ESM.zip › 0006a_1/20210308_093500_Thick_result.jpg]

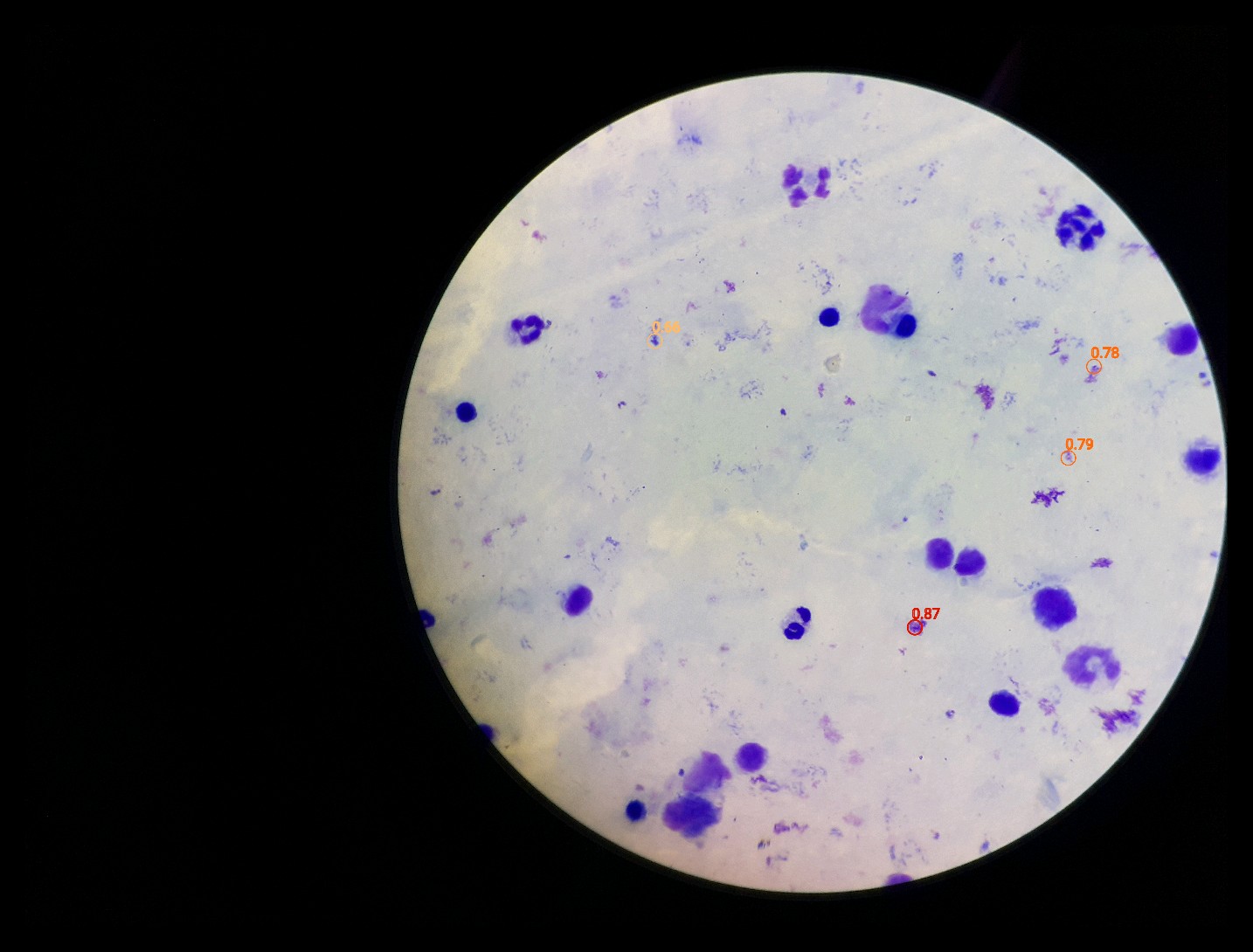

Supplement: Supplementary file 2 — Additional file 2: Overlay_images.zip: Example images showing overlays of blood smear images with detected parasite candidates. [file 12936_2023_4446_MOESM2_ESM.zip › 0006a_1/20210308_093531_Thick_result.jpg]

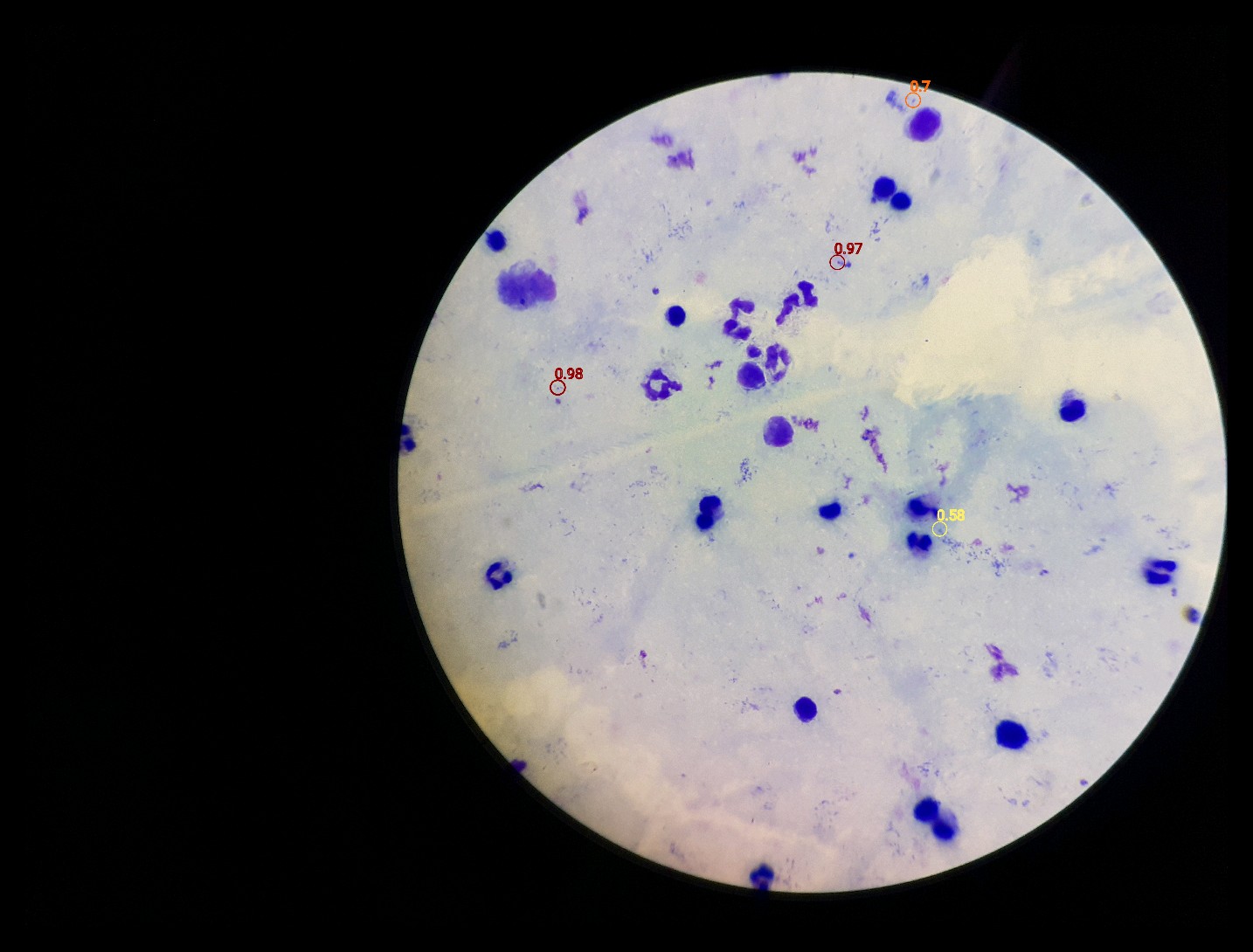

Supplement: Supplementary file 2 — Additional file 2: Overlay_images.zip: Example images showing overlays of blood smear images with detected parasite candidates. [file 12936_2023_4446_MOESM2_ESM.zip › 0006a_1/20210308_093557_Thick_result.jpg]

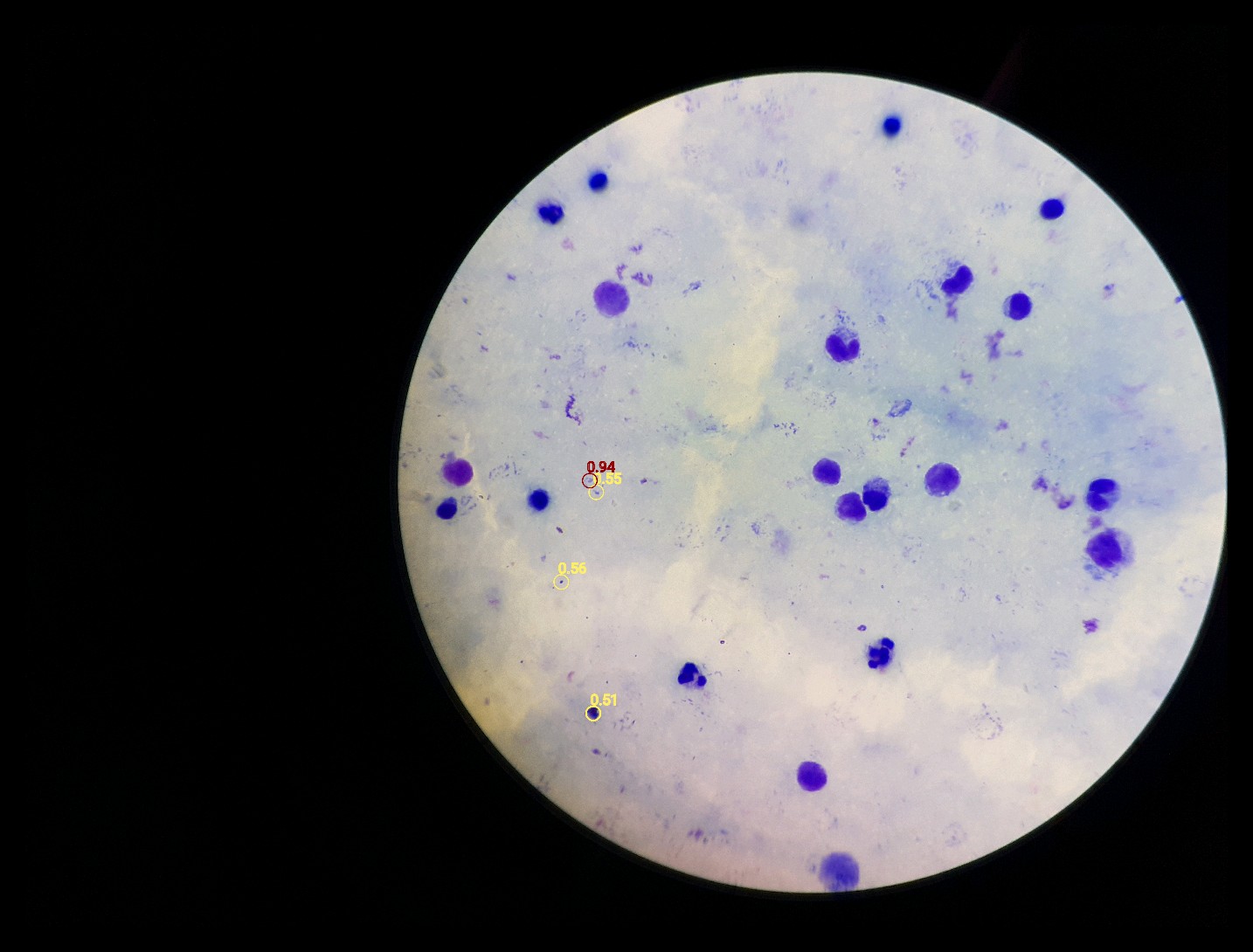

Supplement: Supplementary file 2 — Additional file 2: Overlay_images.zip: Example images showing overlays of blood smear images with detected parasite candidates. [file 12936_2023_4446_MOESM2_ESM.zip › 0006a_1/20210308_093621_Thick_result.jpg]

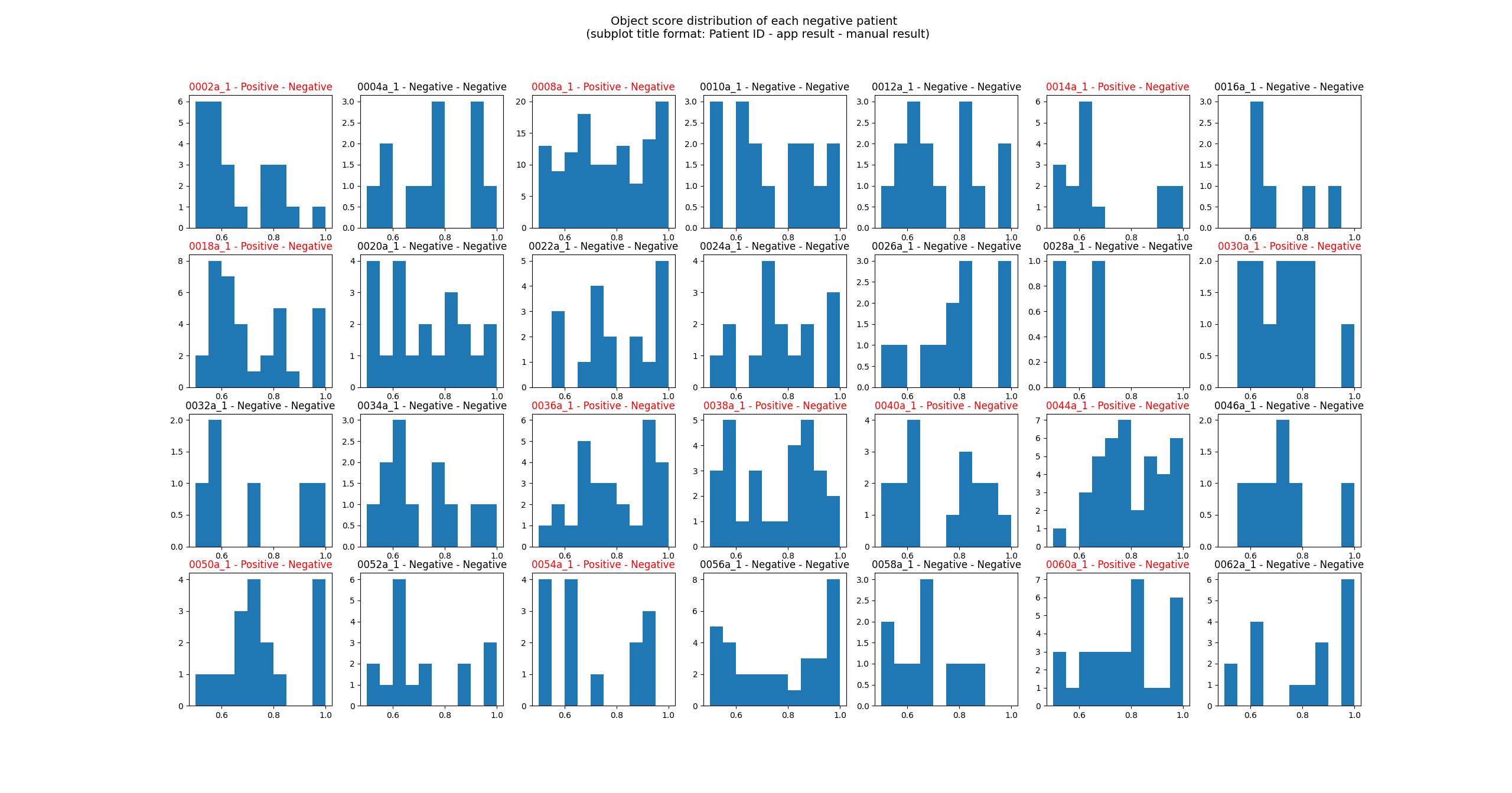

Supplement: Supplementary file 5 — Additional file 5: Object_score_histograms.zip: Object score histograms for each patient. [file 12936_2023_4446_MOESM5_ESM.zip › Figure_negative_1_28.png]

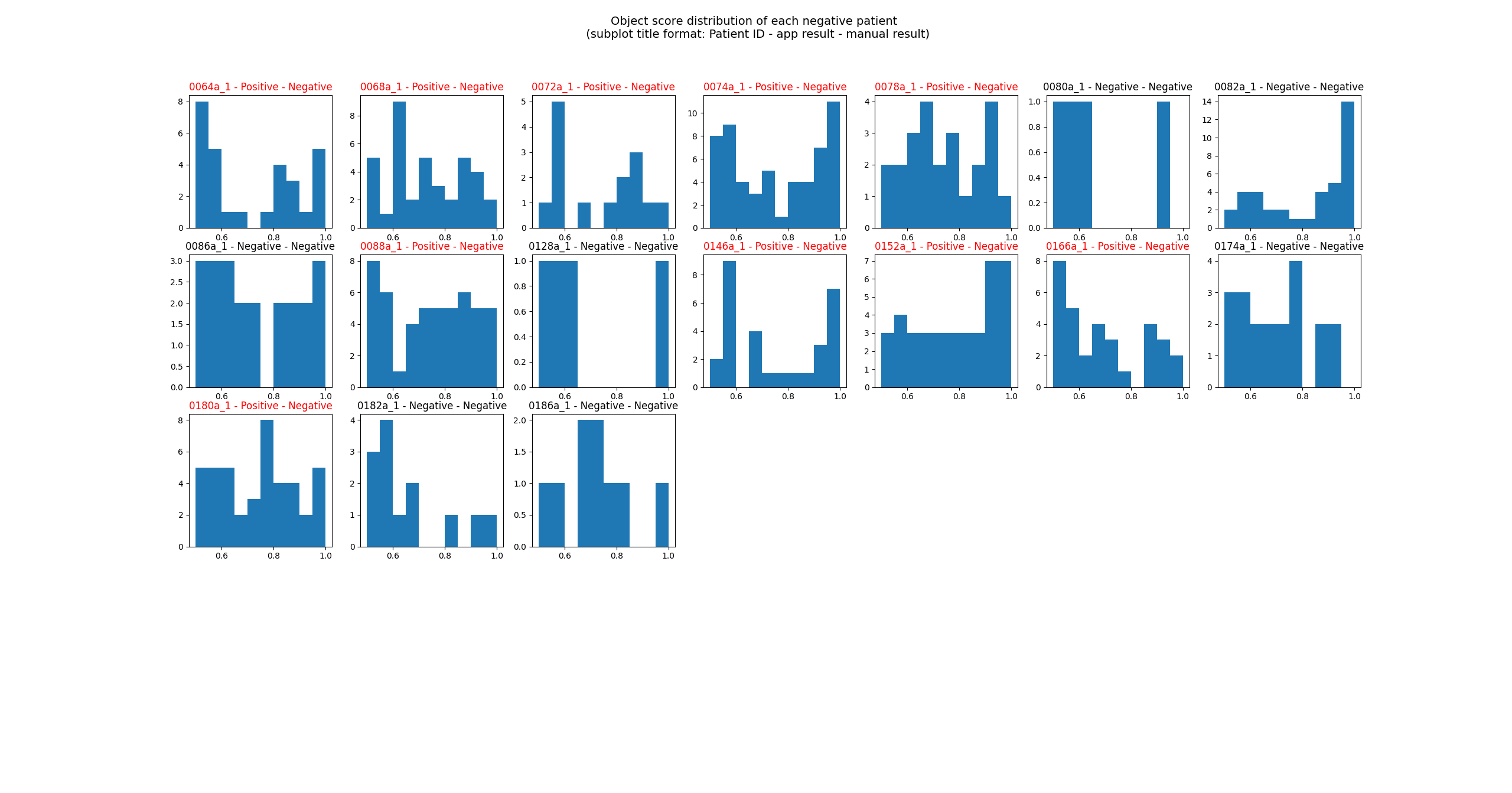

Supplement: Supplementary file 5 — Additional file 5: Object_score_histograms.zip: Object score histograms for each patient. [file 12936_2023_4446_MOESM5_ESM.zip › Figure_negative_28_45.png]

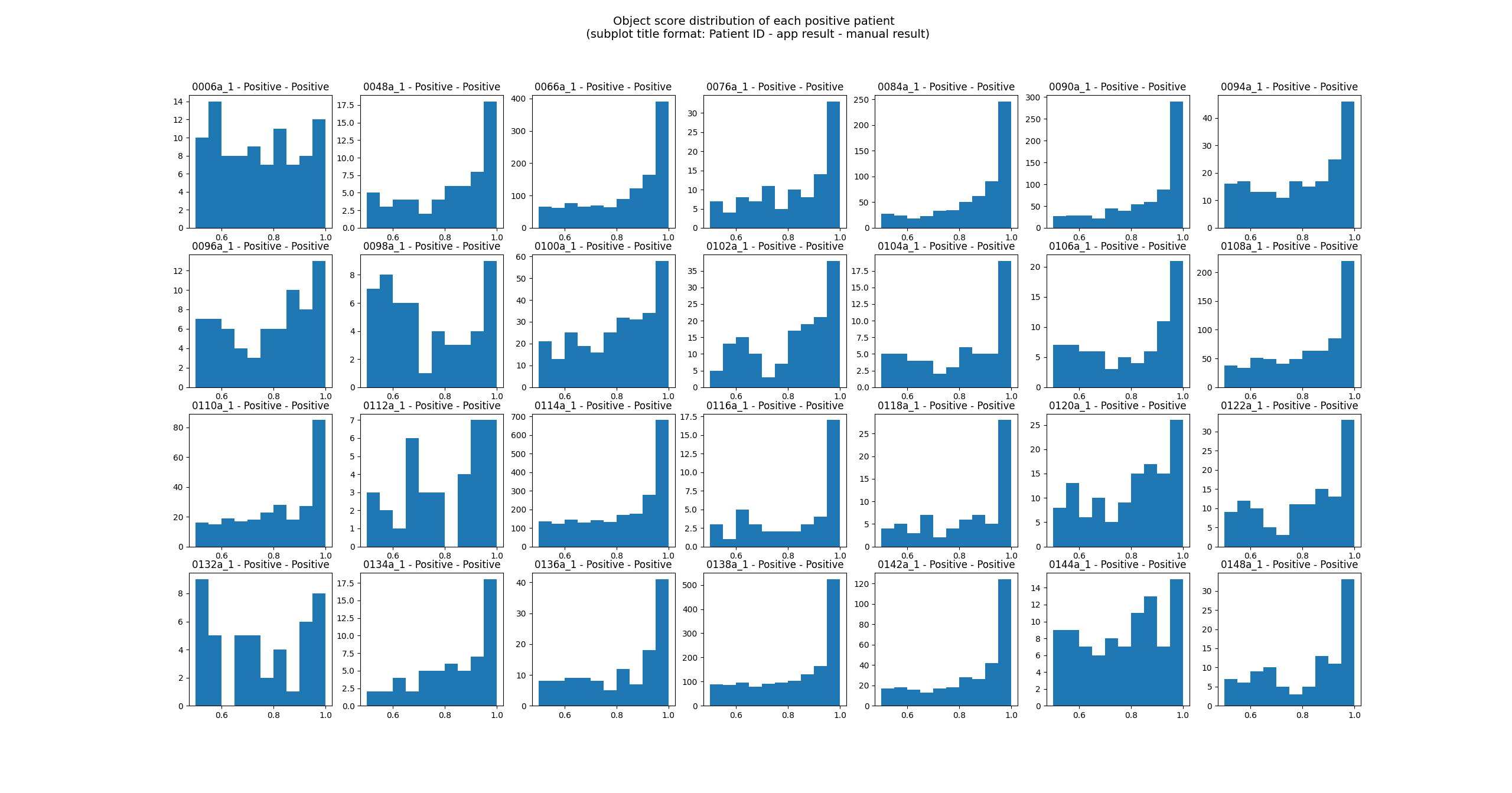

Supplement: Supplementary file 5 — Additional file 5: Object_score_histograms.zip: Object score histograms for each patient. [file 12936_2023_4446_MOESM5_ESM.zip › Figure_positive_1_28.png]

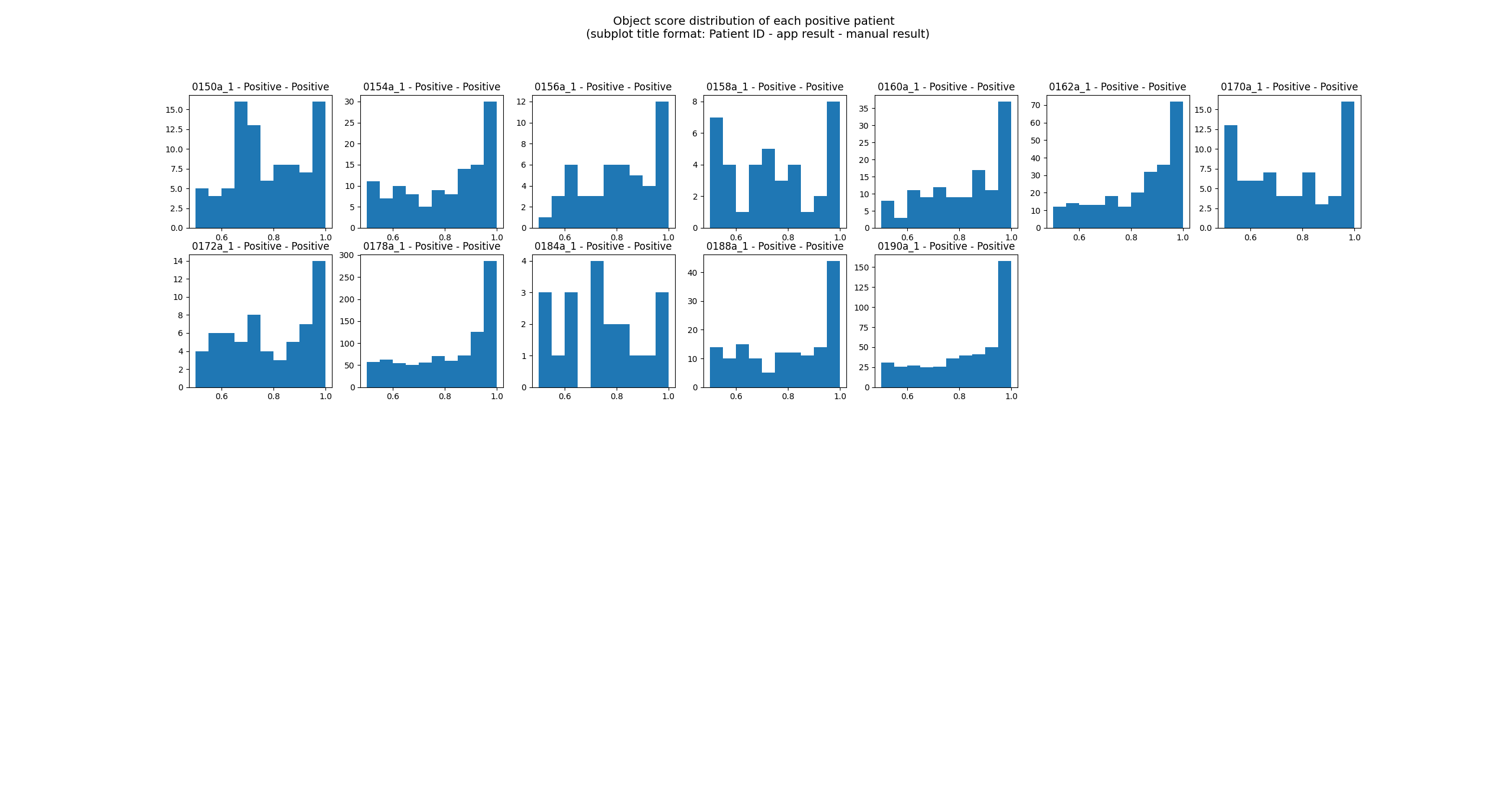

Supplement: Supplementary file 5 — Additional file 5: Object_score_histograms.zip: Object score histograms for each patient. [file 12936_2023_4446_MOESM5_ESM.zip › Figure_positive_29_40.png]
